# Supplementary figures and images for: Understanding the biosynthesis of human IgM SAM-6 through a combinatorial expression of mutant subunits that affect product assembly and secretion
Source: PLoS One. 2024 Jun 7;19(6):e0291568. doi: 10.1371/journal.pone.0291568 (PMC11161108; doi:10.1371/journal.pone.0291568)

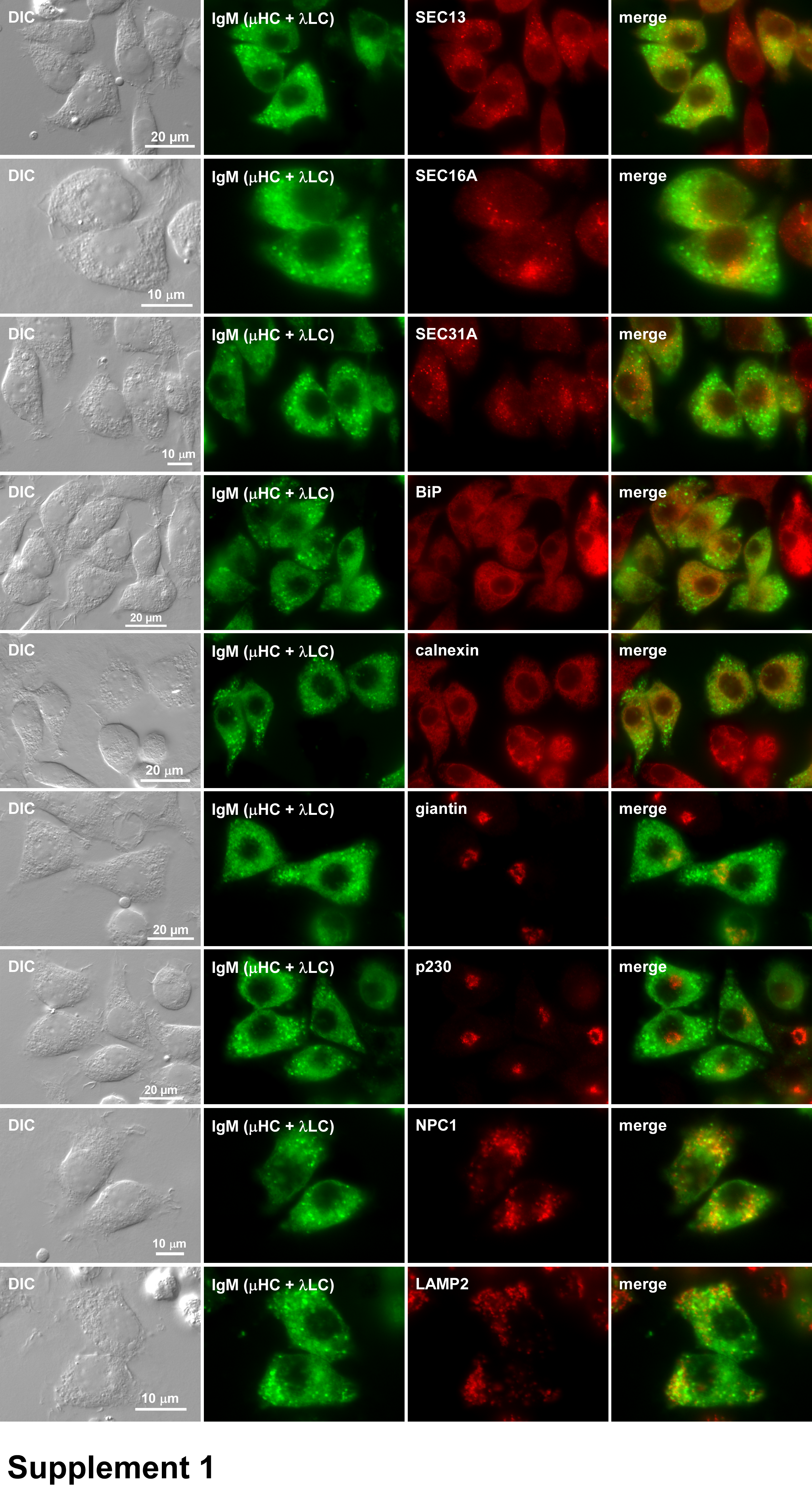

Supplement: S1 Fig — Fluorescent micrographs of HEK293 cells transfected with the [μHC + λLC] construct pair. On day-3 post-transfection, cells were fixed, permeabilized, and co-stained with a 1-to-1 mix of FITC-labeled anti-μHC and FITC-labeled anti-λLC to stain both subunits simultaneously (green) and antibodies against various organelle markers (red). Green and red image fields were superimposed to create ‘merge’ views. (TIF) [file pone.0291568.s001.tif]

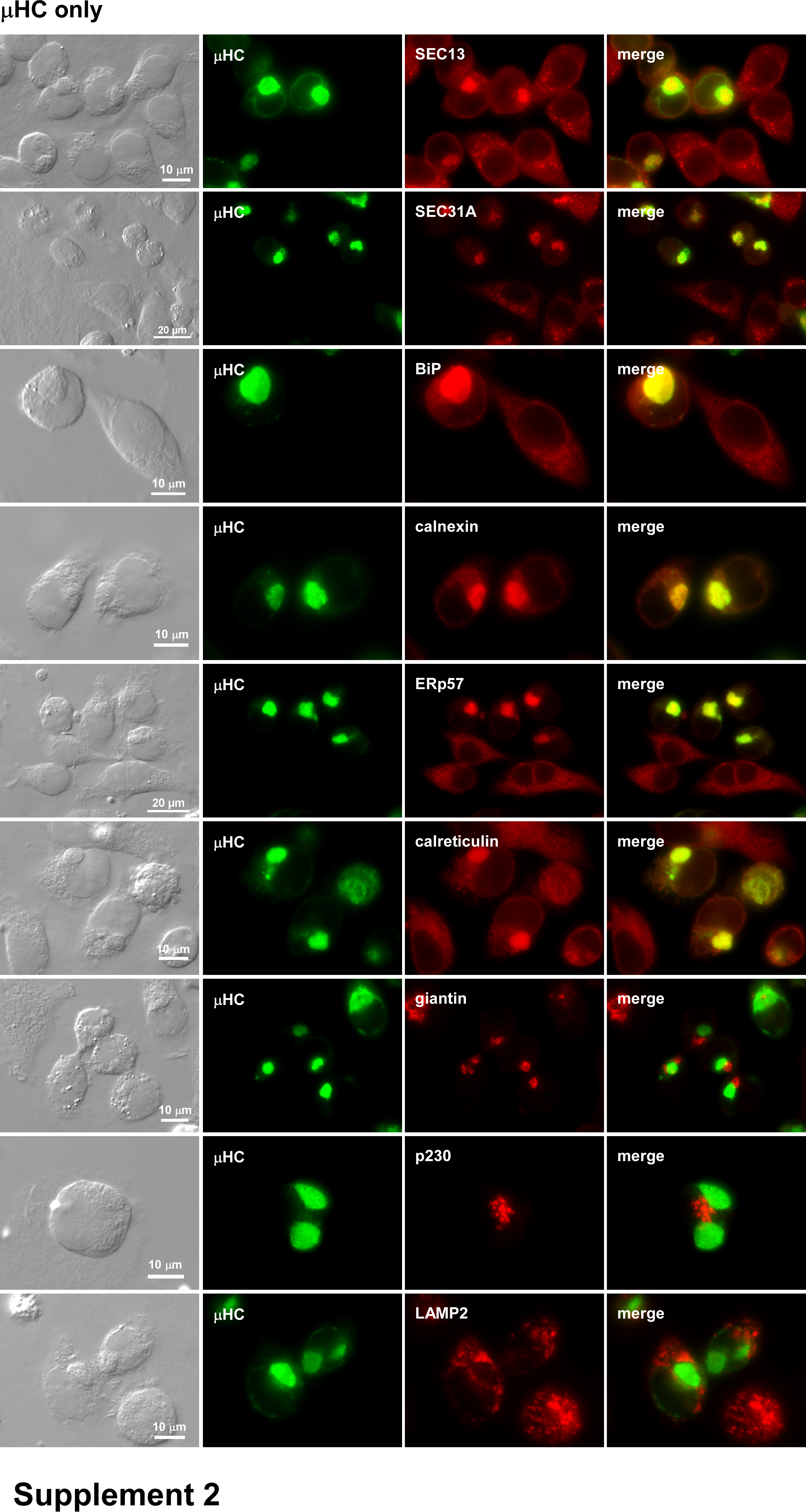

Supplement: S2 Fig — Fluorescent micrographs of HEK293 cells transfected with the μHC construct. On day-3 post-transfection, cells were fixed, permeabilized, and co-stained FITC-labeled anti-μHC (green) and antibodies against various organelle markers (red). Green and red image fields were superimposed to create ‘merge’ views. (TIF) [file pone.0291568.s002.tif]

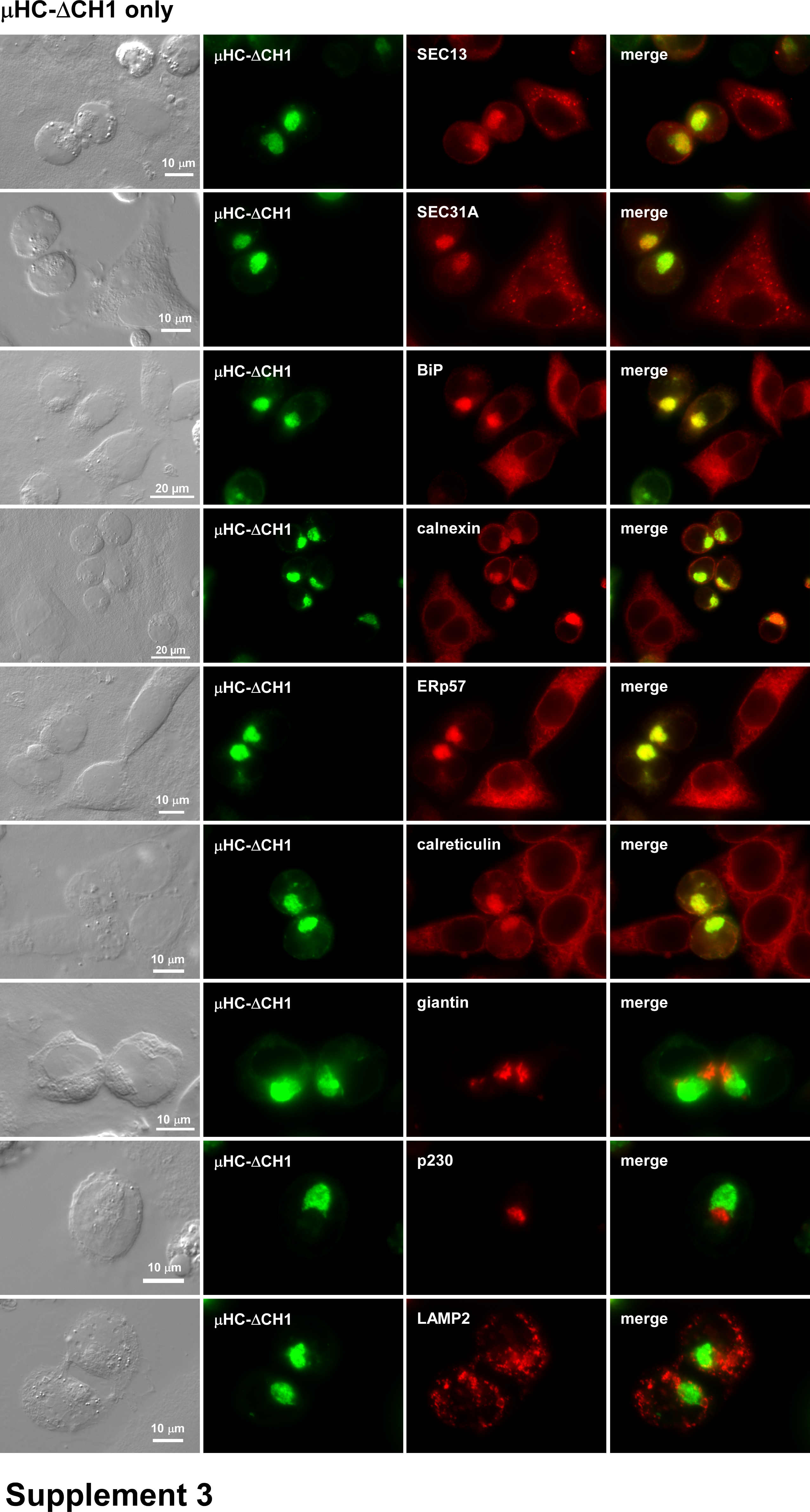

Supplement: S3 Fig — Fluorescent micrographs of HEK293 cells transfected with the μHC-ΔCH1 mutant construct. On day-3 post-transfection, cells were fixed, permeabilized, and co-stained FITC-labeled anti-μHC (green) and antibodies against various organelle markers (red). Green and red image fields were superimposed to create ‘merge’ views. (TIF) [file pone.0291568.s003.tif]

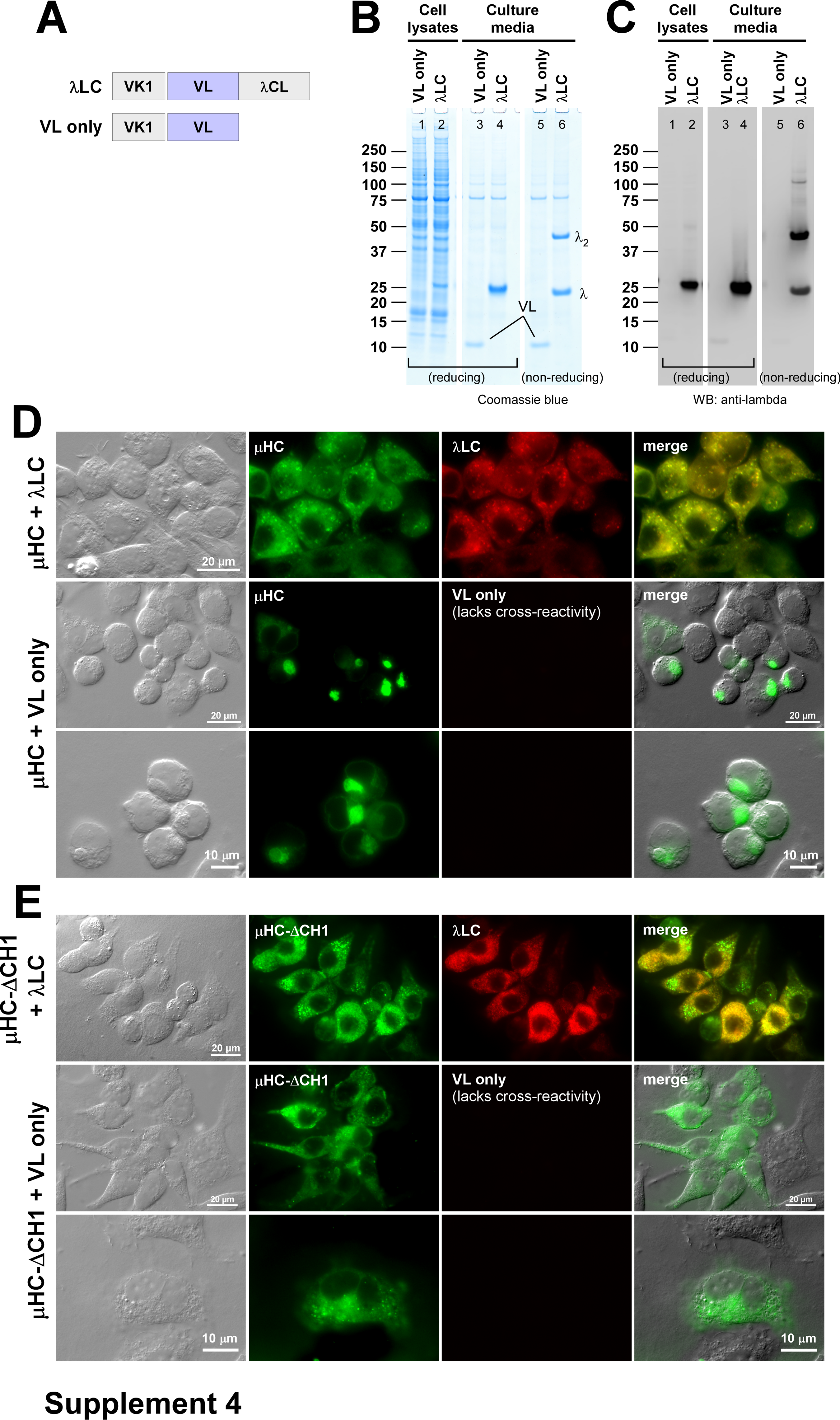

Supplement: S4 Fig — (A) Schematic representation of the VL-only construct. (B, C) HEK293 cells were transfected with VL-only construct (lanes 1, 3, 5) or the full-length λLC (lanes 2, 4, 6). On day-7 post-transfection, cell lysates (lanes 1 and 2) and cell culture media samples (lanes 3–5) were prepared and resolved by SDS-PAGE under reducing or non-reducing conditions followed by Coomassie blue staining (B) or by Western blotting (C). The membrane was probed with polyclonal anti-λLC. The corresponding protein band for the VL-only protein is pointed and labeled in panel B, lanes 3 and 5. Monomeric and dimeric λLC subunit is also labeled next to lane 6. The polyclonal anti-λLC raised against the constant domain of λLC could not recognize the VL-only protein in Western blotting. (D, E) Fluorescent micrographs of HEK293 cells transfected with the constructs shown on the left side. On day-3 post-transfection, cells were fixed, permeabilized, and co-stained with FITC-labeled anti-μHC and Texas Red-labeled anti-λLC. The polyclonal anti-λLC was raised against the constant domain of λLC and could not recognize the VL-only protein. (TIF) [file pone.0291568.s004.tif]

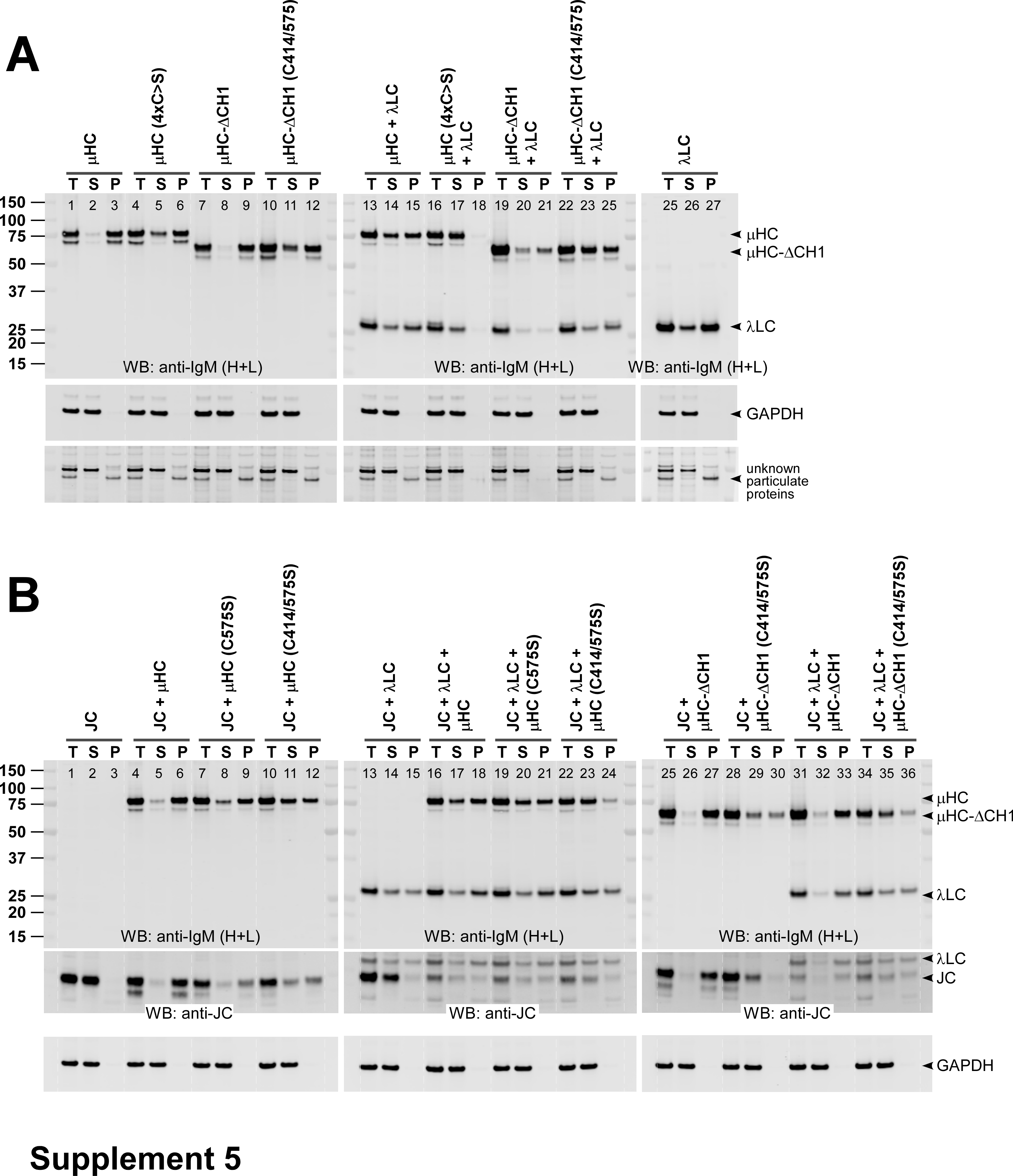

Supplement: S5 Fig — (A) On day-3 post-transfection, the detergent solubility was determined for the intracellular pool of transfected μHC and/or λLC subunit. The total detergent cell extracts were prepared under non-denaturing conditions and subjected to 15,000 g centrifugation for 60 min. Then, total (T), soluble (S), and particulate (P) fractions were resolved in SDS-PAGE under reducing conditions. Transfected construct(s) are shown at the top of corresponding lanes. Membranes were probed with polyclonal anti-IgM (H+L) (top panel) and monoclonal anti-GAPDH (second panel). Unidentified, non-specifically cross-reacting proteins are also shown in the bottom panel as the reference for soluble and particulate proteins. (B) The effect of co-expression partners on the detergent solubility of JC subunit was determined. Membranes were probed with polyclonal anti-IgM (H+L) (top panel), monoclonal anti-JC (middle panel), and monoclonal anti-GAPDH (third panel). (TIF) [file pone.0291568.s005.tif]

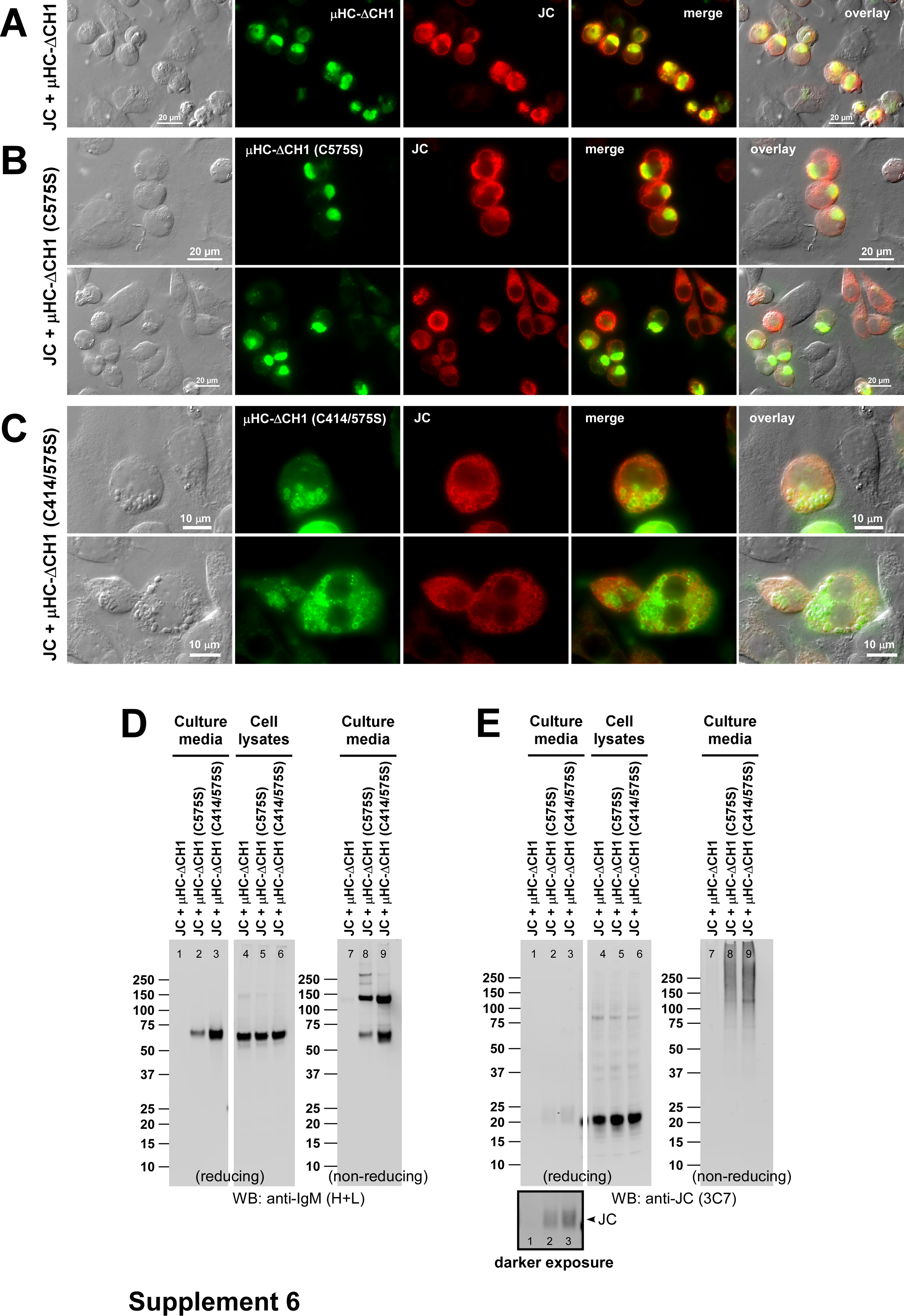

Supplement: S6 Fig — Fluorescent micrographs of HEK293 cells co-transfected with JC and one of the following constructs: (A) μHC-ΔCH1, (B) μHC-ΔCH1 (C575S), or (C) μHC-ΔCH1 (C414/575S). Co-transfected construct pairs are also shown on the left side of each row. On day-3 post-transfection, cells were fixed, permeabilized, and co-stained with FITC-labeled anti-μHC and monoclonal anti-JC (shown in red). (D) On day-7 post-transfection, cell culture media (lanes 1‒3; lanes 7‒9) and cell lysates (lanes 4‒6) were analyzed by Western blotting. Membranes were probed with polyclonal anti-IgM (H+L). Co-transfected construct pairs are shown at the top of each lane. (E) The same culture media and cell lysate samples were analyzed by Western blotting using monoclonal anti-JC. A longer exposed Western blot result is shown underneath the corresponding lanes in a black box for the cell culture media (panel E, lanes 1‒3). (TIF) [file pone.0291568.s006.tif]

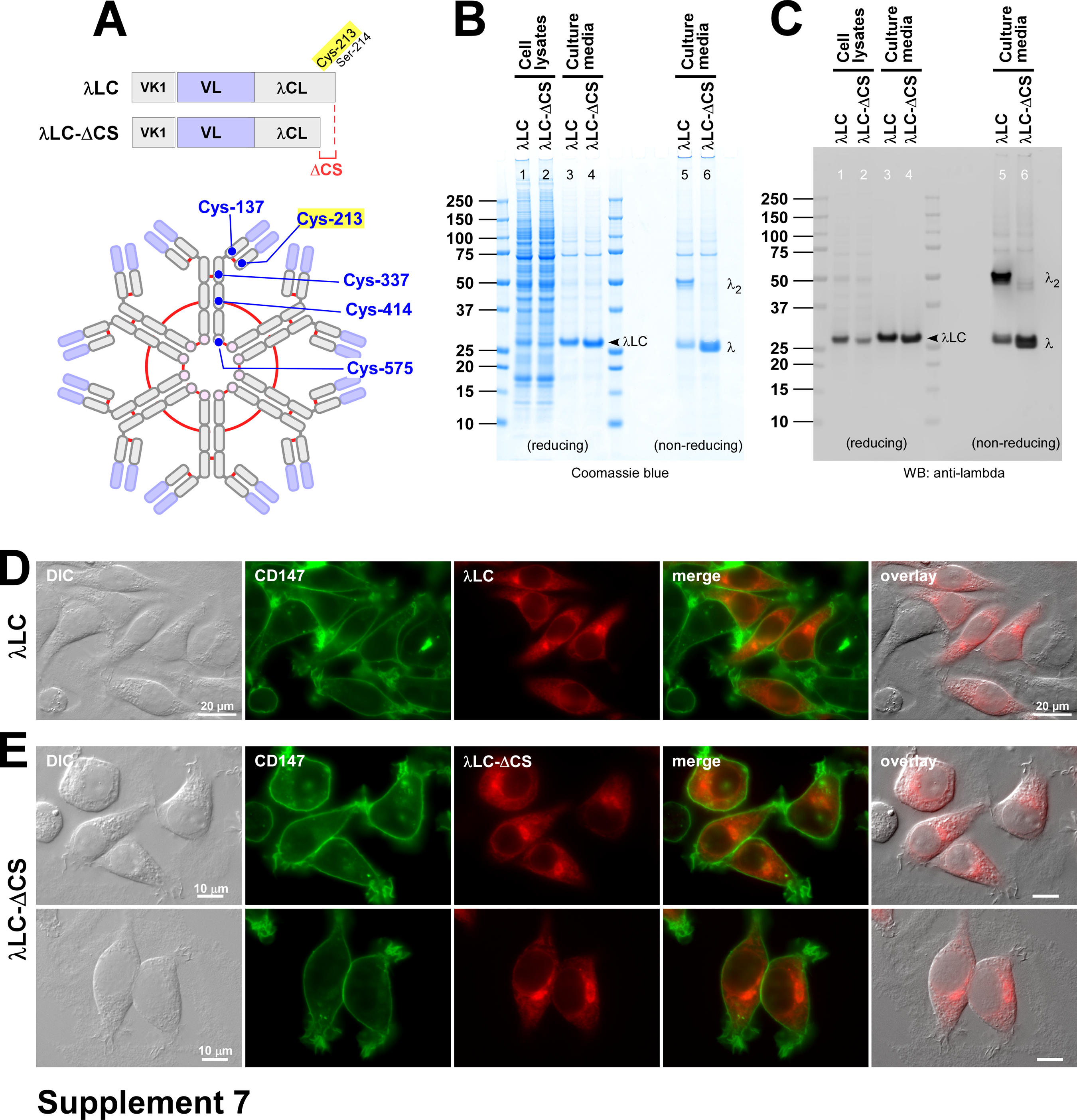

Supplement: S7 Fig — (A, top) Schematic representation of the full-length SAM-6 λLC (top row) and its ΔCS mutant (second row) in which two C-terminal amino acids (Cys-213 and Ser-214) are deleted. (A, bottom) The position of Cys-213 residue involved in the HC‒LC inter-chain disulfide bond is highlighted in yellow in the context of hexameric IgM. Solid red lines represent the inter-chain disulfide bond connectivity. (B, C) HEK293 cells were transfected with full-length λLC (lanes 1, 3, 5) or its ΔCS mutant (lanes 2, 4, 6). On day-7 post-transfection, cell lysates (lanes 1 and 2) and cell culture media samples (lanes 3 and 4) were prepared and resolved by SDS-PAGE under reducing conditions followed by Coomassie blue staining (B) or by Western blotting (C). The day-7 cell culture media were also analyzed by Coomassie staining or Western blotting after resolving the proteins under non-reducing conditions (B C, lanes 5 and 6). Membranes were probed with polyclonal anti-λLC. The corresponding protein band for the λLC subunit is pointed by an arrowhead and labeled. Monomeric and dimeric λLC subunit is labeled next to lane 6. (D, E) Fluorescent micrographs of HEK293 cells transfected with full-length λLC (D) or ΔCS mutant (E). On day-3 post-transfection, cells were fixed, permeabilized, and co-stained with FITC-labeled anti-CD147 and Texas Red-labeled anti-λLC. (TIF) [file pone.0291568.s007.tif]

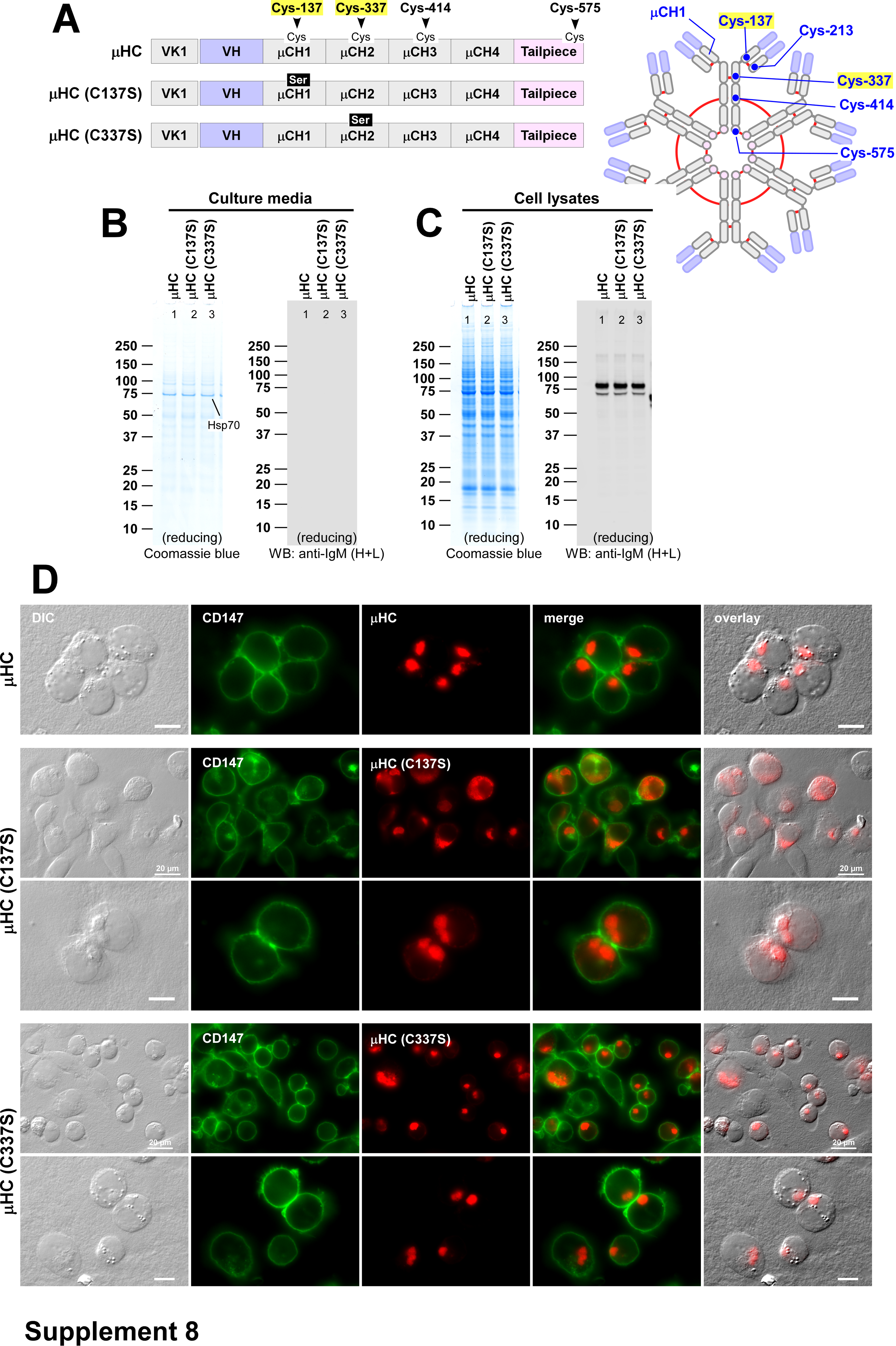

Supplement: S8 Fig — (A, left) Schematic representation of parental SAM-6 μHC (top row) and its C137S and C337S mutants (second and third rows). (A, right) The position of Cys-137 and Cys-337 residues is highlighted in yellow in the context of hexameric IgM. Solid red lines represent the inter-chain disulfide bond connectivity. (B, C) HEK293 cells were transfected with parental μHC and its mutants, as shown at the top of each lane. On day-7 post-transfection, cell culture media (B) and cell lysates (C) were prepared and resolved by SDS-PAGE under reducing conditions followed by Coomassie blue staining (B, C, left panels) or by Western blotting (B, C, right panels). Membranes in B and C were probed with polyclonal anti-IgM (H+L). Both parental and mutant μHCs were completely retained in the cells and failed to secrete. (D) Fluorescent micrographs of HEK293 cells transfected with parental μHC (top row), μHC (C137S) mutant (second and third rows), or μHC (C337S) mutant (fourth and fifth rows). On day-3 post-transfection, cells were fixed, permeabilized, and co-stained with FITC-labeled anti-CD147 and Texas Red-labeled anti-μHC. (TIF) [file pone.0291568.s008.tif]

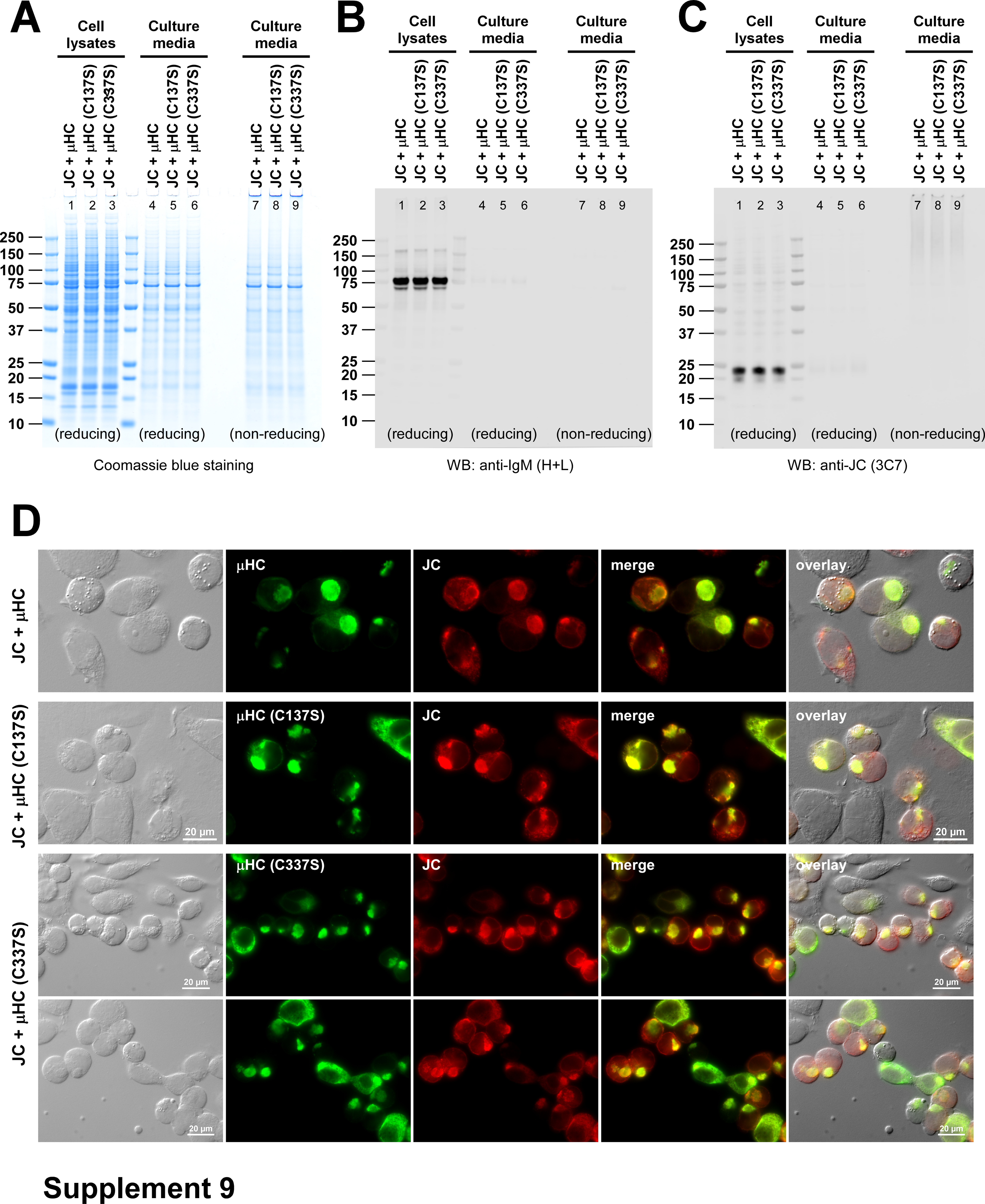

Supplement: S9 Fig — (A‒C) HEK293 cells were co-transfected with JC and one of the following μHC constructs: parental μHC (lanes 1, 4, 7), μHC (C137S) (lanes 2, 5, 8), and μHC (C337S) (lanes 3, 6, 9). On day-7 post-transfection, cell lysate samples were prepared (lanes 1‒3), and cell culture media were harvested (lanes 4‒9) to run SDS-PAGE under reducing conditions (lanes 1‒6) or non-reducing conditions (lanes 7‒9) followed by Coomassie blue staining (panel A) and Western blotting (panels B and C). Membranes were probed with (B) polyclonal anti-IgM (H+L) or (C) monoclonal anti-JC. A co-transfected construct pair is shown at the top of each lane. (D) Fluorescent micrographs of HEK293 cells co-transfected with JC and parental μHC (top row), JC and μHC (C137S) (second row), and JC and μHC (C337S) (third and fourth rows). On day-3 post-transfection, cells were fixed, permeabilized, and co-stained with FITC-labeled anti-μHC (green) and monoclonal anti-JC (red). (TIF) [file pone.0291568.s009.tif]

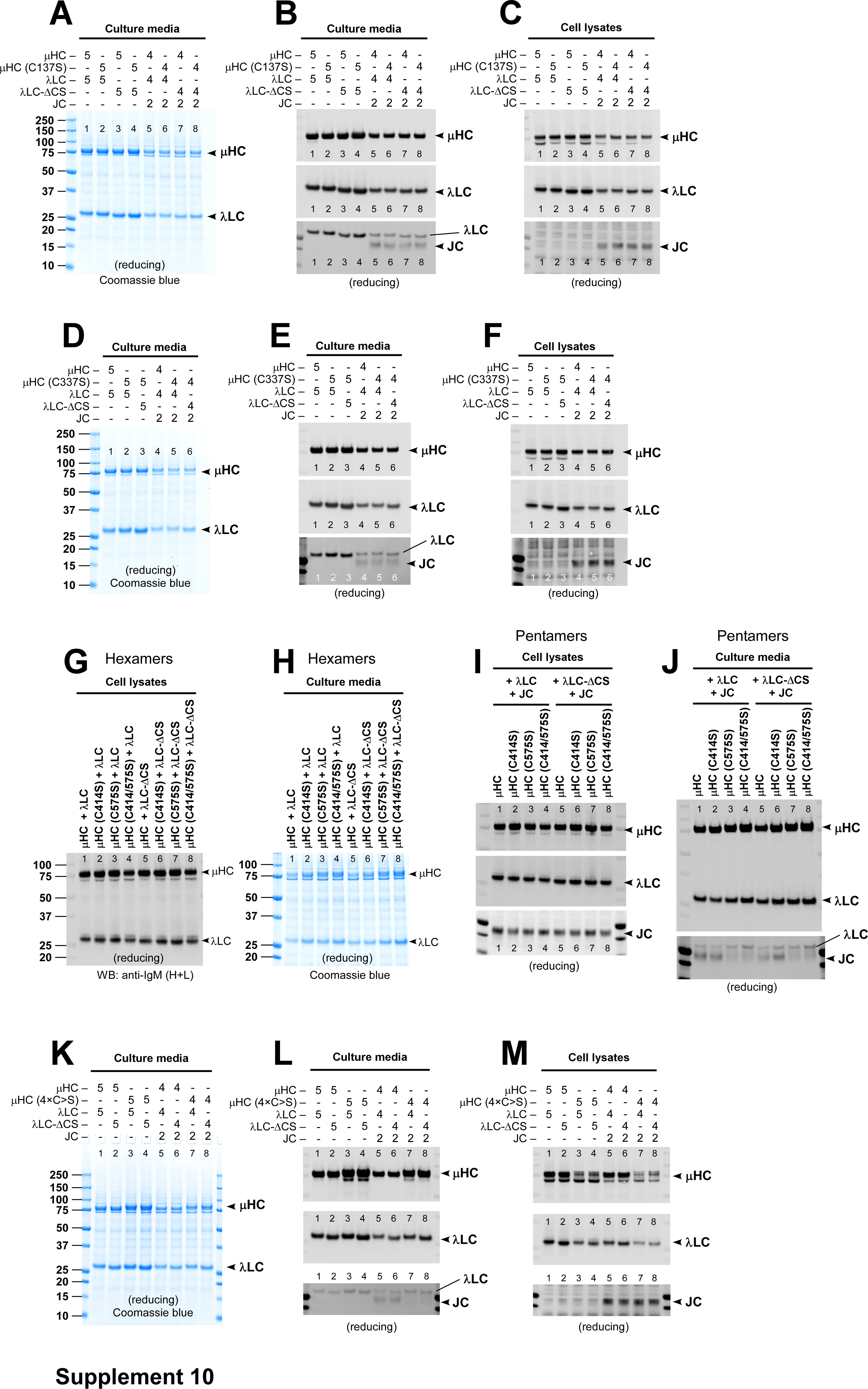

Supplement: S10 Fig — (A‒C) The effect of μHC (C137S) mutant subunit on IgM expression and secretion was assessed both in a 2-chain co-expression (lanes 1‒4) and a 3-chain co-expression (lanes 5‒8) settings. The DNA ratio of each subunit chain is shown at the top of each lane. On day-7 post-transfection, cell culture media (A, B) and cell lysates (C) were resolved by SDS-PAGE under reducing conditions followed by Coomassie blue staining (A) or Western blotting (B, C). Membranes were probed with polyclonal anti-IgM (H+L) to detect μHC or μHC (C137S) (top panel) and λLC or λLC-ΔCS (second panel) or with monoclonal anti-JC (third panel). (D‒F) The effect of C337S mutation on IgM expression was tested both in a 2-chain (lanes 1‒3) and a 3-chain co-expression (lanes 4‒6) settings. The DNA ratio of each subunit chain is shown at the top of each lane. On day-7 post-transfection, cell culture media (D, E) and cell lysates (F) were resolved by SDS-PAGE under reducing conditions, followed by Coomassie blue staining and Western blotting. Membranes were probed with polyclonal anti-IgM (H+L) to detect μHC or μHC (C337S) (E, F; top panel) and λLC or λLC-ΔCS (E, F, second panel) or with monoclonal anti-JC (E, F, third panel). (G‒J) The effect of μHC (C414S) mutant on polymeric IgM expression was tested both in a 2-chain (panel G, H) and 3-chain co-expression (panel I) settings. To detect any subtle effects, μHC (C414S) was compared side by side with μHC (C575S) and μHC (C414/575S). On day-7 post-transfection, cell culture media (H, J) and cell lysates (G, I) were resolved by SDS-PAGE under reducing conditions, followed by Coomassie blue staining (H) and Western blotting (G, I, J). Membranes were probed with polyclonal anti-IgM (H+L) to detect μHC variants and λLC variants or monoclonal anti-JC (I, J; bottom). (K‒M) The effect of μHC (4×C>S) mutant on IgM expression and secretion was tested both in a 2-chain (lanes 1‒4) and a 3-chain co-expression (lanes 5‒8) settings. The DNA ratio of each subunit chai [file pone.0291568.s010.tif]

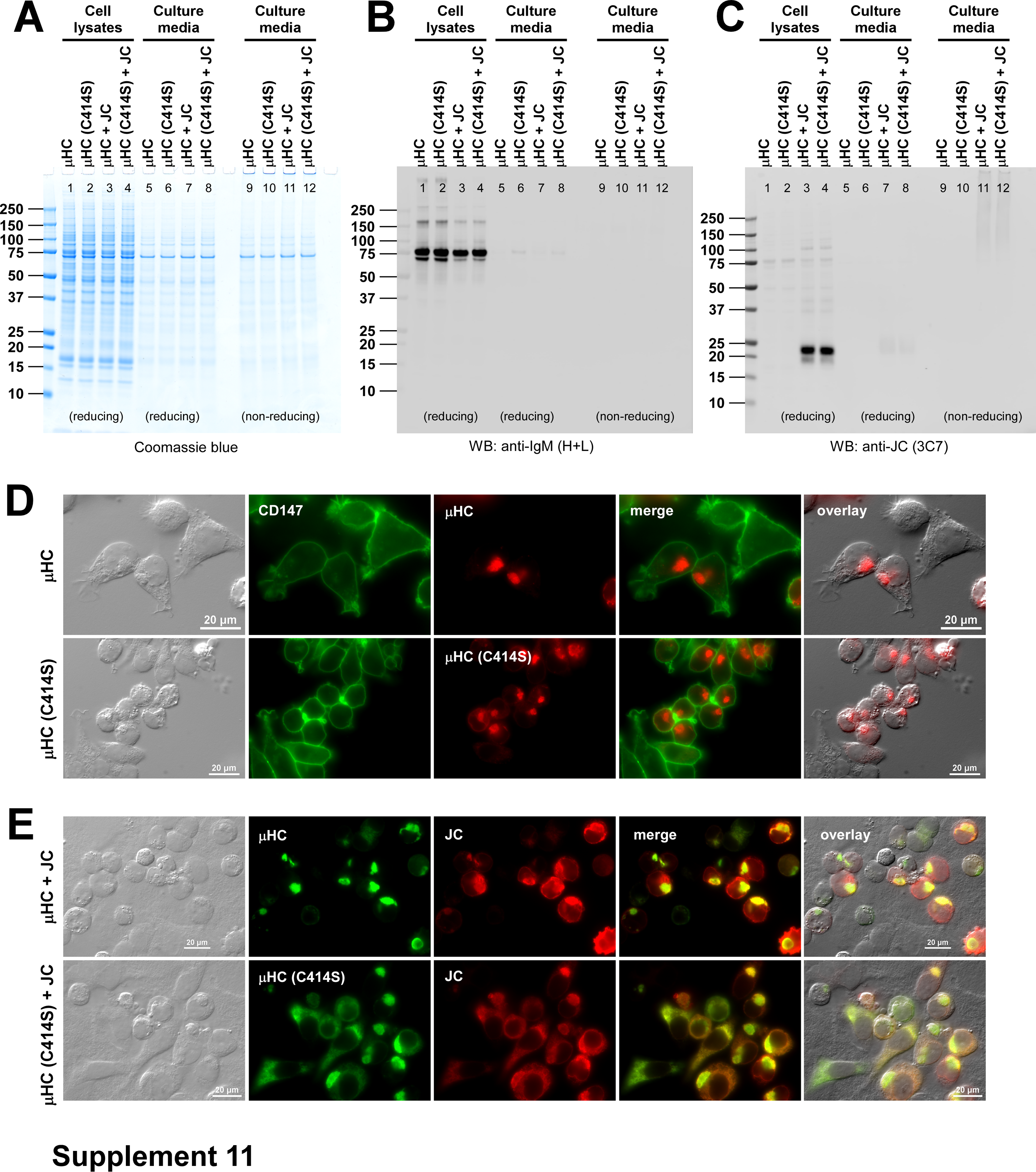

Supplement: S11 Fig — (A–C) HEK293 cells were transfected with the construct or construct pair shown at the top of each lane. On day-7 post-transfection, cell culture media (lanes 5–12) and cell lysates (lanes 1–4) were resolved by SDS-PAGE under reducing (lanes 1–8) or non-reducing (lanes 9–12) conditions followed by Coomassie blue staining (A) or by Western blotting (B, C). Membranes in B and C were probed with polyclonal anti-IgM (H+L) and monoclonal anti-JC, respectively. (D) Fluorescent micrographs of HEK293 cells transfected with parental μHC (top row) or μHC (C414S) mutant (second row). On day-3 post-transfection, cells were fixed, permeabilized, and co-stained with FITC-labeled anti-CD147 and Texas Red-labeled anti-μHC. (E) Fluorescent micrographs of HEK293 cells transfected with [μHC + JC] pair (top row) or [μHC (C414S) + JC] pair (second row). Cells were co-stained with FITC-labeled anti-μHC (green) and monoclonal anti-JC (shown in red). (TIF) [file pone.0291568.s011.tif]

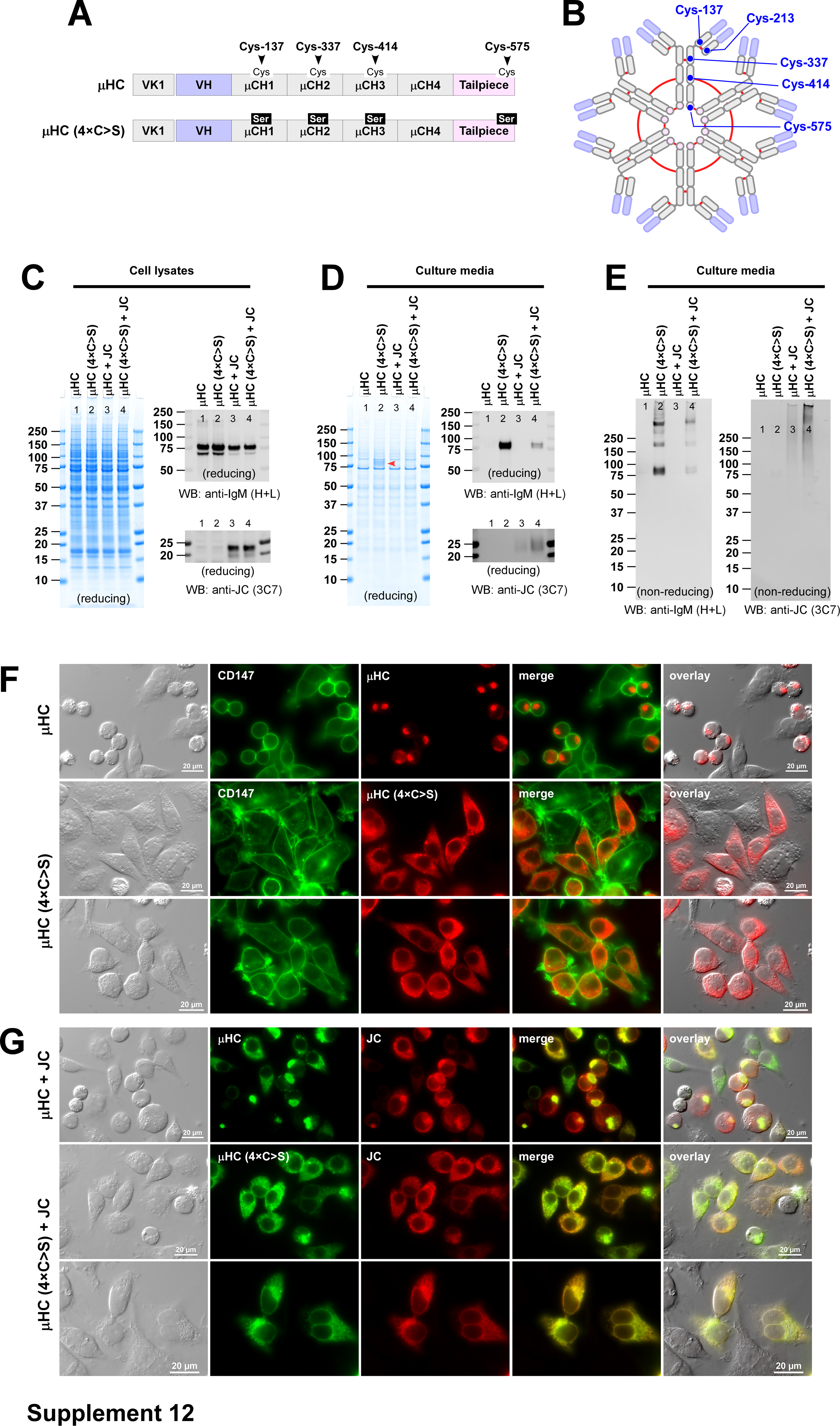

Supplement: S12 Fig — (A) Schematic representation of parental SAM-6 μHC (top row) and its 4×C>S mutant (second row). (B) The position of all Cys residues involved in inter-chain disulfide bond formation on μHC and λLC are depicted in the context of hexameric IgM. Solid red lines represent the inter-chain disulfide bond connectivity. (C, D) HEK293 cells were transfected with parental μHC alone (lane 1) or its 4×C>S mutant alone (lane 2). Likewise, the cells are co-transfected with [μHC + JC] pair (lane 3) or [μHC (4×C>S) + JC] pair (lane 4). On day-7 post-transfection, cell lysates (C) and culture media (D) were resolved by SDS-PAGE under reducing conditions followed by Coomassie blue staining (C, D; left panel) or by Western blotting (C, D; right panels). Membranes in C and D were probed with polyclonal anti-IgM (H+L) (top panel) or monoclonal anti-JC (bottom panel). (E) Day-7 culture media were also analyzed by Western blotting after proteins were resolved under non-reducing conditions. Membranes were probed with polyclonal anti-IgM (H+L) (left panel) or monoclonal anti-JC (right panel). (F) Fluorescent micrographs of HEK293 cells transfected with parental μHC (top row) or μHC (4×C>S) mutant (second and third rows). On day-3 post-transfection, cells were fixed, permeabilized, and co-stained with FITC-labeled anti-CD147 and Texas Red-labeled anti-μHC. (G) Fluorescent micrographs of HEK293 cells co-transfected with [μHC + JC] pair (top row) or [μHC (4×C>S) + JC] pair (second and third rows). Cells were co-stained FITC-labeled anti-μHC (green) and monoclonal anti-JC (shown in red). (TIF) [file pone.0291568.s012.tif]

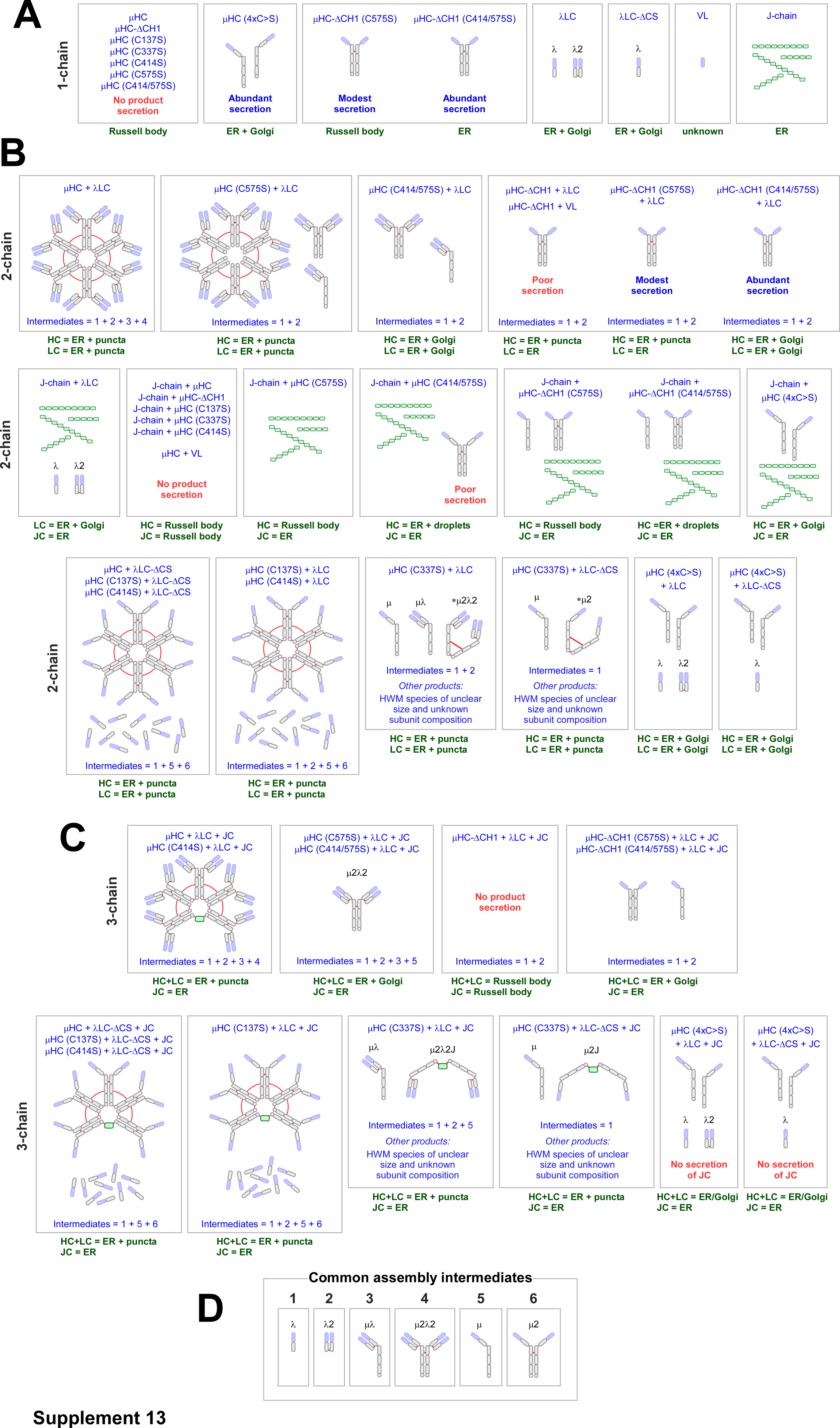

Supplement: S13 Fig — The types of secreted main products and significant by-products released to culture media are illustrated. (A) single-construct expression setting. (B) 2-chain co-expression setting. (C) 3-chain co-expression setting. The name of the transfected construct (or a set of constructs) is shown in blue letters at the upper-most area of each box. At the lower-most area, common types of assembly intermediate in each condition are shown using the numbering system categorized in panel D. Steady state subcellular distribution of individual subunits for each transfection setting is shown in the space under each box, in green letters. When there is no mention of the secretion outputs, it suggests that the products were secreted abundantly. (D) Six commonly produced assembly intermediates released to the culture media are illustrated in each box, from 1 to 6. Solid red lines represent the inter-chain disulfide bond connectivity. (TIF) [file pone.0291568.s013.tif]
