# Supplementary material for: Understanding the biosynthesis of human IgM SAM-6 through a combinatorial expression of mutant subunits that affect product assembly and secretion
Source: PLoS One. 2024 Jun 7;19(6):e0291568. doi: 10.1371/journal.pone.0291568 (PMC11161108; doi:10.1371/journal.pone.0291568)

Figure 1 D and E: The raw gel and blot before the cropping are shown right. The cropped regions are shown in red box.

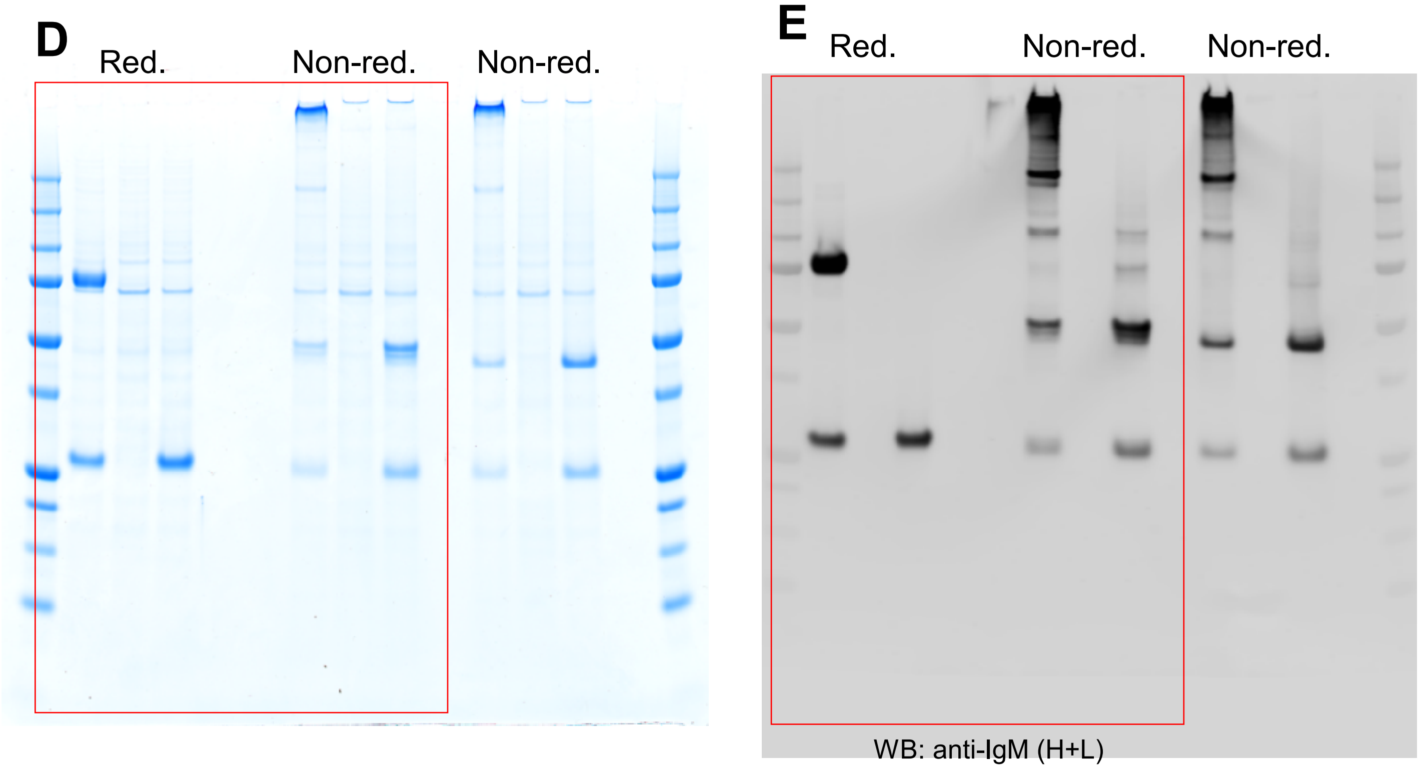

Figure 1 F: The original gel and blot corresponding to Fig. 1F before the lane cropping are shown right. The lanes marked by X contains samples not directly related to the Figure and were cropped.

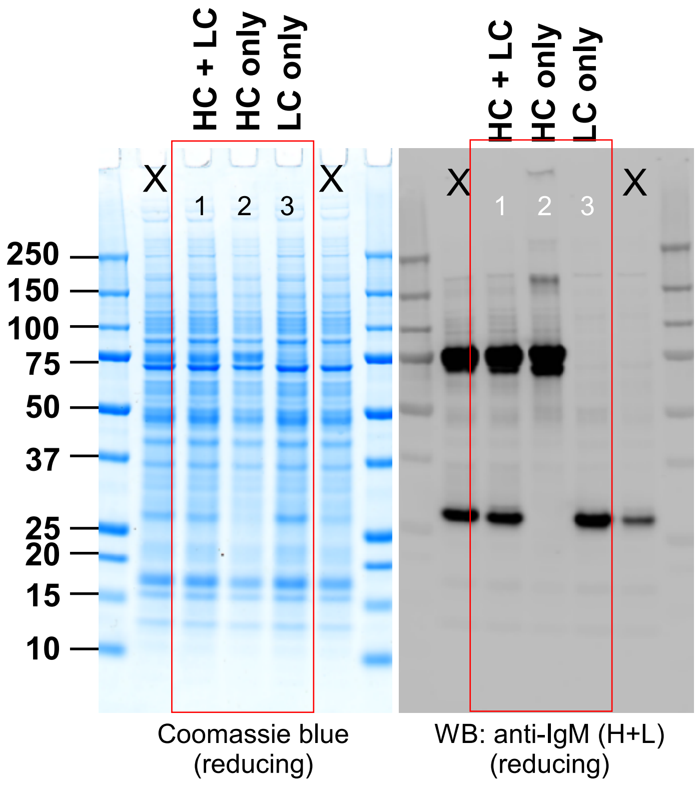

Figure 1 G: The original blots used to generate Fig. 1G are shown below. Continue to the next page.

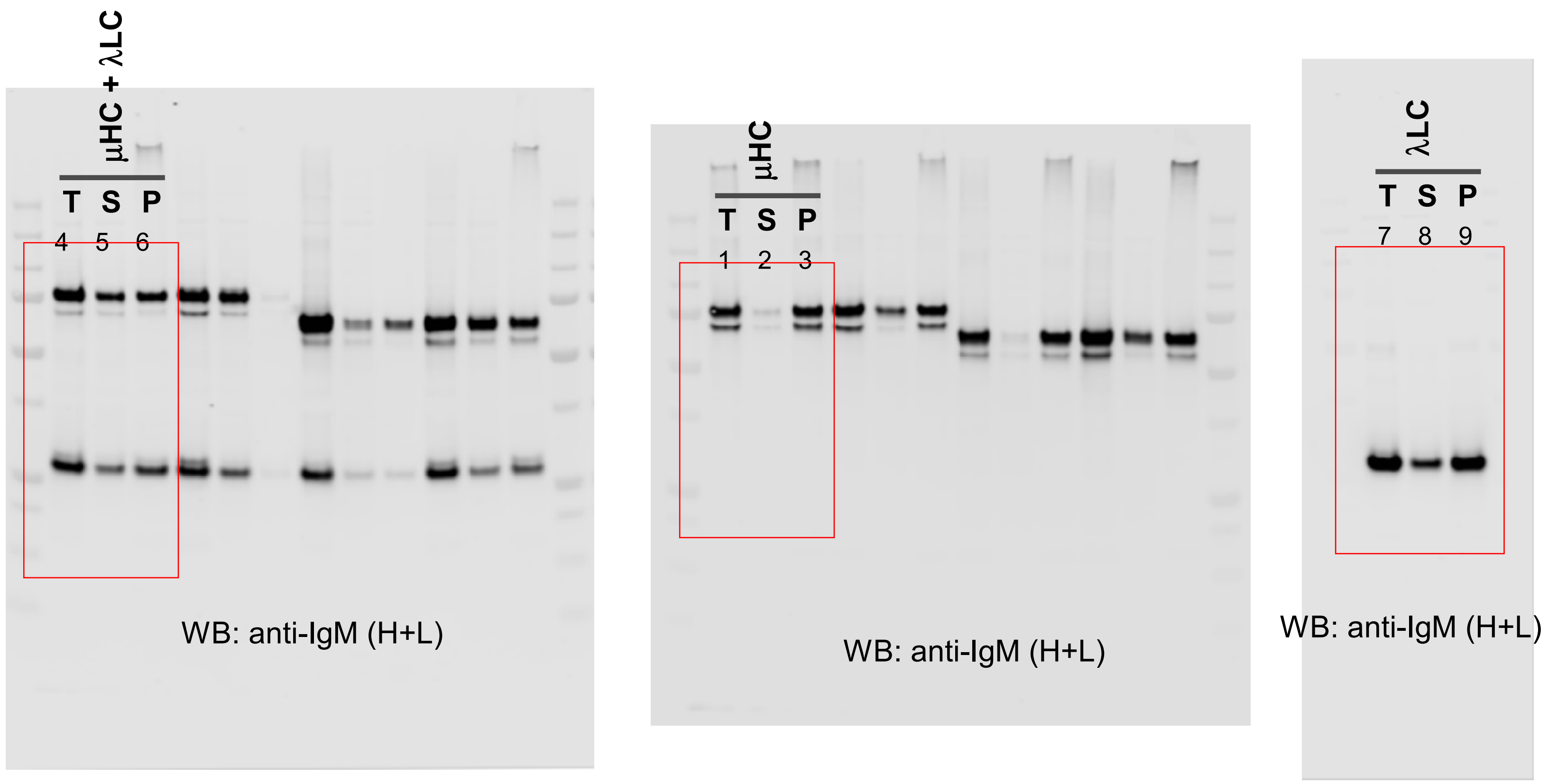

Figure 1 G: The original blots used to generate Fig. 1G are shown below.

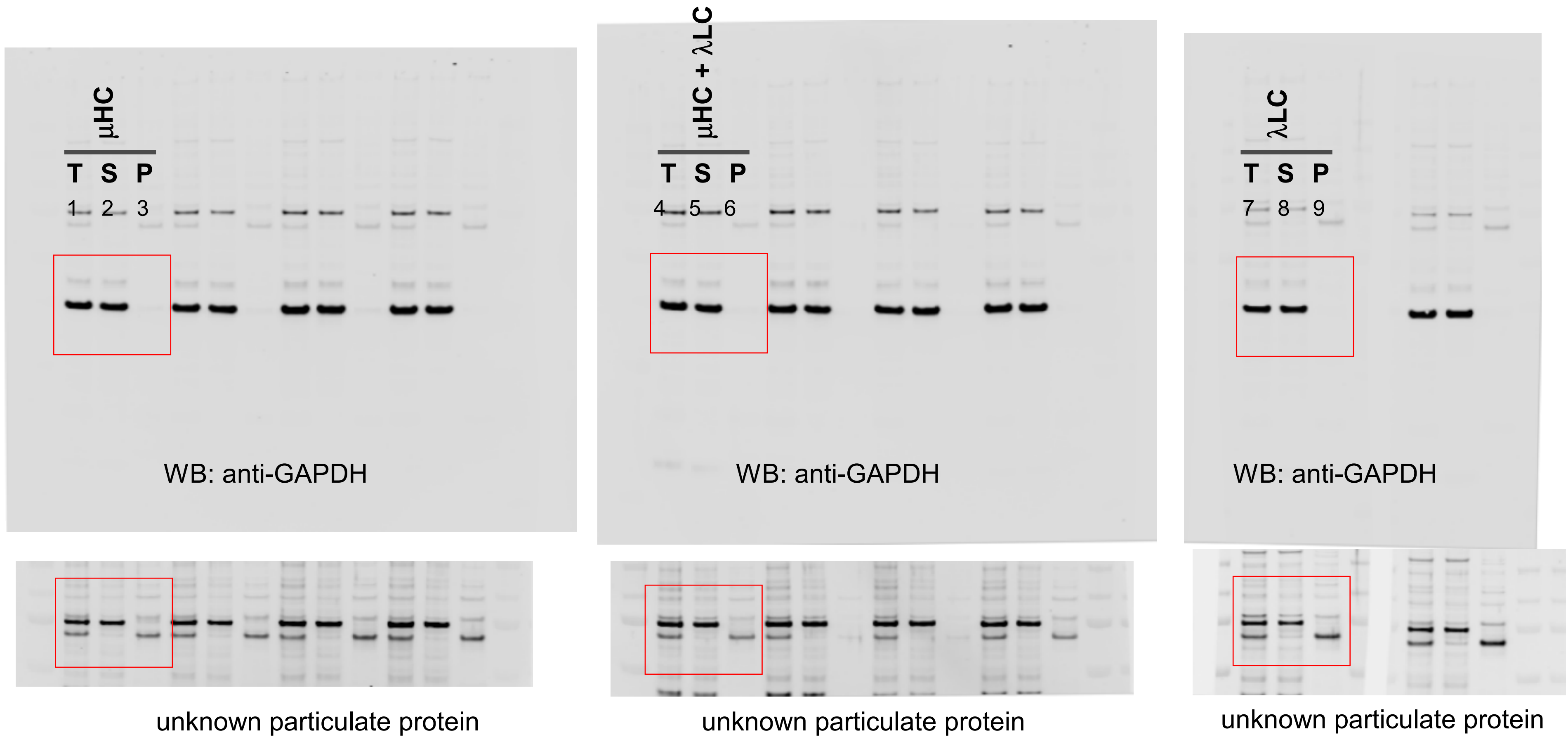

Figure 2 D E F G: The raw gels and blots before the cropping are shown below. The cropped regions are shown in red box.

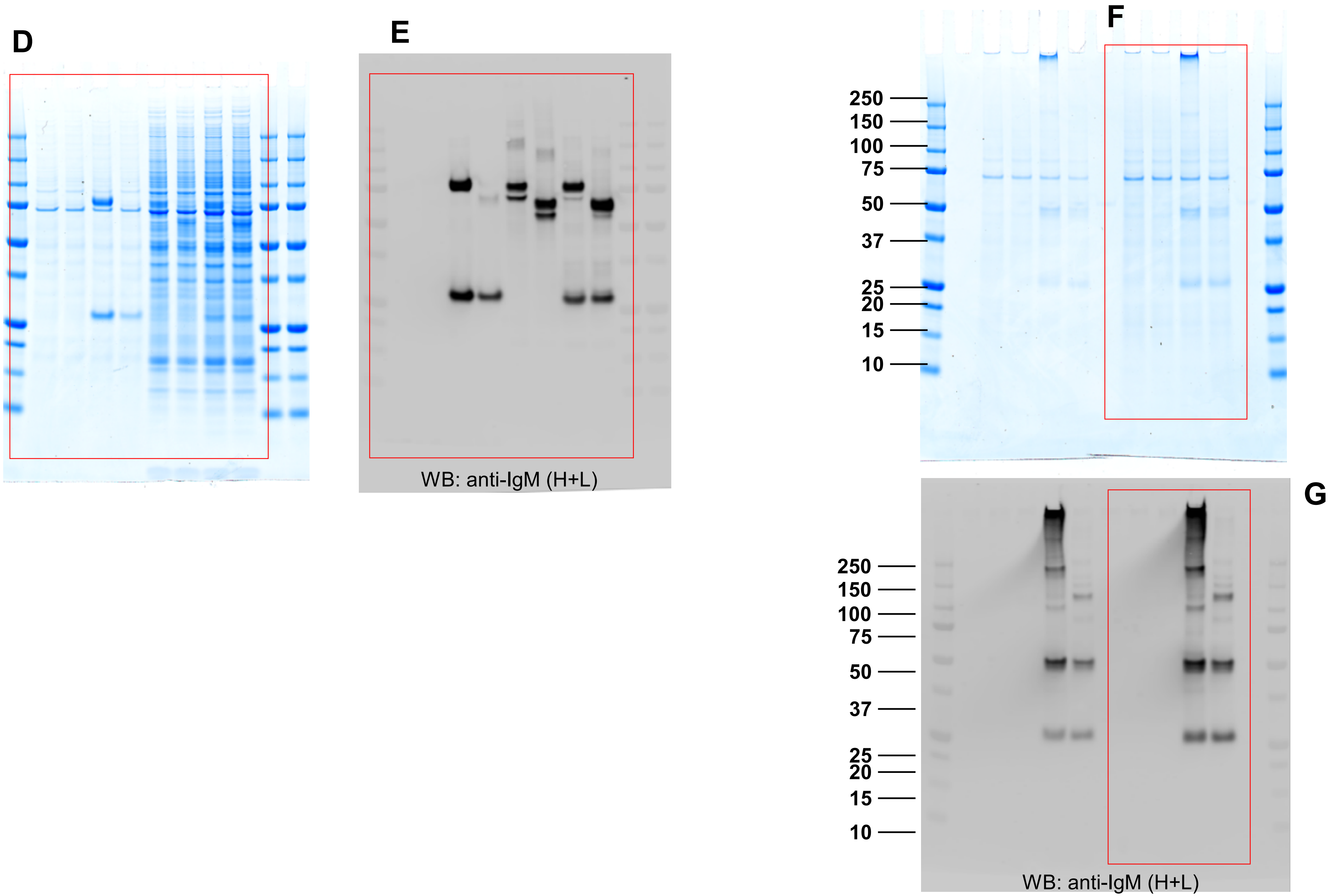

Figure 3 C D E: The raw gels and blots before the cropping are shown below. The cropped regions are shown in red box.

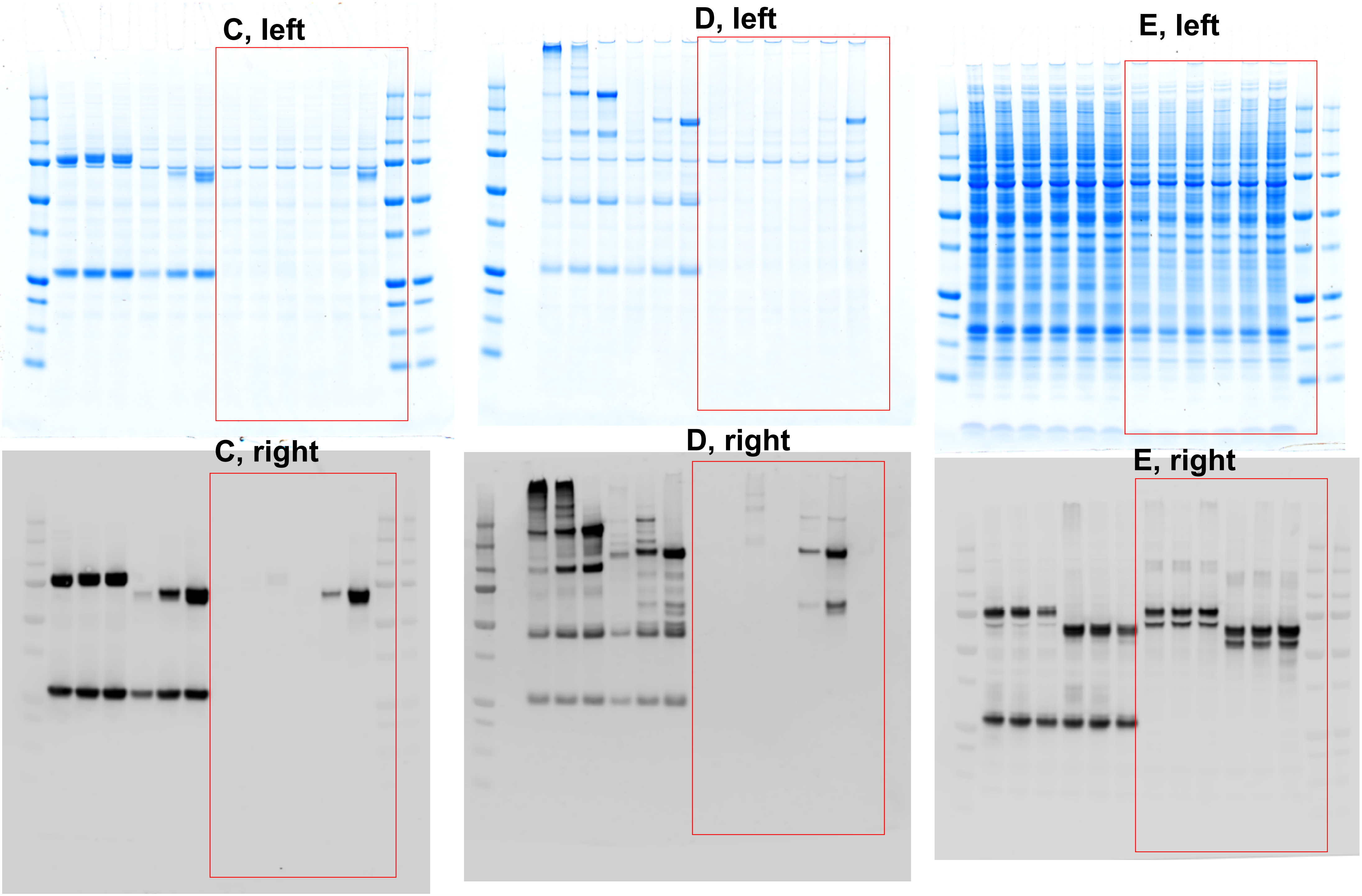

Figure 4 C D E: The raw gels and blots before the cropping are shown below. The cropped regions are shown in red box.

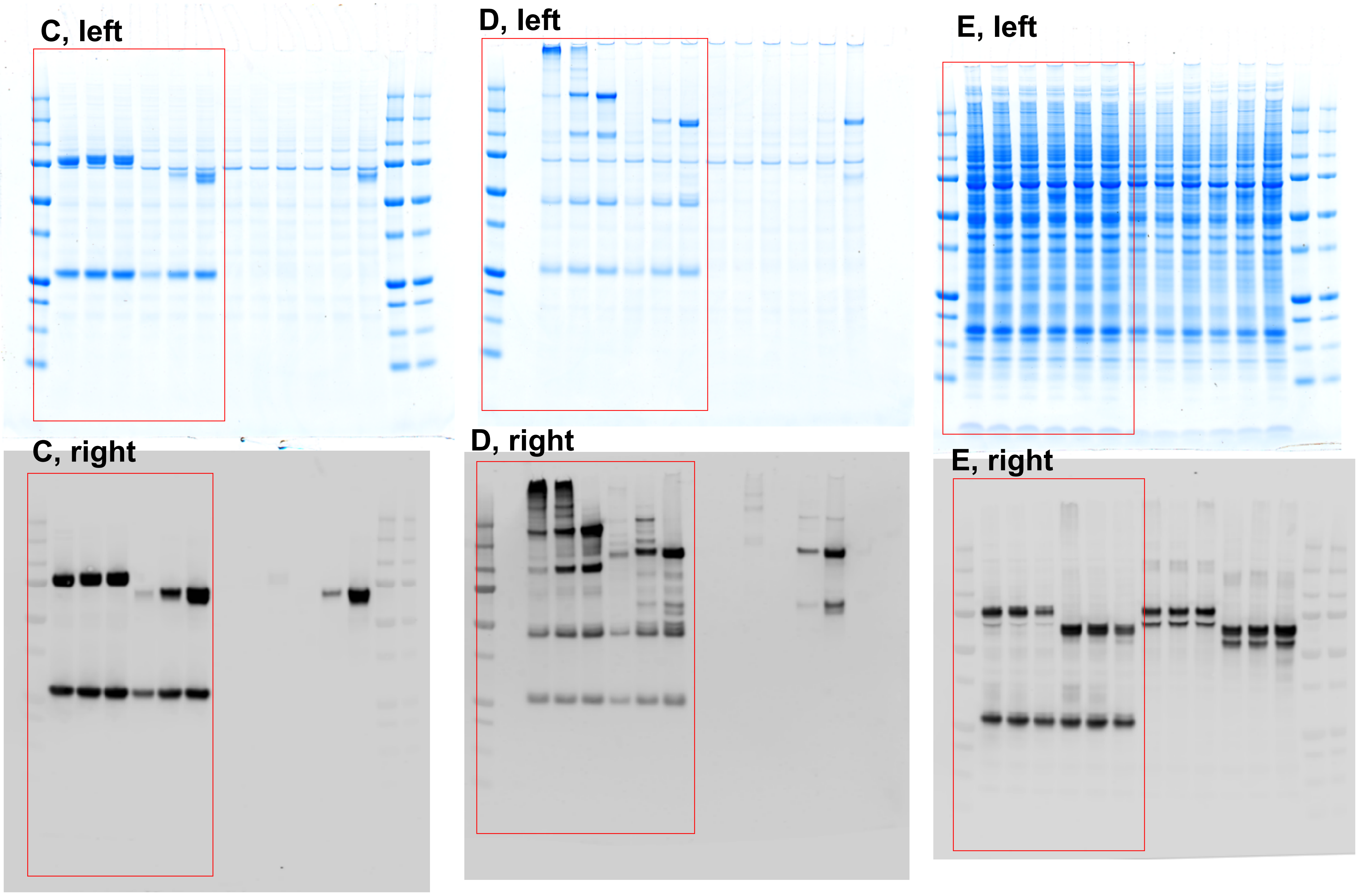

Figure 5 A, C, D, E, F, G: The raw gel and blot before the cropping are shown below. The cropped regions are shown in red box.

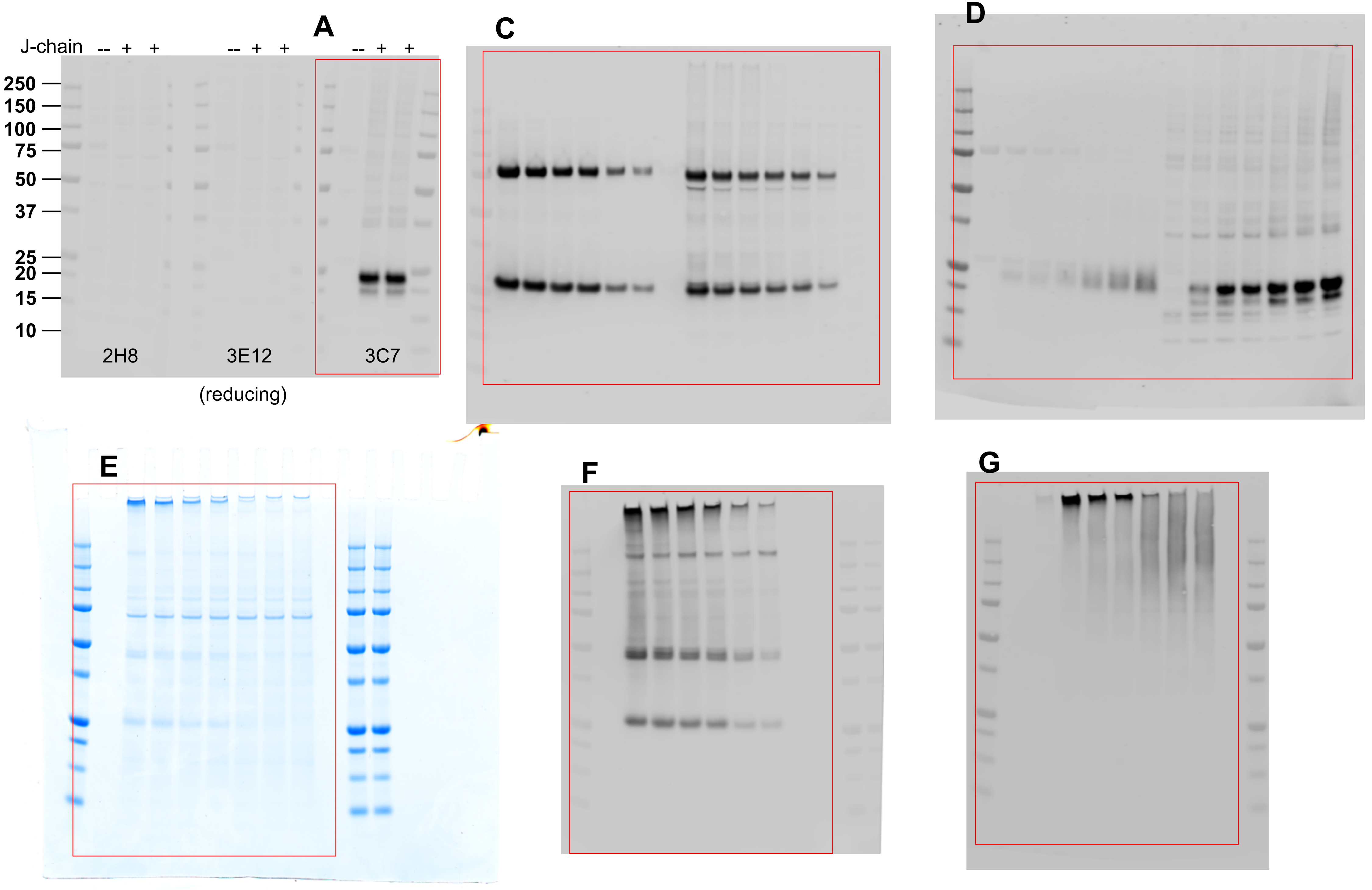

Figure 6 A: The bottom one-third of the gel and blots below 55 kDa was cut off to save space. The original gel and blots (from the loading lane to the bottom of the gel including molecular weight markers) are shown below. The cropped regions are shown in red box.

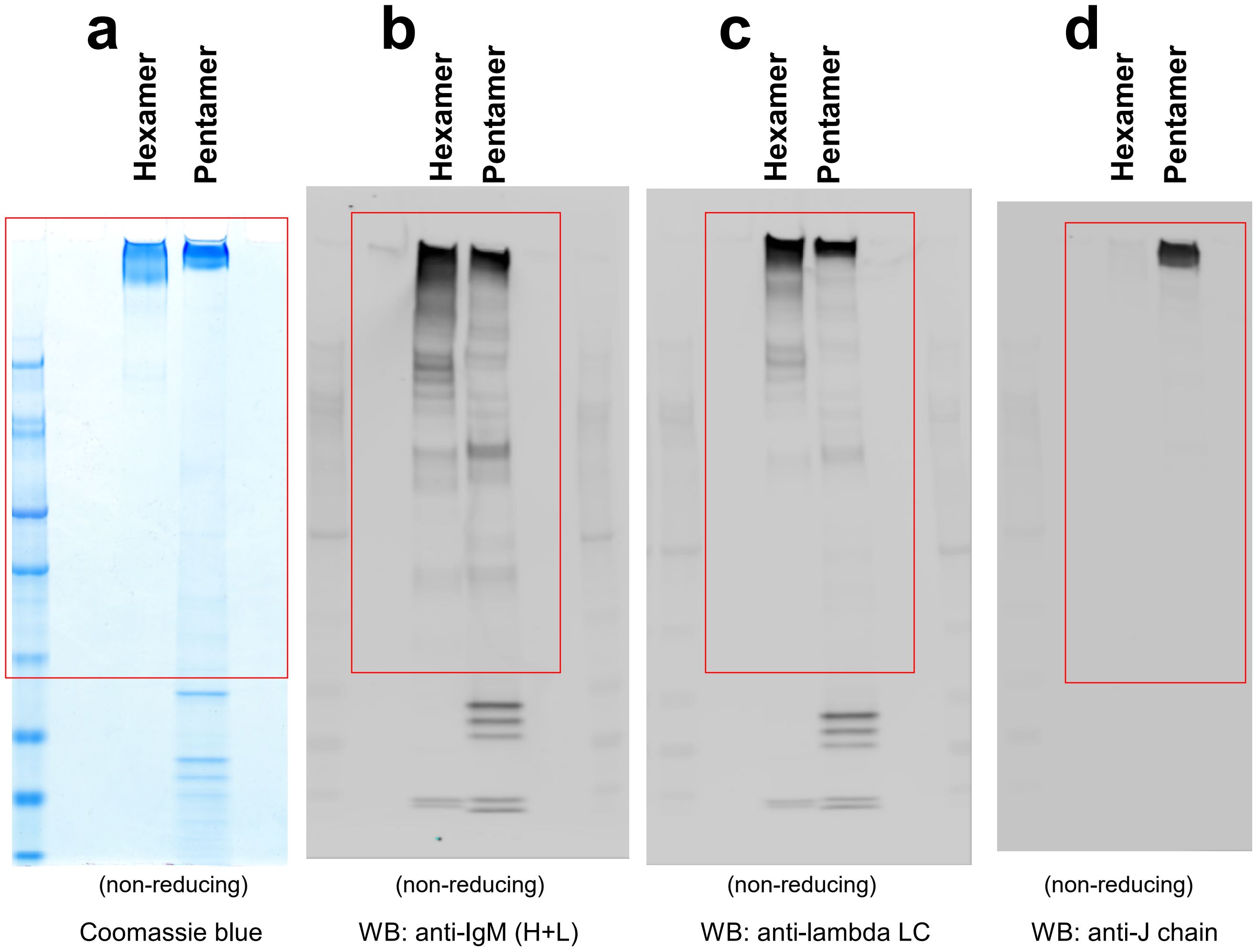

Figure 7 E: The raw blots before the cropping are shown below. The cropped regions are shown in red box.

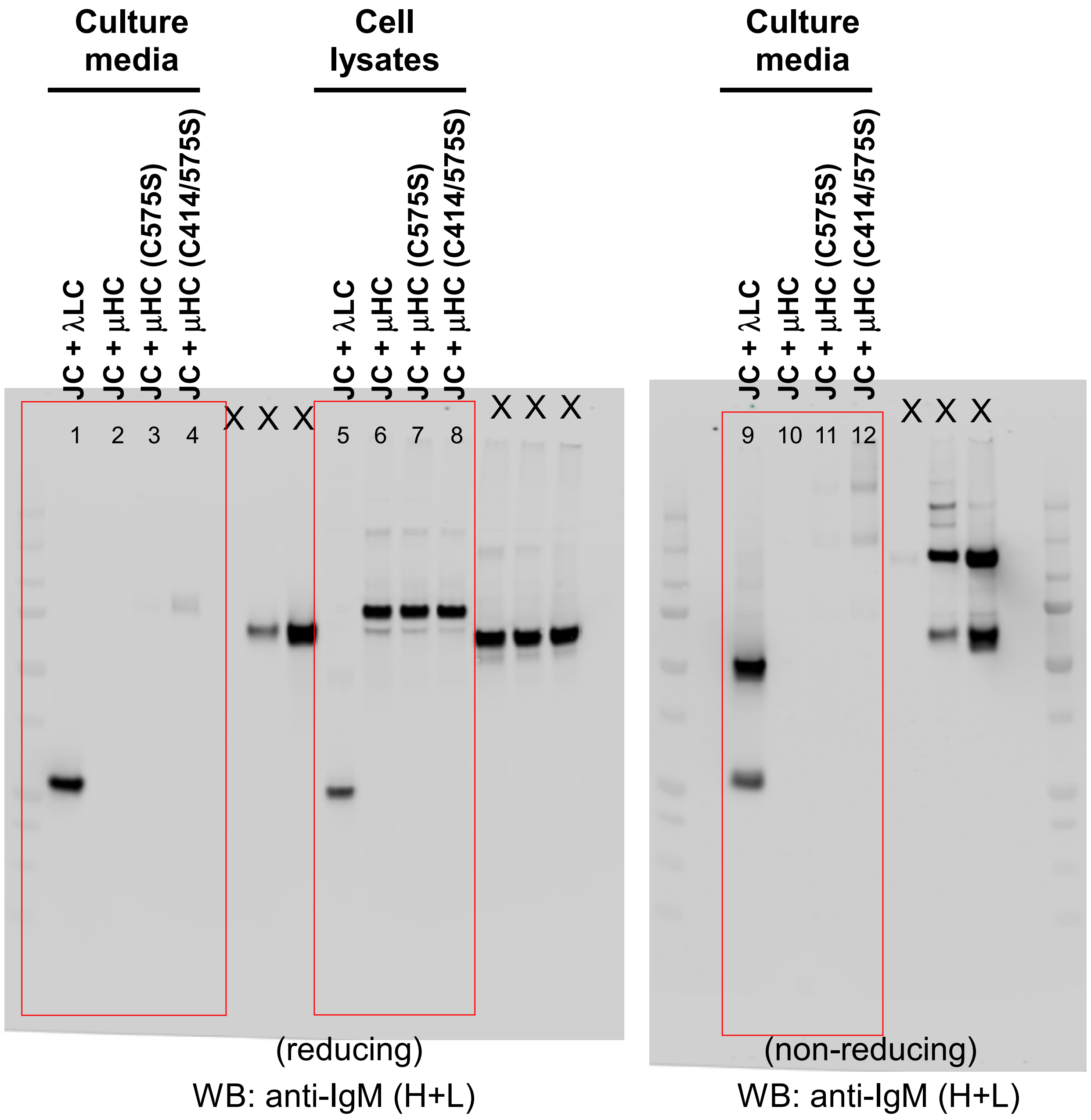

Figure 7 F: The raw blots before the cropping are shown below. The cropped regions are shown in red box.

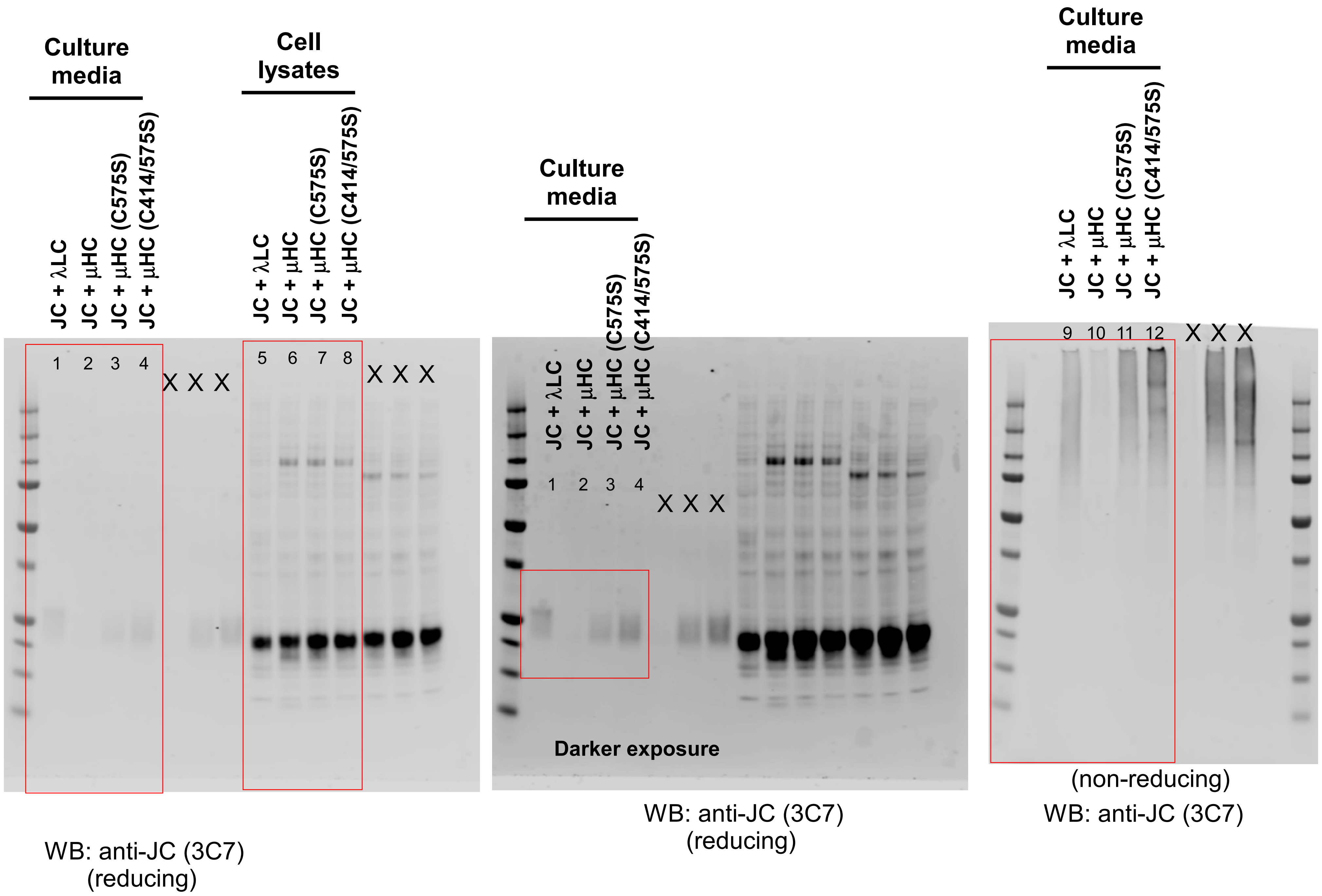

Figure 8 A: HC region and LC region were cropped from a same blot. The JC region was cropped from a different blot. The raw blots before the cropping are shown below. The cropped regions are shown in red box.

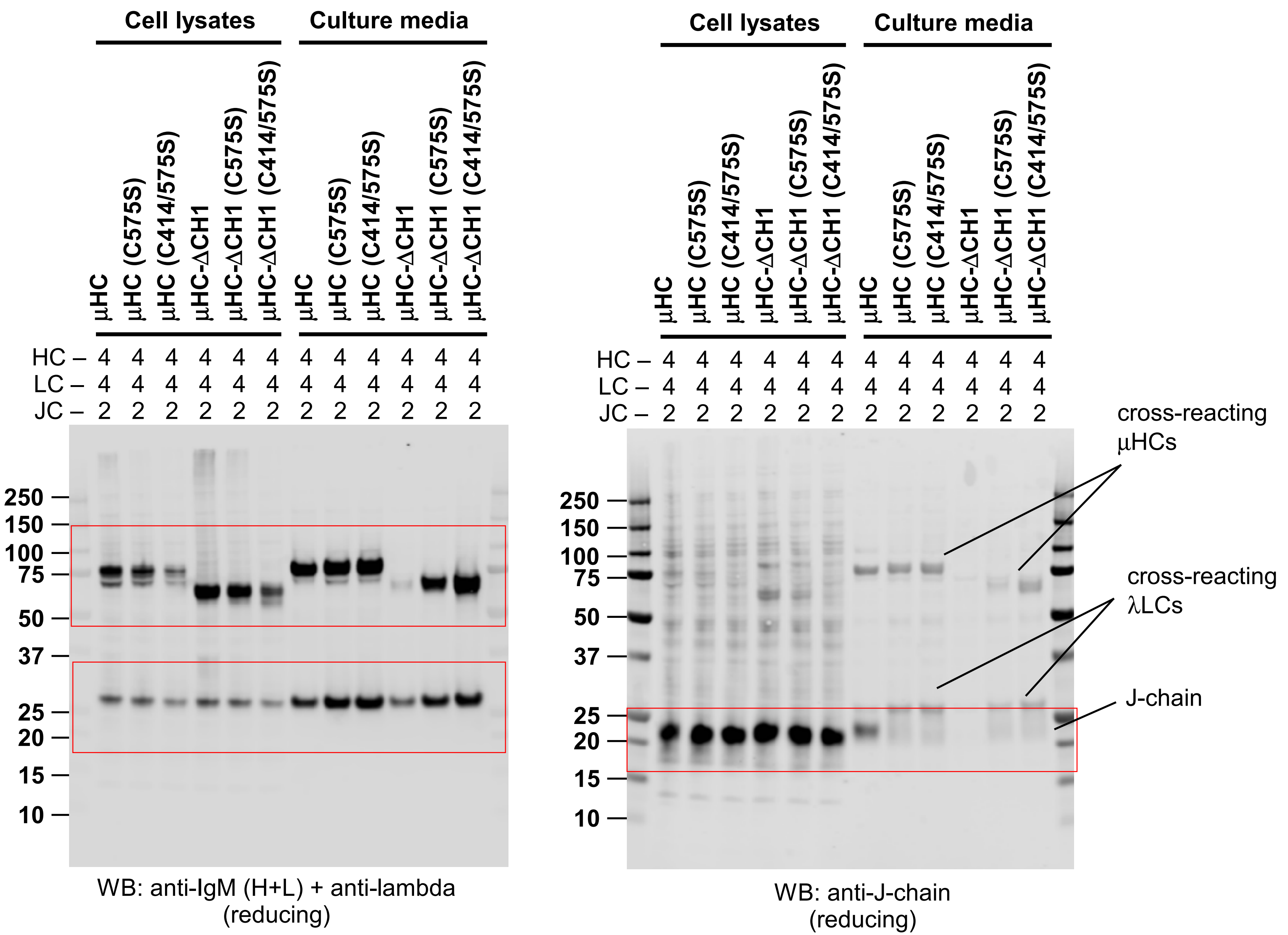

Figure 8 B C D: The raw gel and blot before the cropping are shown right. The cropped regions are shown in red box.

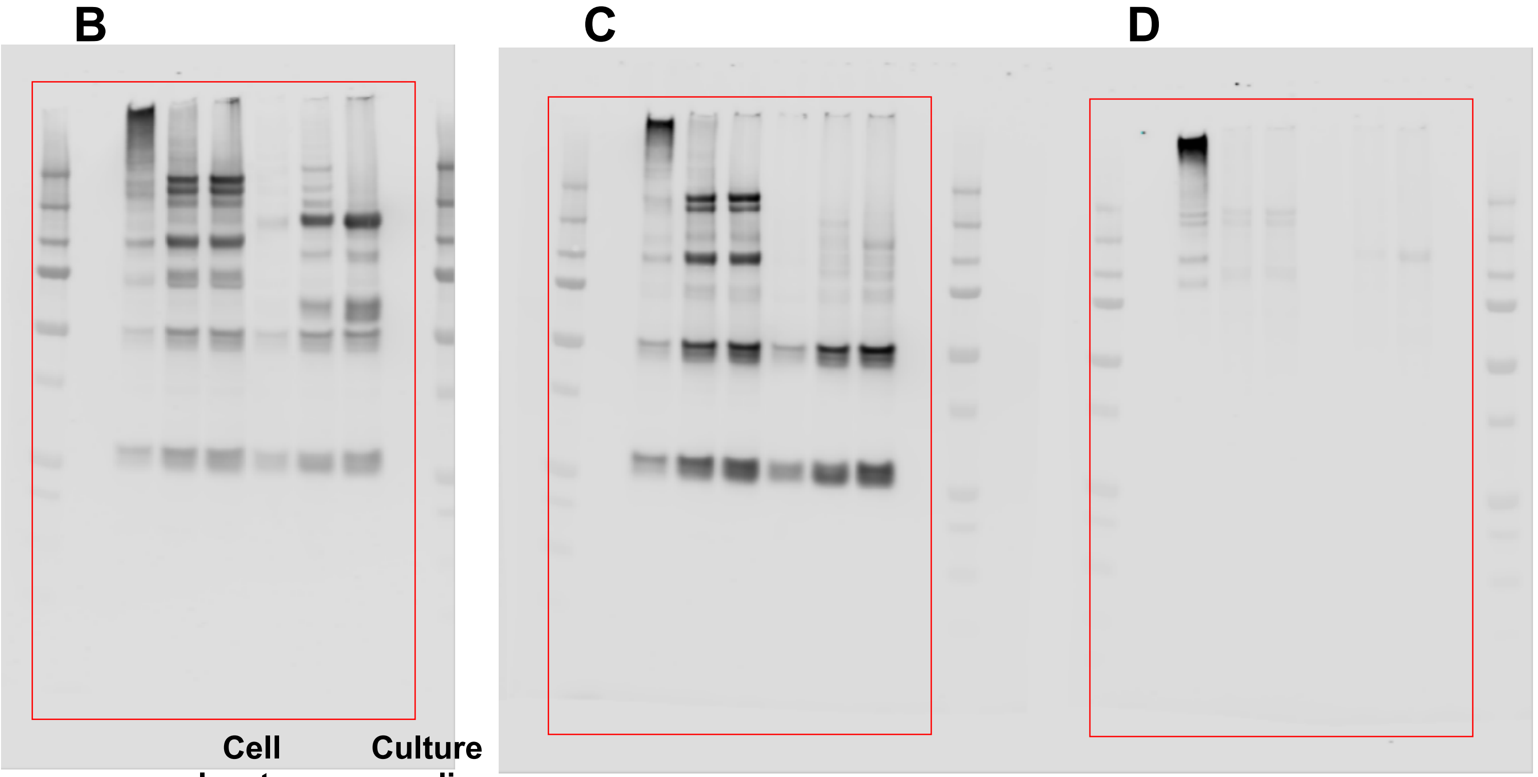

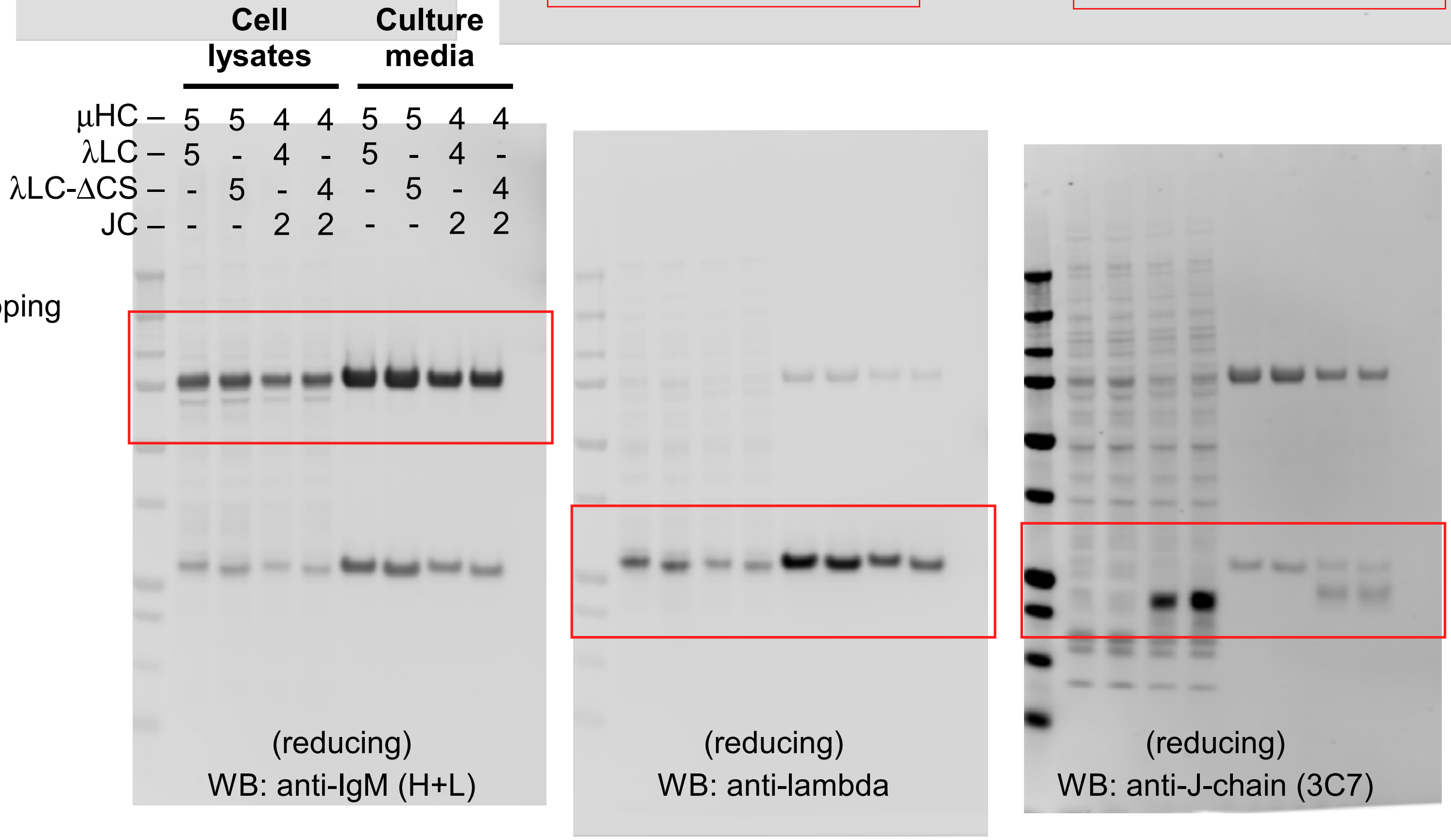

Figure 9 A: The raw blots before the cropping are shown right. The cropped regions are shown in red box.

Figure 9 B C D: The raw gel and blot before the cropping are shown below. The cropped regions are shown in red box.

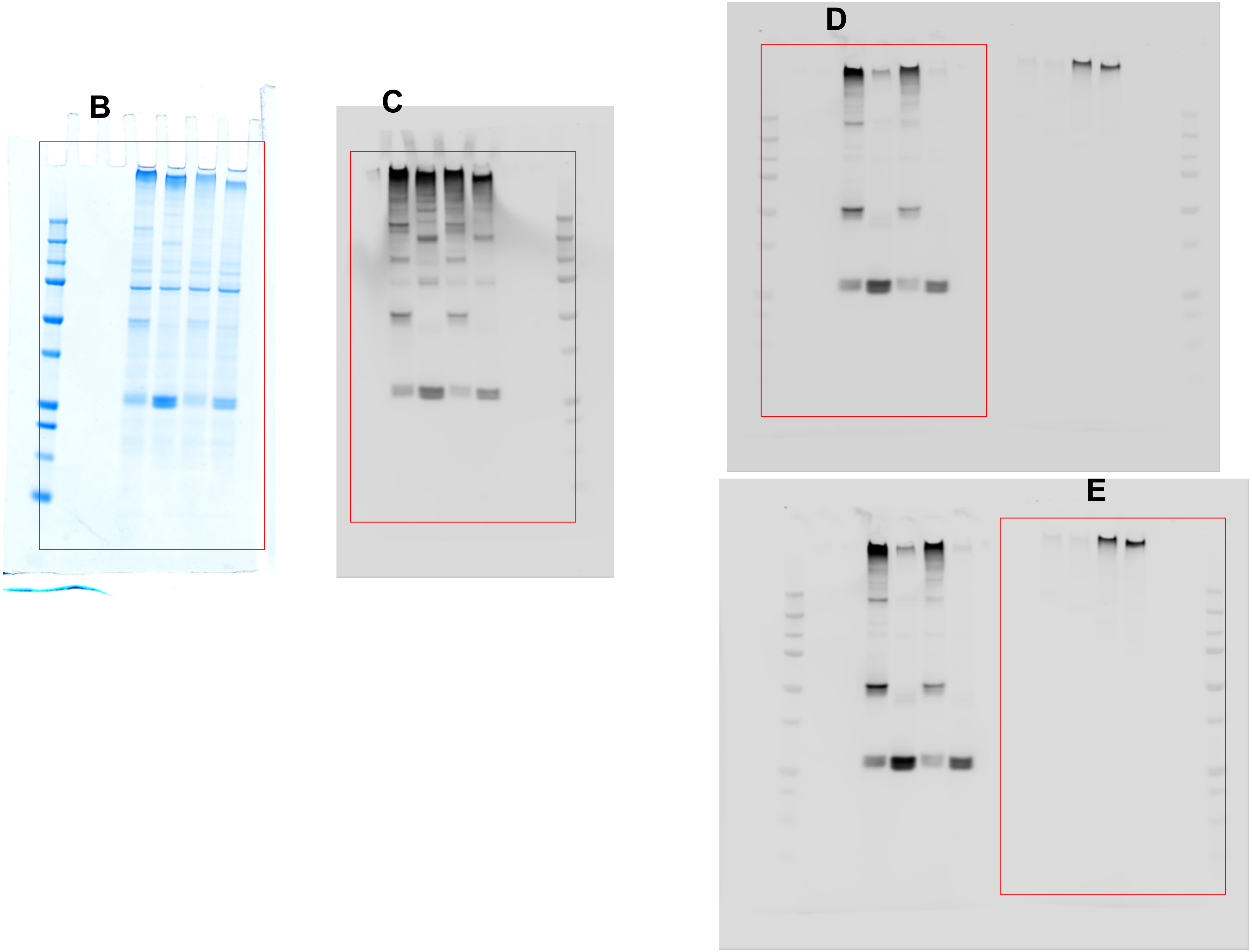

Figure 10 A B C: The raw gel and blot before the cropping are shown below. The cropped regions are shown in red box.

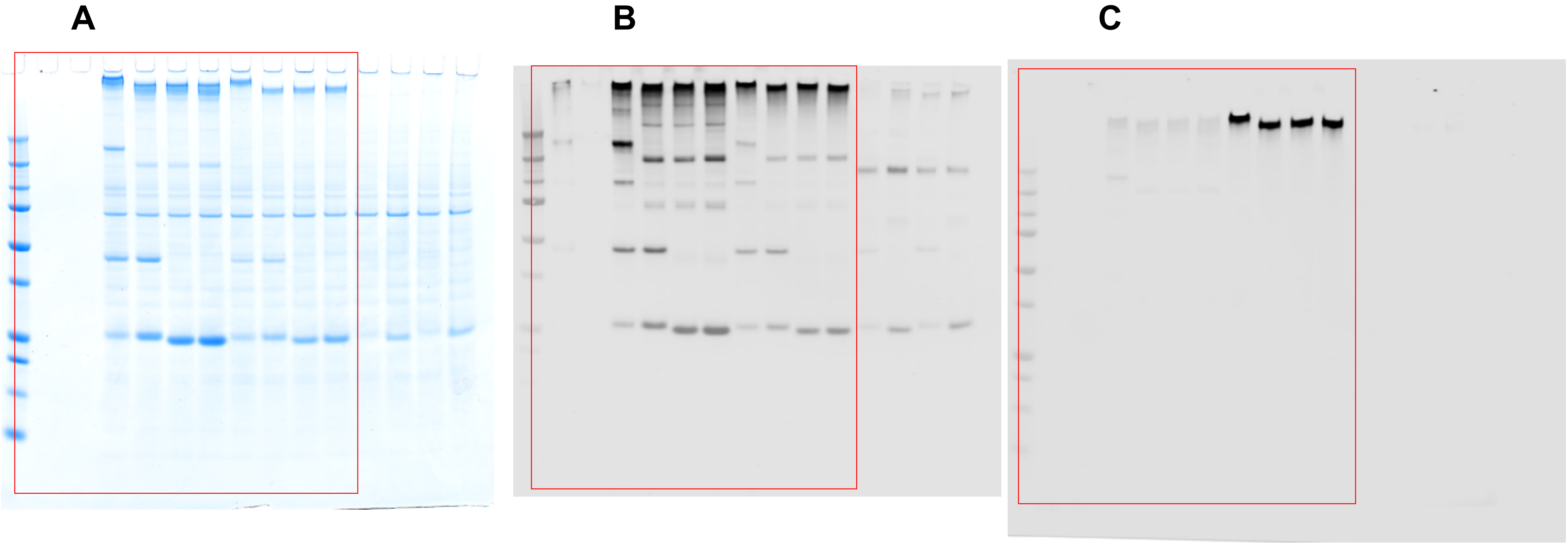

Figure 11 A B C D: The raw gel and blot before the cropping are shown below. The cropped regions are shown in red box.

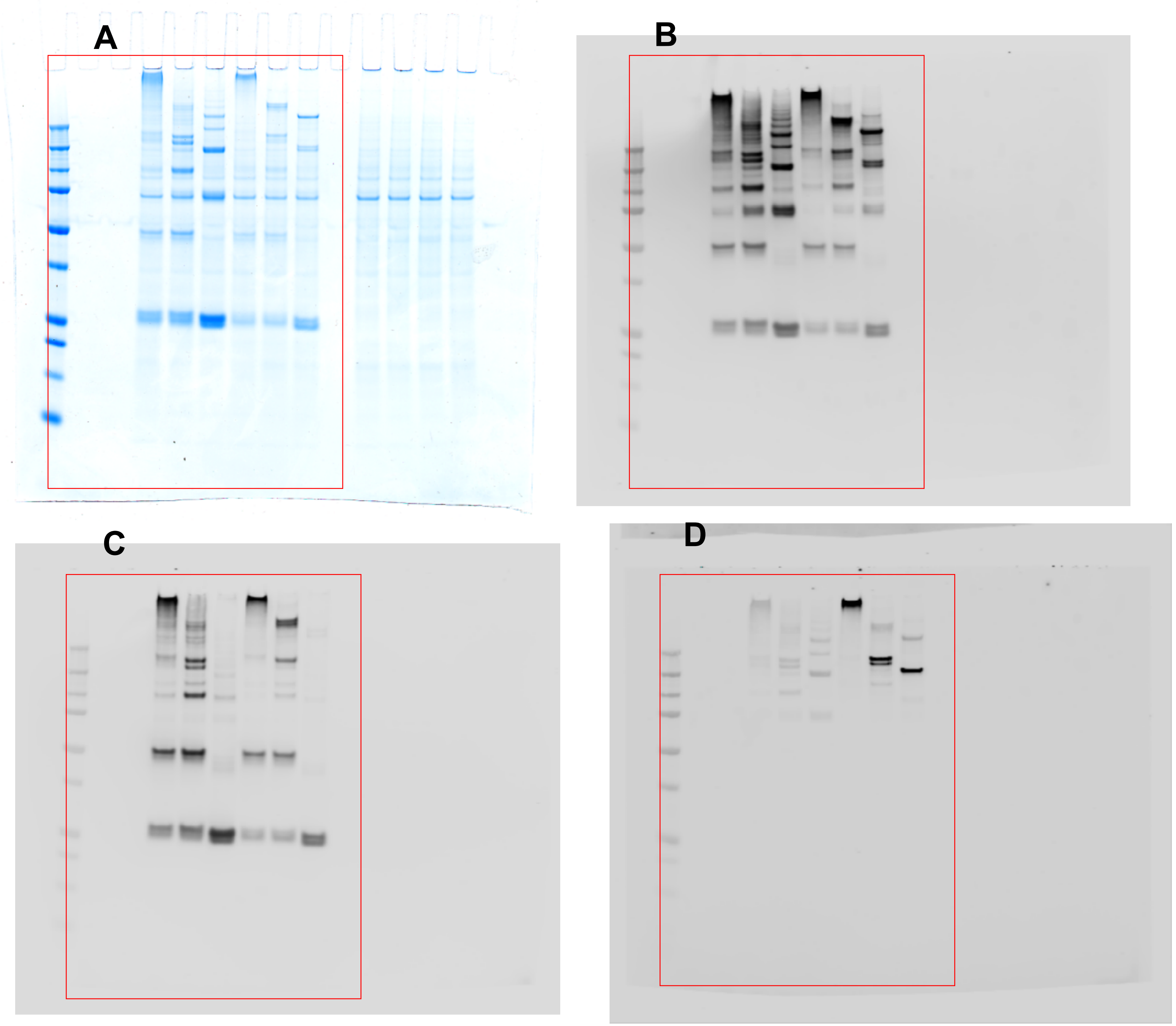

Figure 12 A B C D E F G: The raw gel and blot before the cropping are shown below. The cropped regions are shown in red box.

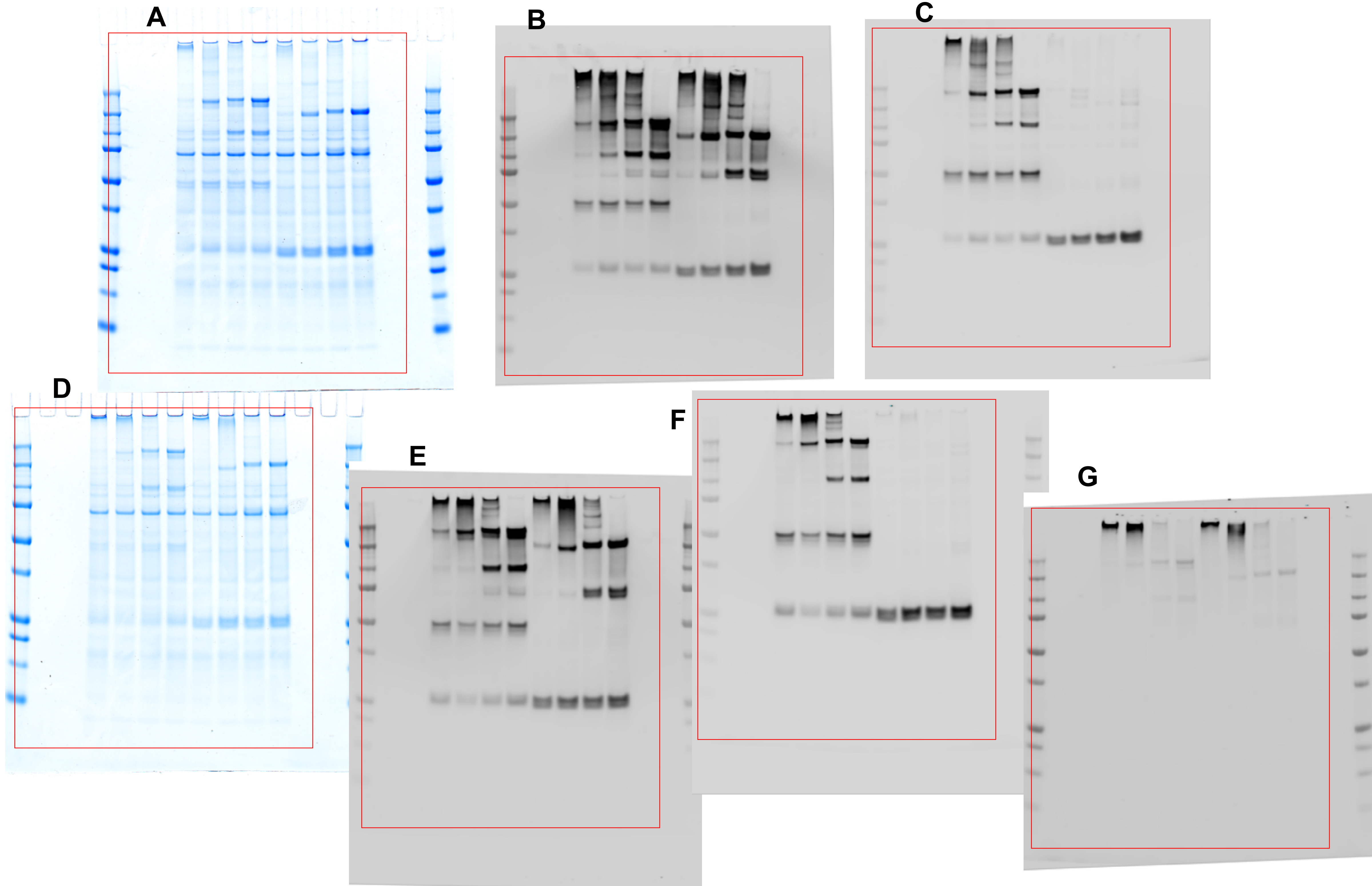

Figure 13 A B C D: The raw gel and blot before the cropping are shown below. The cropped regions are shown in red box.

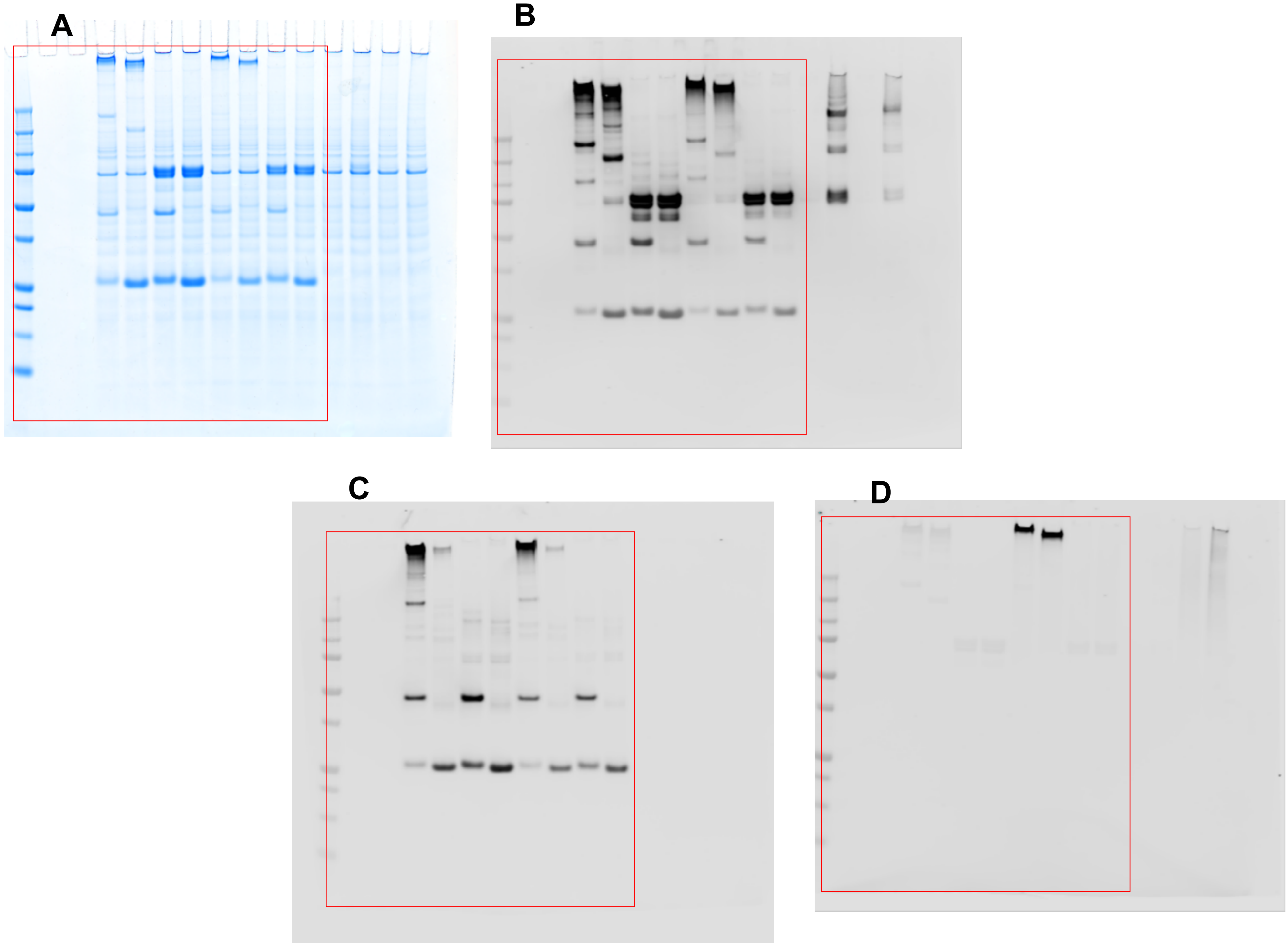

S4 Fig B C: The raw blots before the cropping are shown below. The cropped regions are shown in red box.

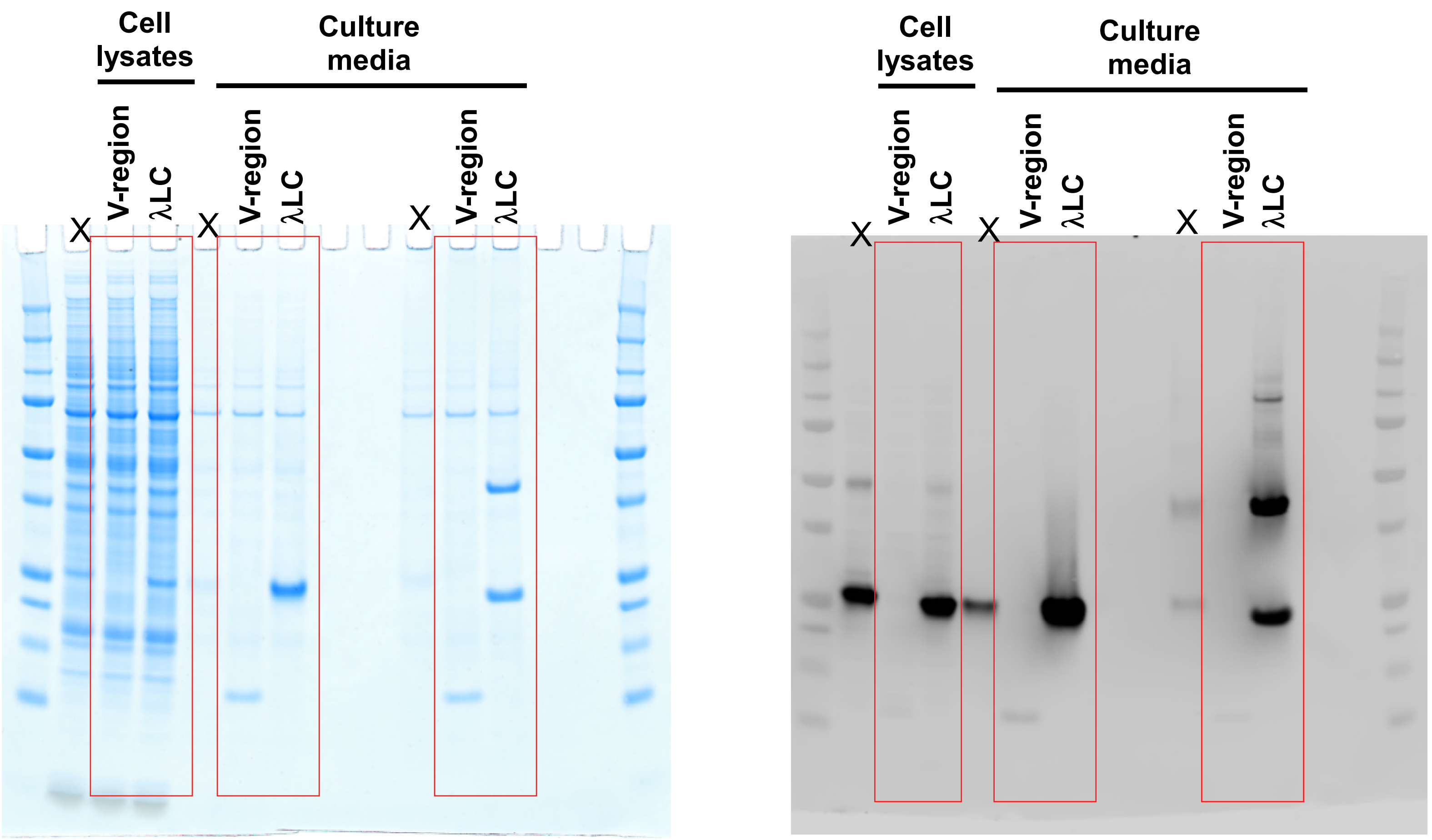

S5 Fig A: The raw blots before the cropping are shown below. The cropped regions are shown in red box.

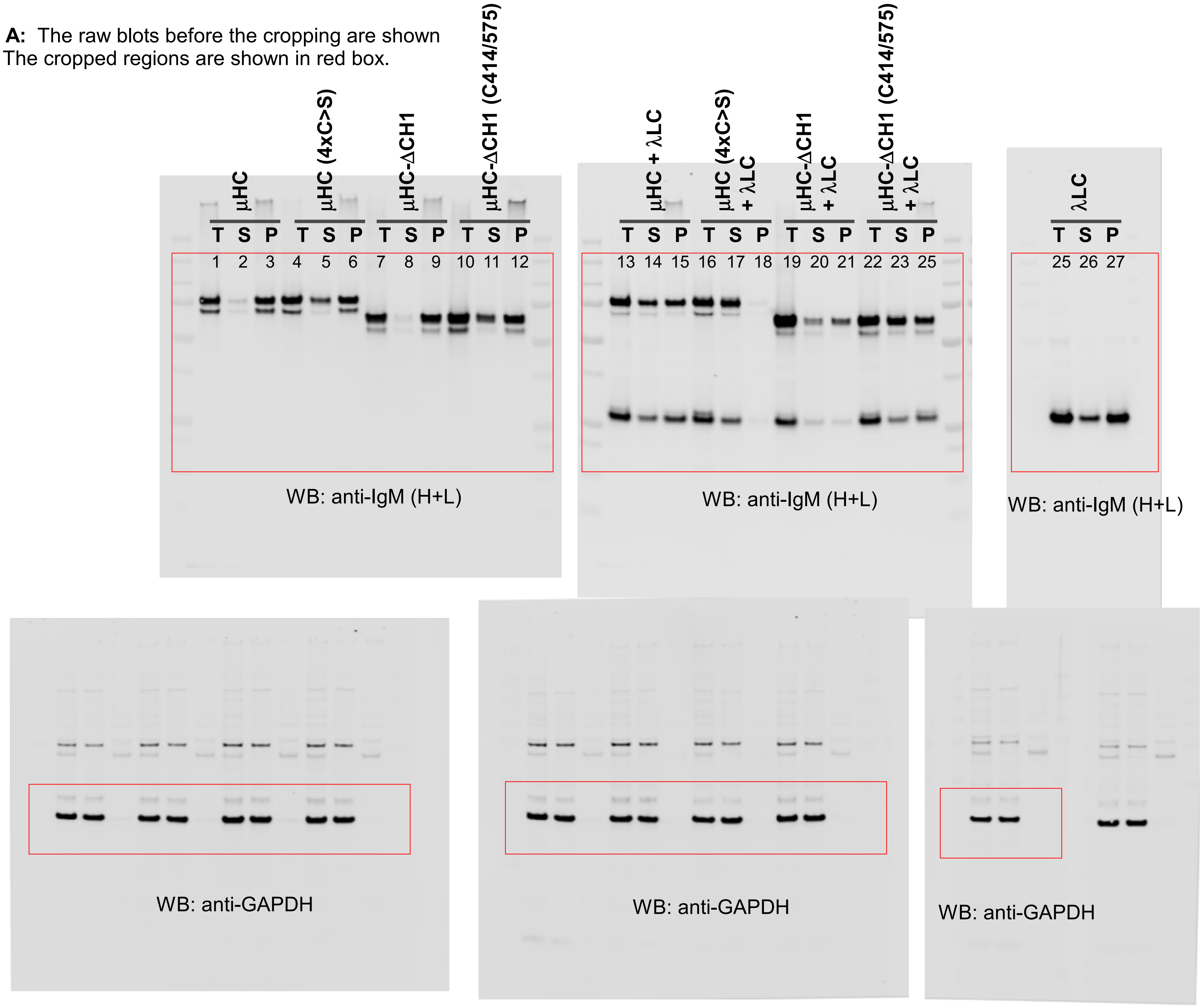

S5 Fig B top panel: The raw blots before the cropping are shown below. The cropped regions are shown in red box.

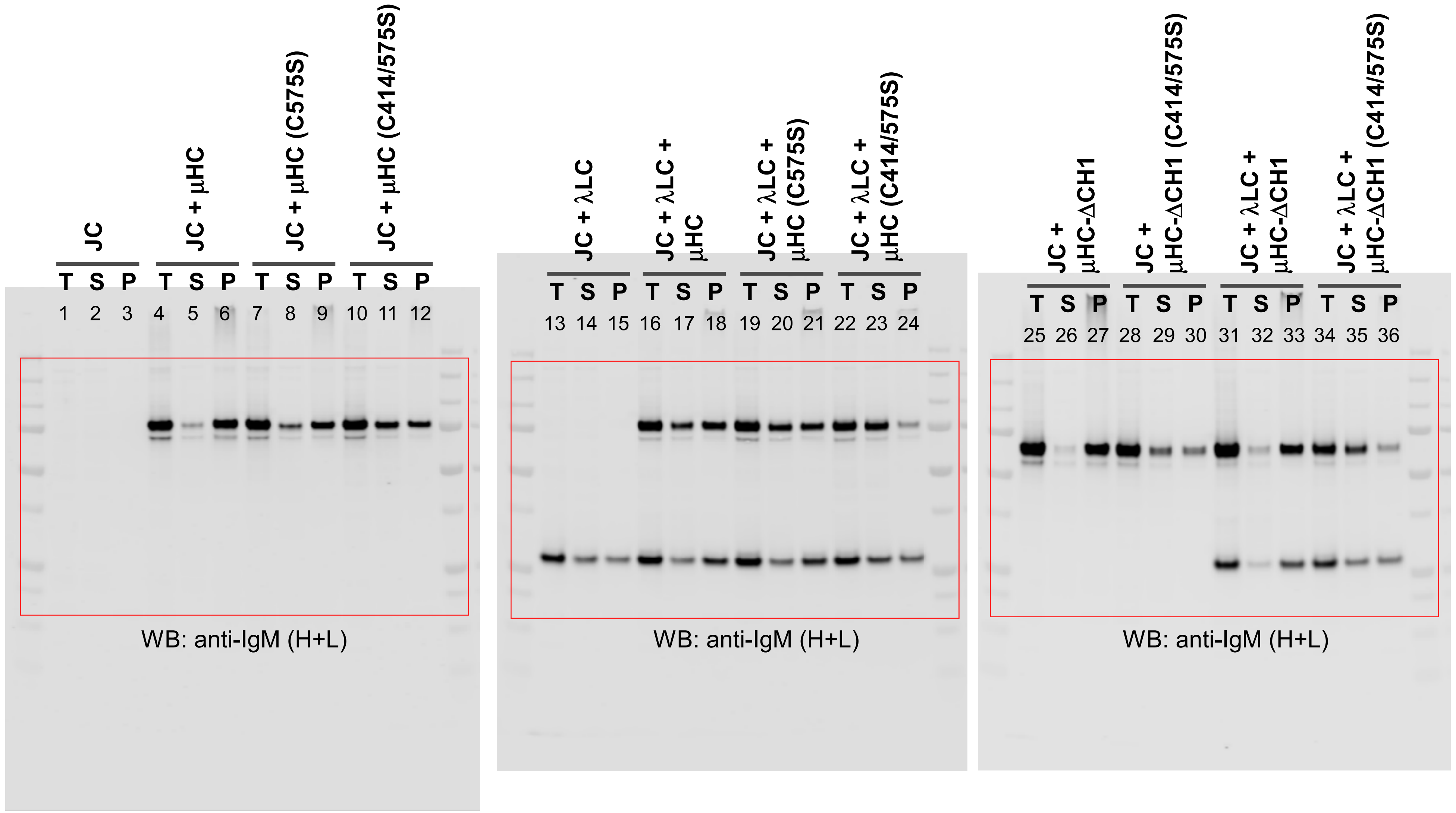

S5 Fig B middle and bottom panels: The raw blots before the cropping are shown below. The cropped regions are shown in red box.

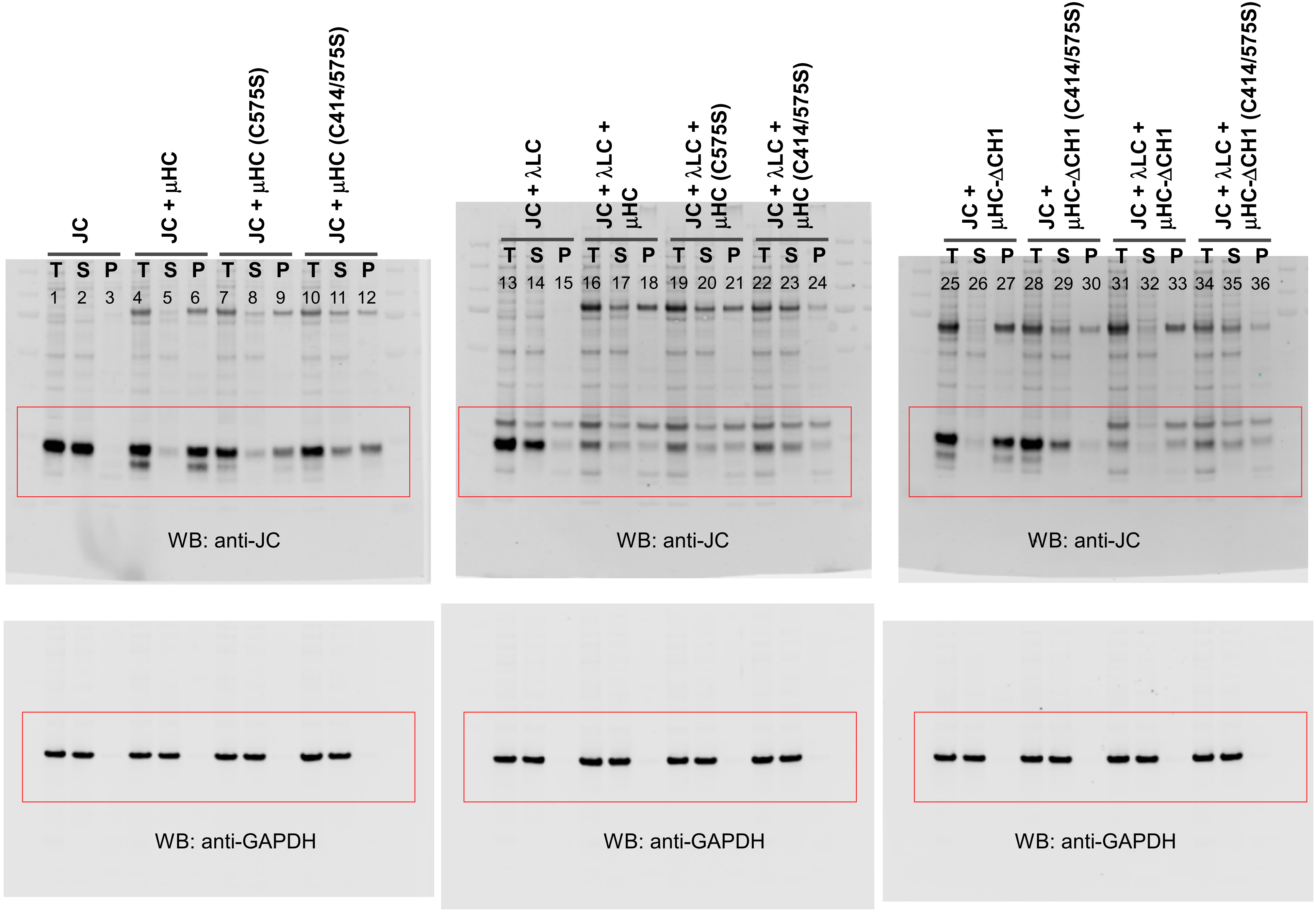

S6 Fig D: The raw blots before the cropping are shown below. The cropped regions are shown in red box.

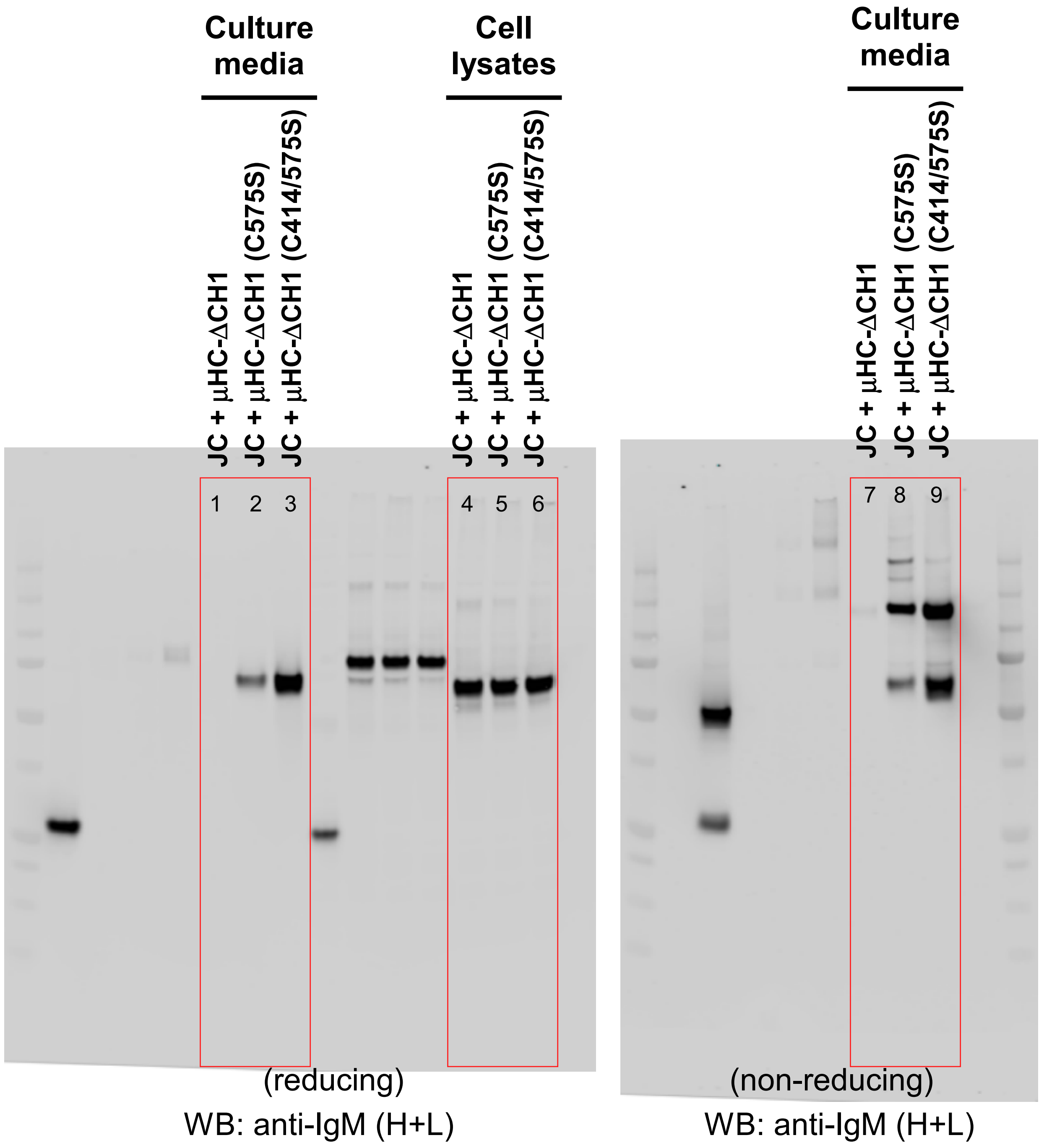

S6 Fig E: The raw blots before the cropping are shown below. The cropped regions are shown in red box.

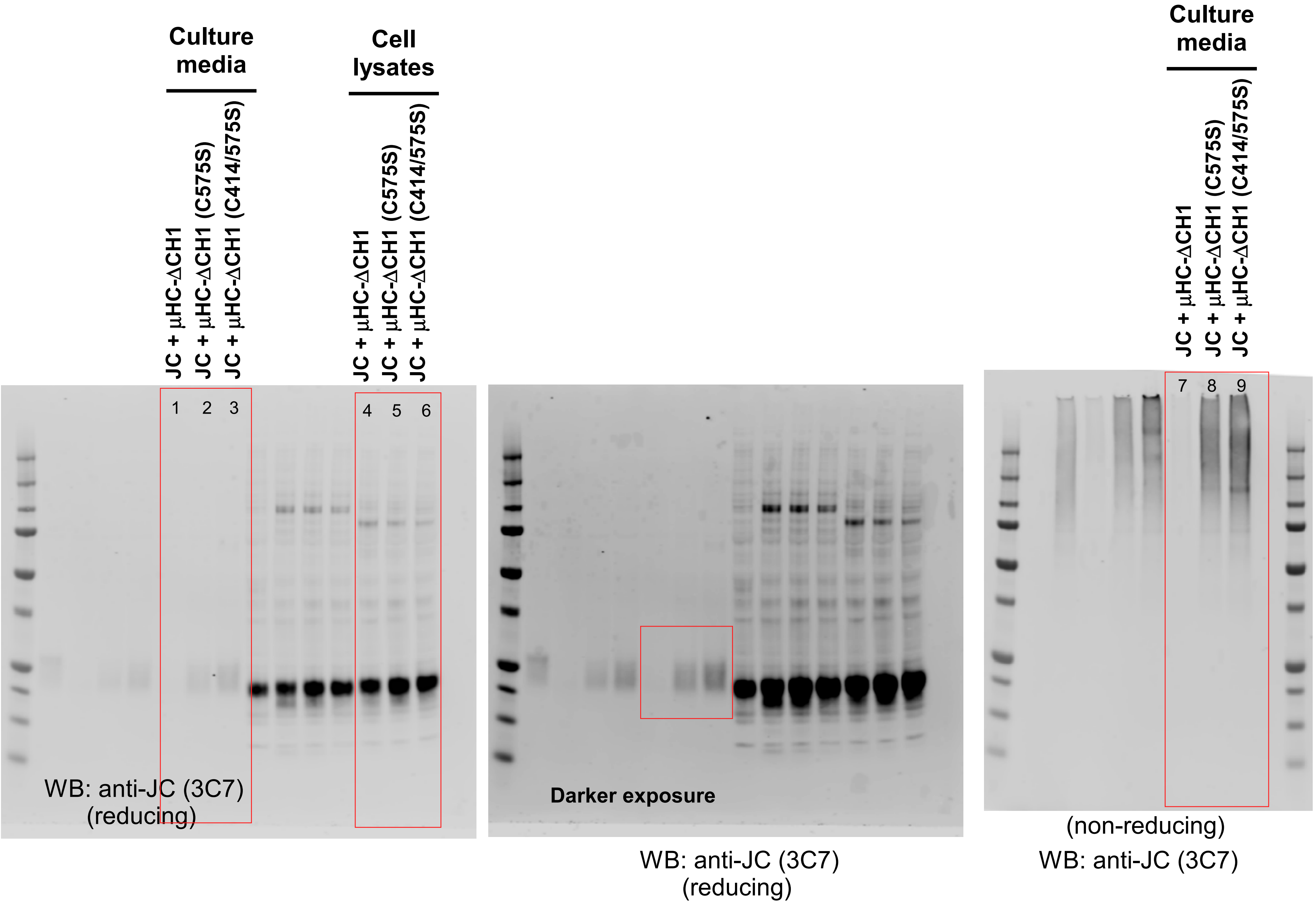

S7 Fig B C: The raw gel and blot before the cropping are shown below. The cropped regions are shown in red box.

B

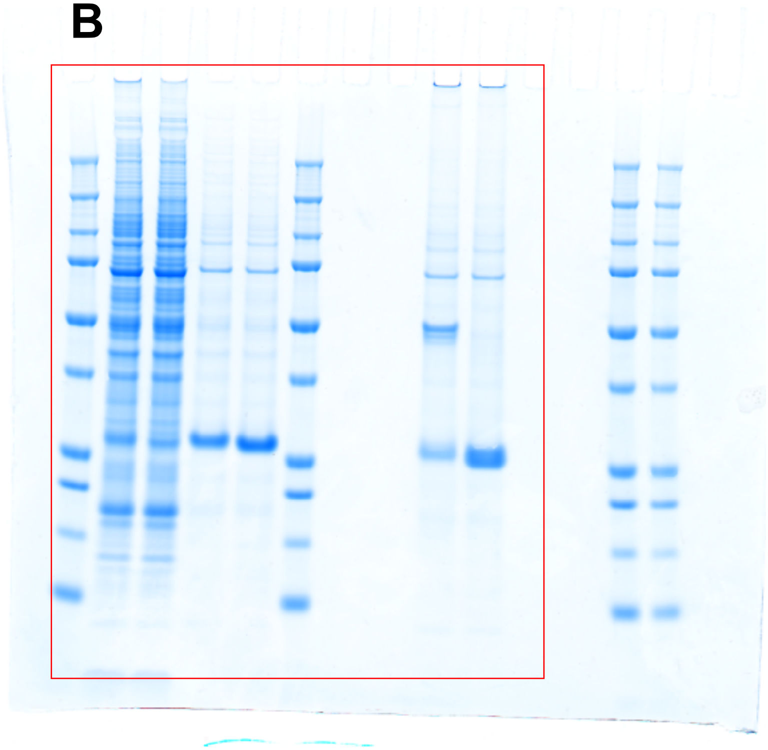

C

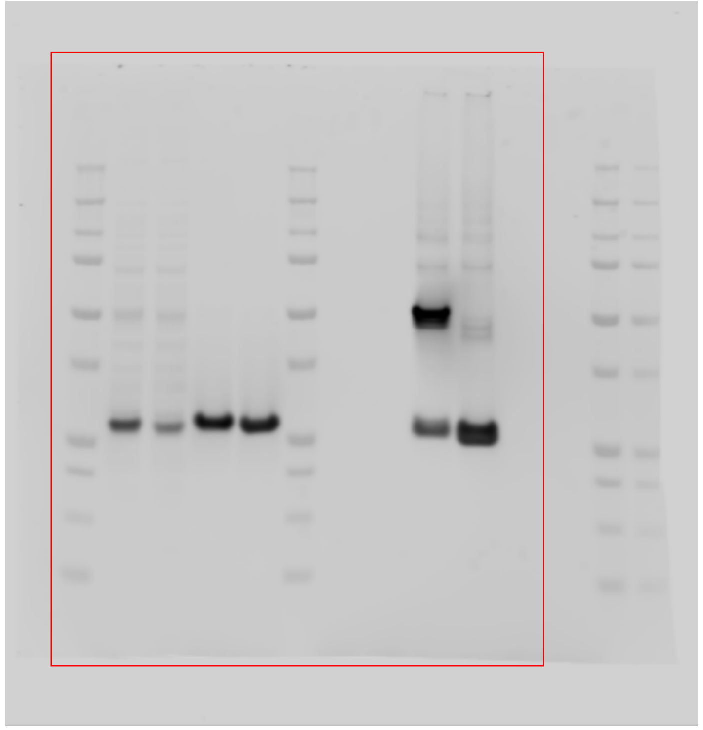

S8 Fig B C: The raw blots before the cropping are shown below. The cropped regions are shown in red box.

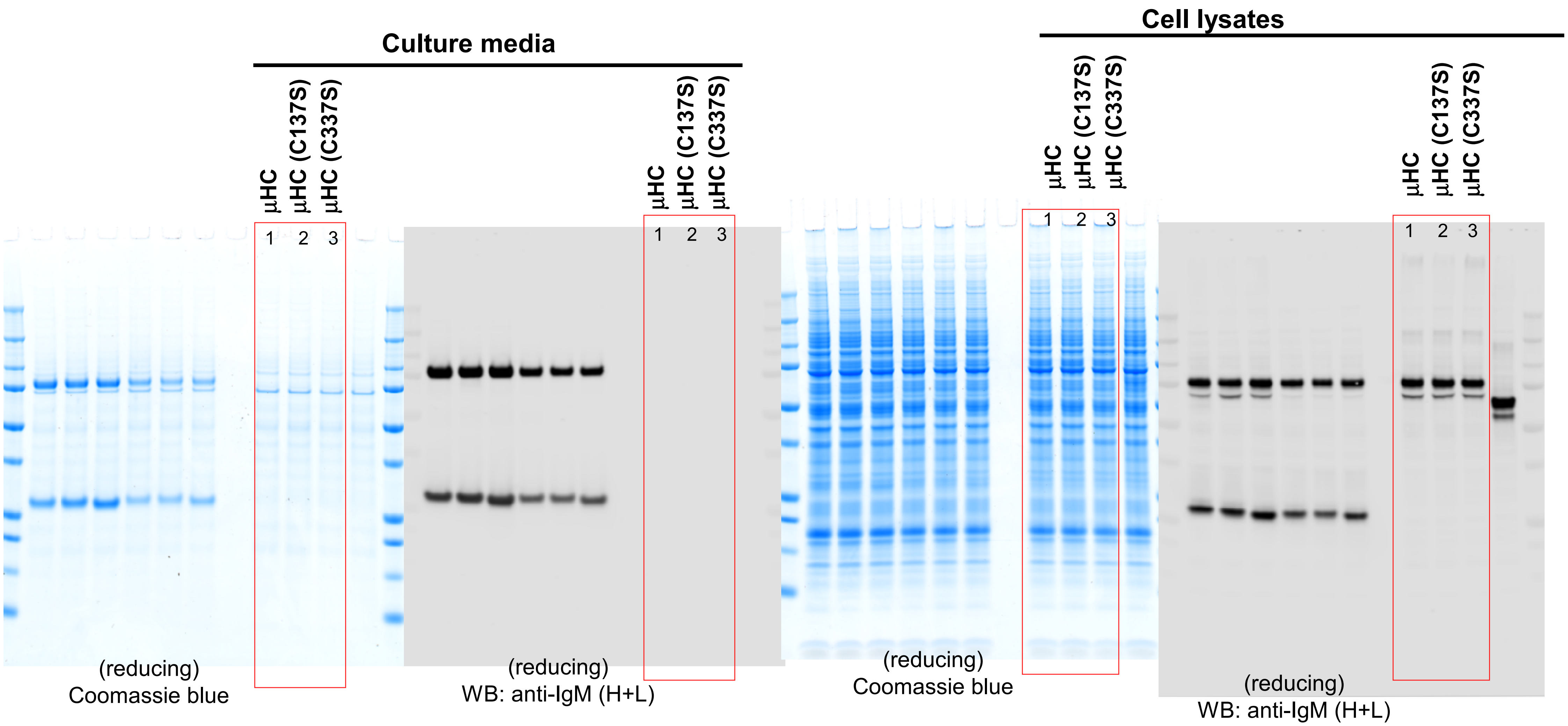

S9 Fig A B C: The raw gel and blot before the cropping are shown below. The cropped regions are shown in red box.

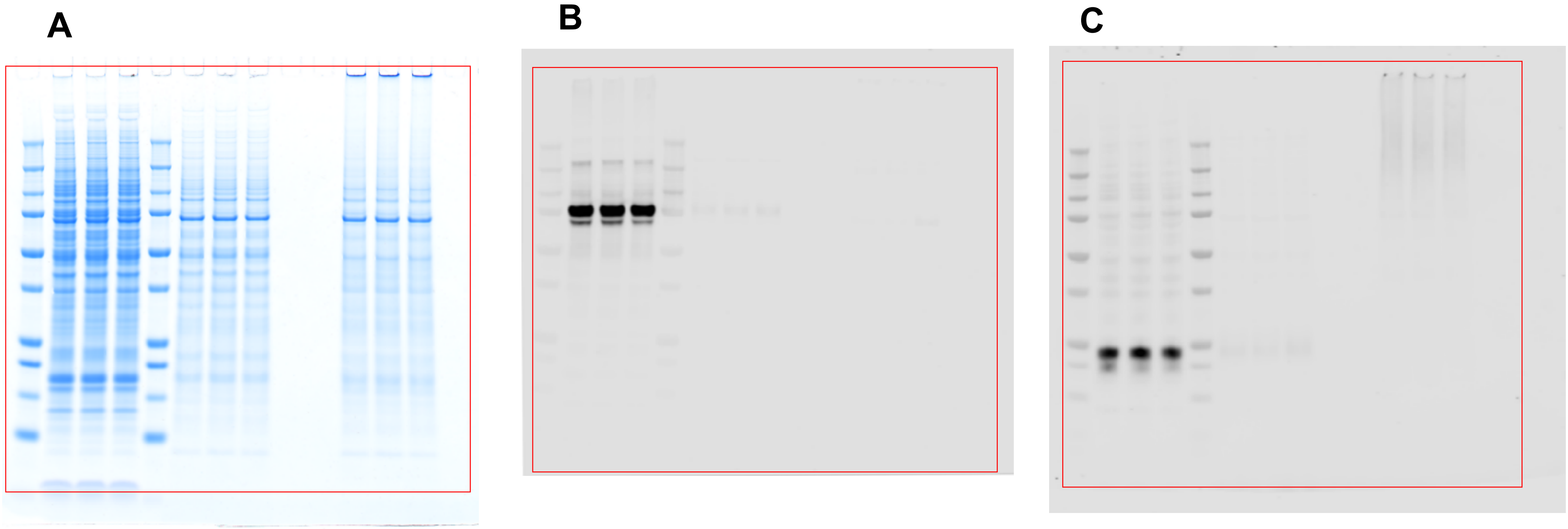

S10 Fig A B C: The raw blots before the cropping are shown below. The cropped regions are shown in red box.

A

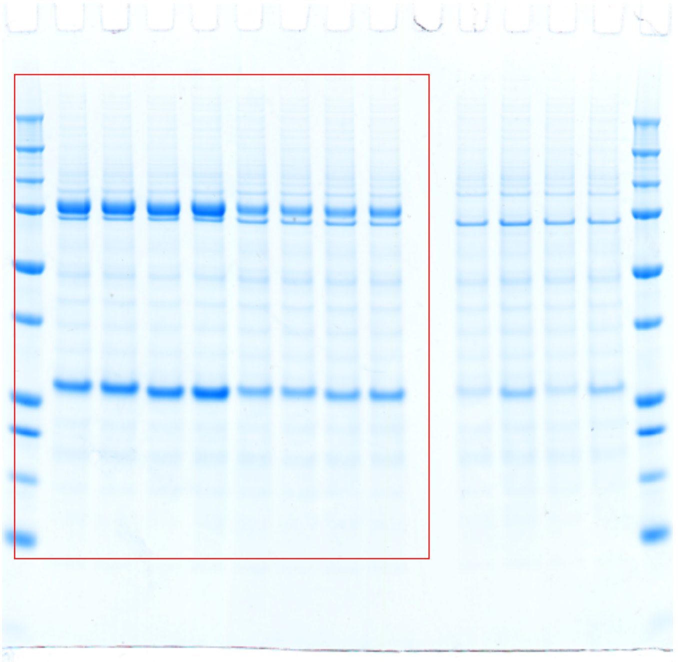

B

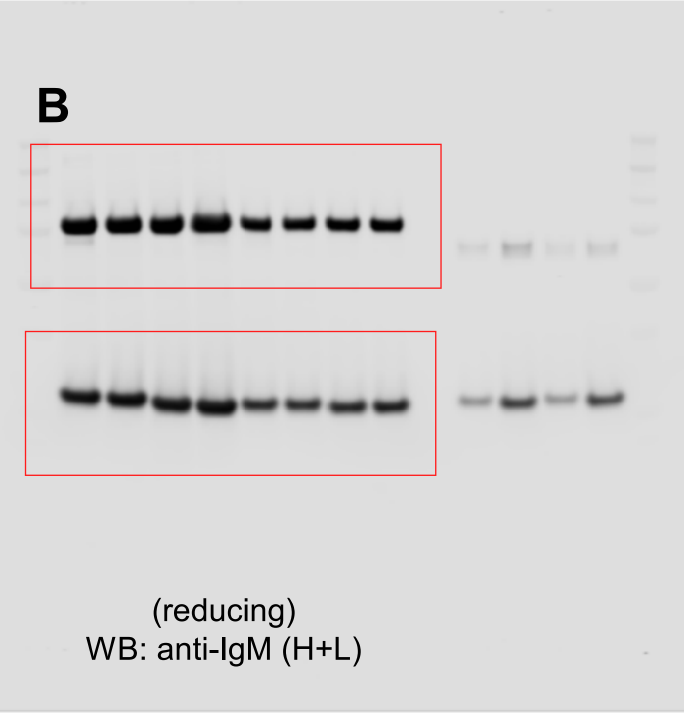

(reducing)
WB: anti-IgM (H+L)

B

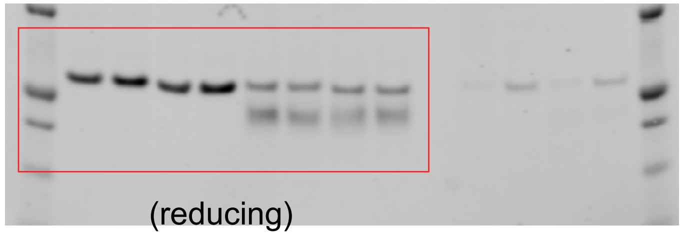

(reducing)
WB: anti-JC

C

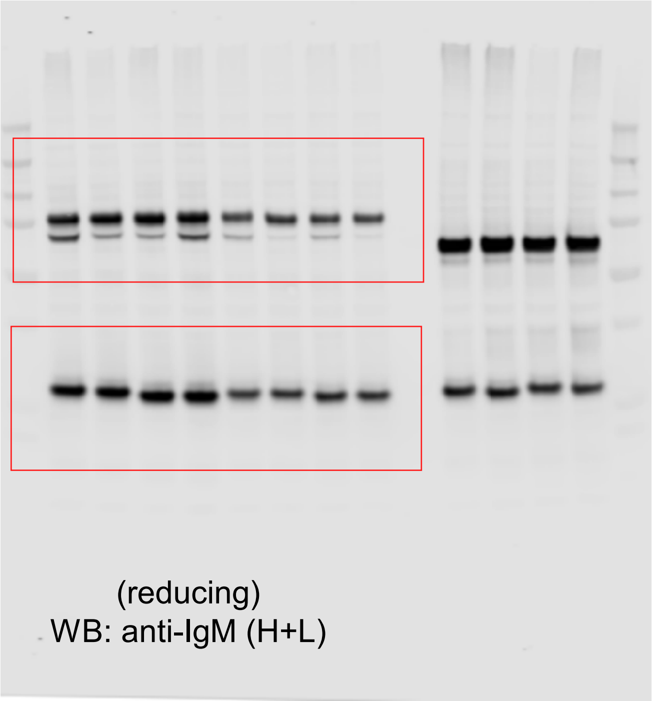

(reducing)
WB: anti-IgM (H+L)

C

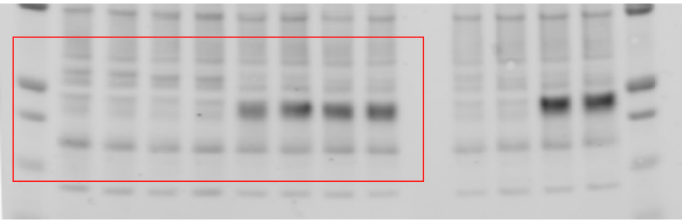

(reducing)
WB: anti-JC

S10 Fig D E F: The raw blots before the cropping are shown below. The cropped regions are shown in red box.

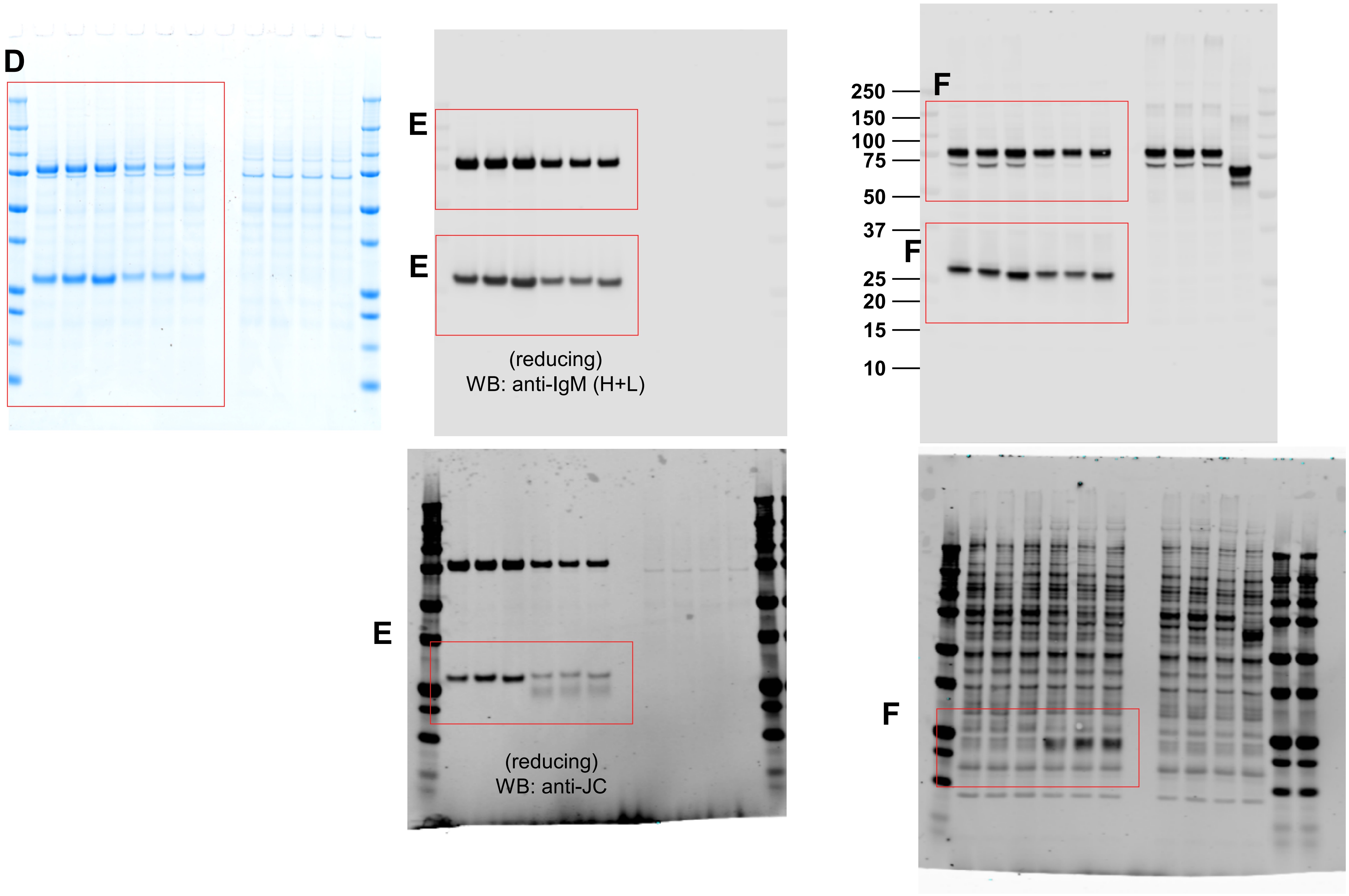

S10 Fig G H I J K: The raw blots before the cropping are shown below. The cropped regions are shown in red box.

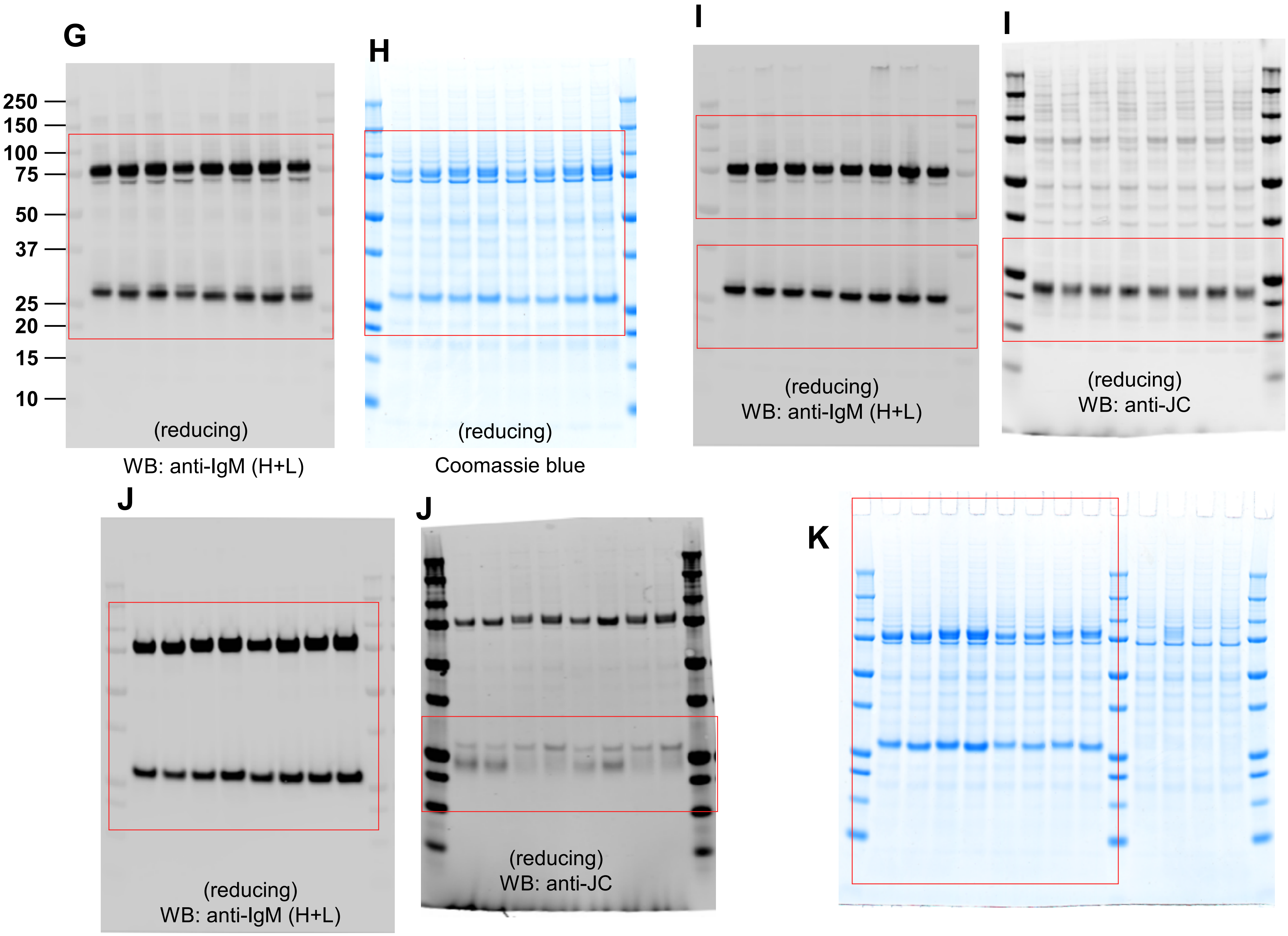

S10 Fig L M: The raw blots before the cropping are shown right. The cropped regions are shown in red box.

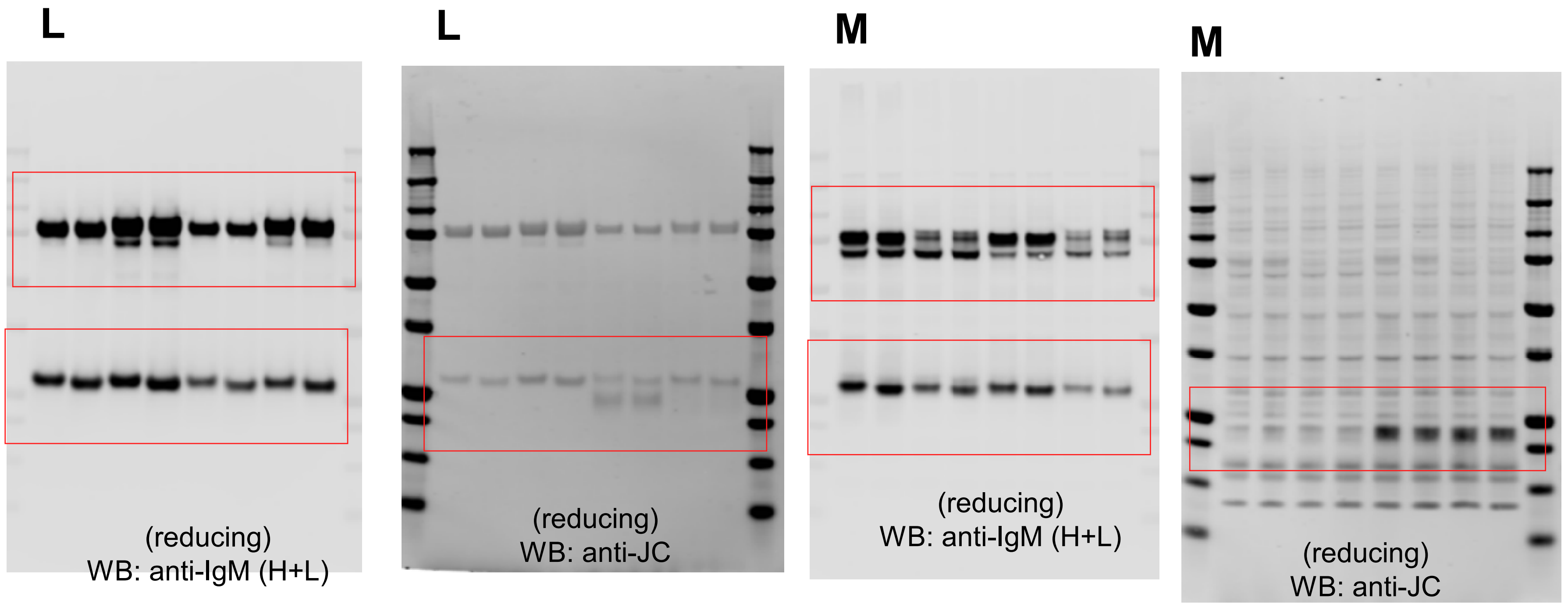

S11 Fig A B C: The raw gel and blot before the cropping are shown below. The cropped regions are shown in red box.

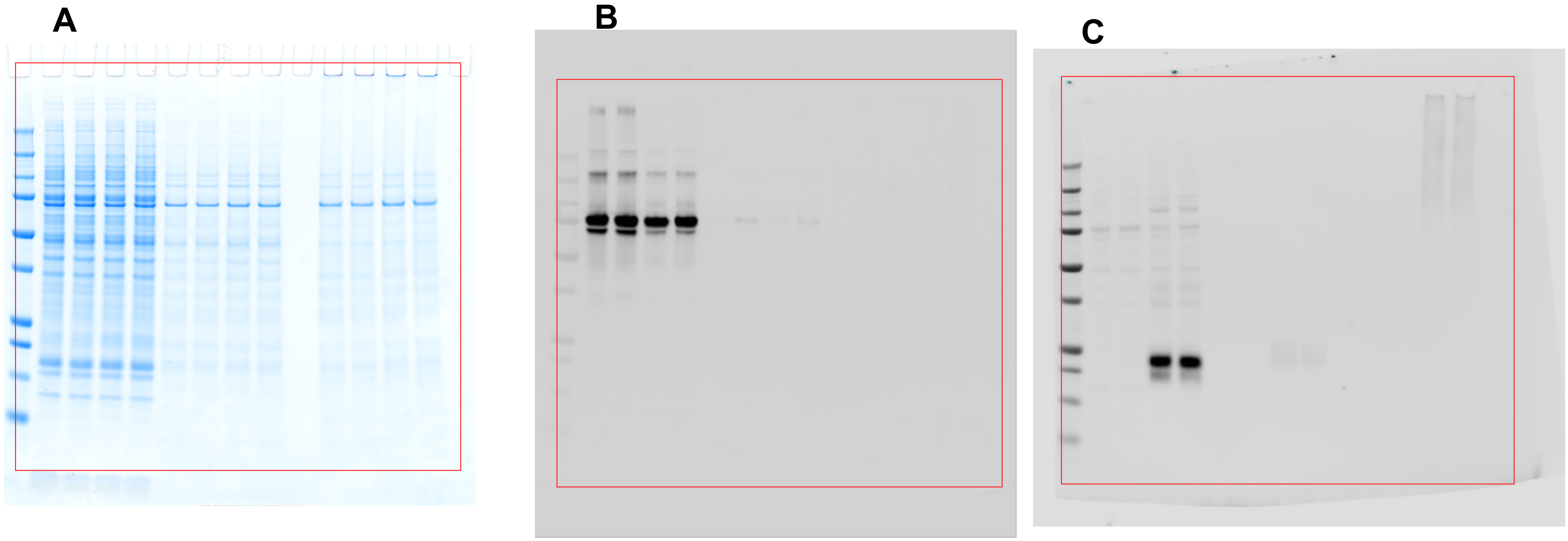

S12 Fig C D: The raw gels before the cropping are shown below. The cropped regions are shown in red box.

C

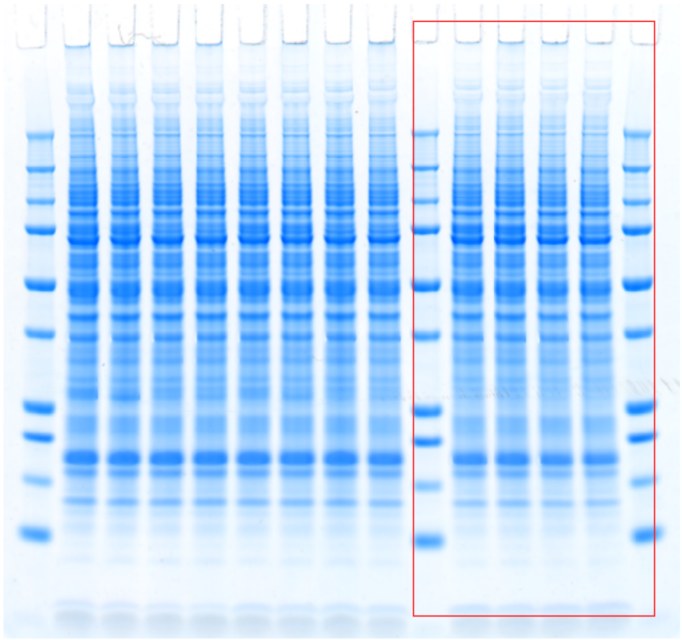

D

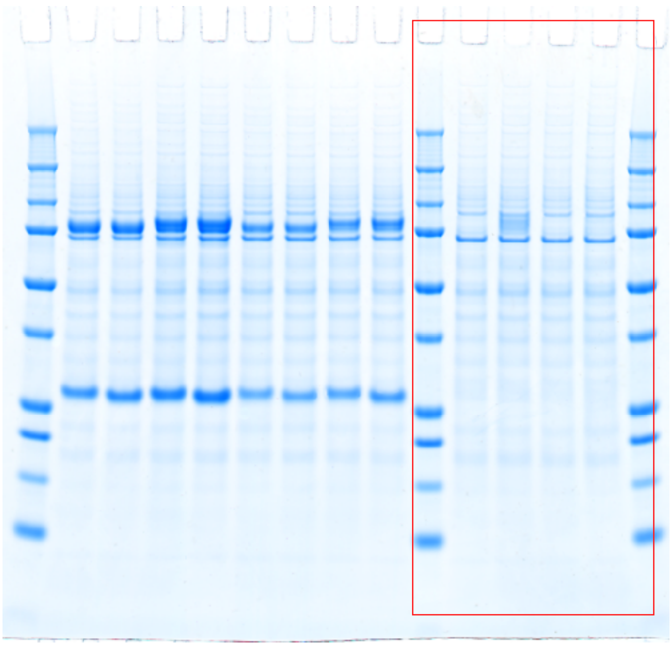

S12 Fig C D E: The raw blots before the cropping are shown below. The cropped regions are shown in red box.

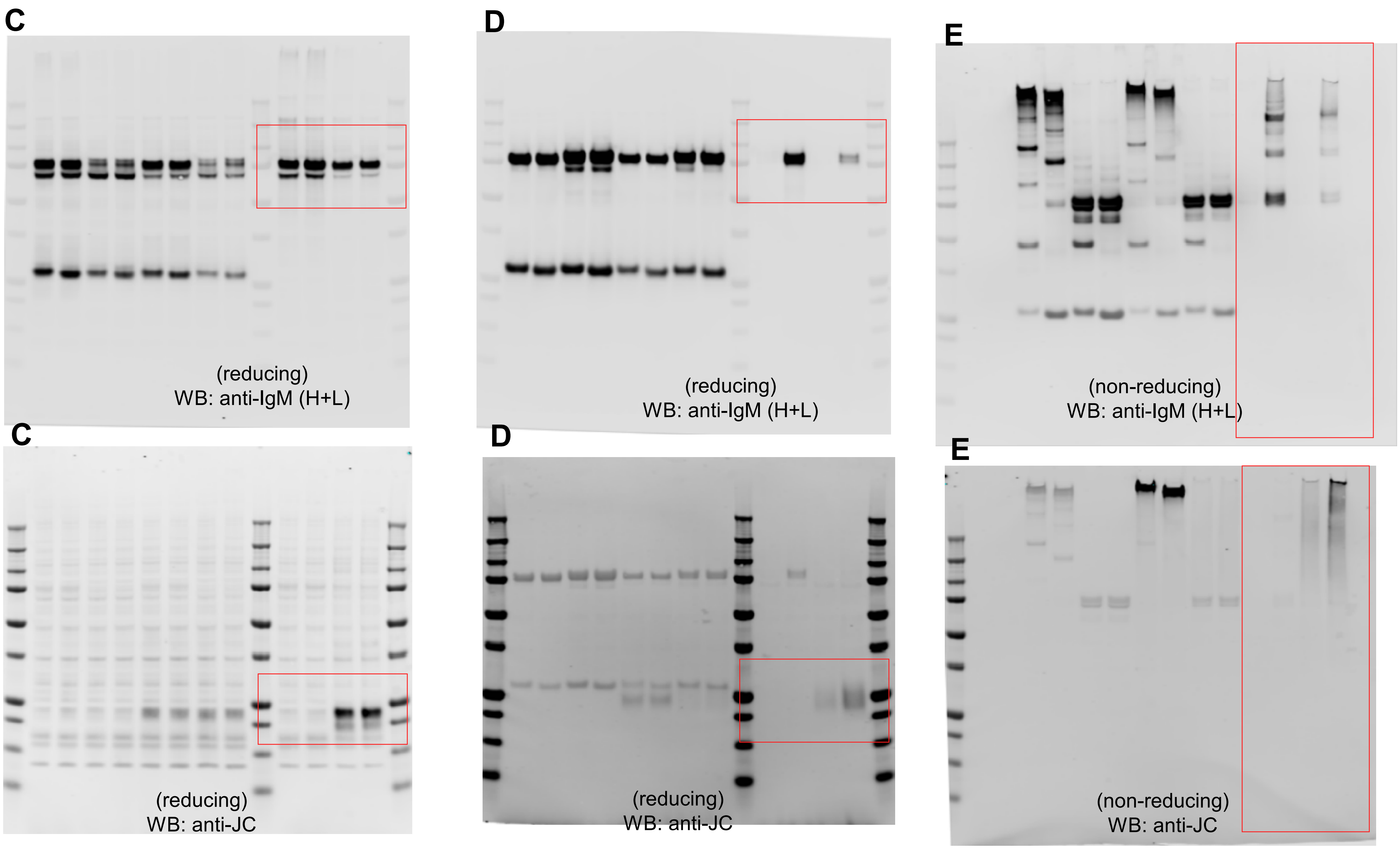

Supplement: S1 Raw images — (PDF) [file pone.0291568.s014.pdf]
