# Supplementary material for: Understanding the biosynthesis of human IgM SAM-6 through a combinatorial expression of mutant subunits that affect product assembly and secretion
Source: PLoS One. 2024 Jun 7;19(6):e0291568. doi: 10.1371/journal.pone.0291568 (PMC11161108; doi:10.1371/journal.pone.0291568)
Supplement: Attachment — Submitted filename: Hasegawa et al Revision 2024.docx [file pone.0291568.s015.docx]

In an extensive tour de force for both writers and readers (>100 pages, almost 30,000 words, 15 multipaneled main figures and 7 supplemental ones) Hasegawa et al. investigate the biogenesis of multimeric IgM. They address this interesting problem analyzing systematically a panel of heavy and light chain mutants, that yield 48 possible combinations.

While the biochemical analyses are clear and complete, the imaging studies (mainly immunofluorescence) leave room for improvement and clarification.

However, besides some technological issues that can be addressed with additional work, the main criticism that can be raised to this extensive study is its largely confirmatory nature.

That interchain H-L bonds are not needed for antibody assembly is known since over 50 years. In many species IgA exist that lack them. And monoclonal antibodies endured clonal selection without a covalent association between their H and L chains.

Large parts of the wordy text is devoted to refuting papers published by others rather than to highlighting novel findings. Nothing wrong with the desire to clarify potential controversies. It seems to this reviewer, however, that the authors see controversies where there are simply different experimental systems. In doing so, the authors do not address the potentially relevant reasons that underlie different phenotypes in different cells expressing different proteins.

That said, the wealth of data and thoroughness of this systematic approach would make a profoundly revised manuscript suitable for publication.

Below follows a list of specific points that the authors are expected to carefully address before this manuscript can be reconsidered for publication.

**Major points**

The text should be shortened drastically. The main points of the study can be summarized in much less than one third of the actual size of the text and iconography. Many of the main figures can become supplemental information. For example, the authors ‘validate’ the efficiency of transfection in most experimental set ups. Validation is obviously essential, but the data can be easily summarized once in supplementary material. As presented, the paper reads like a lab book: the authors should make the effort to pick the relevant information and focus the reader’s attention on the novel aspects of their diligent work.

In general, condensation in RBs correlates with the presence of abundant detergent insoluble material. The authors may wish to investigate further this problem and discuss why no such ‘precipitates’ be formed in their system.

Higher resolution imaging are needed for some key mutants analyzed (below some detailed suggestions)

-Page 2, line 56. In view of the existing literature, it is not at all unexpected that the H-L and inter-µ chain bonds linking C337 be disposable for polymerization. That C414 and C575 can mediate different bonds was also already shown (Giannone et al., 2022 and references therein). What is solid in this study is the ‘holistic’ analysis in a single human IgM. The authors should rewrite the Ms focusing on the few novel findings and highlighting the systematic confirmation of what was already known from different models.

-Page 15 , line 344 and Figure 1 c-d. The distribution of free µHC observed by immunofluorescence (IF) cells differs from other reports in which unassembled H chains were shown to distribute in the ER, colocalizing with BiP or other markers of the organelle. Therefore, identification of the red blobs containing µHC as Russell bodies is premature. Calnexin costaining (shown in Figure 4) is not consistent with a RB, as defined by the accumulation of amorphous Ig aggregates in an intracellular compartment. A membrane protein like calnexin would be expected to be excluded from the lumen of ER-localized RB.

Are these µHC detergent insoluble, as most condensed-aggregated proteins in Mott cells are?

The authors should use other organelle markers and perform electron microscopy and exclude the possibility that aggresomes be formed by their transfectants.

In lymphoid cells unassembled µ and J chains are dislocated to the cytosol and degraded very rapidly by proteasomes (Chillaron et al, 2000: Mancini et al, 2000; Fagioli et al 2001), but more slowly in HeLa (Medrano-Fernandez et al, 2014). The authors should compare the rate and mode of the degradation as well as the solubility of µHC and µHC-∆CH1 before concluding that both form similar structures when overexpressed in the absence of L chains.

In general, it would be nice to see bigger enlargements of the IF images, and more markers (ERGIC and Golgi subregions) to better appreciate the localization of the key mutants (not all of them, the authors should try to focus the reader’s attention on the ones that give unexpected results).

-p18, line 395. The authors should discuss why SAM µHC behave differently from other H chains (VH? Human vs mouse? Etc).

-p20 line 453. The authors describe these ‘cytoplasmic puncta’, without characterizing them. In the discussion, they raise the possibility of them being ER exit sites. These structures should be characterized, using additional markers, ideally by CLEM.

-pages 20-21; the authors go a long way to refute some ‘influential papers’ published on the biogenesis of RBs. In Ref. 69, Mattioli et al. use HeLa transfectants to show a role of ERGIC in catalyzing the condensation of murine µHC∆CH1 chains, possibly by increasing the local concentration above a critical point. They also show a role of L chains in determining the site of condensation (smooth RBs in ERGIC, no L chains and rough RBs, ribosome coated ER, with L chains). A reasonable interpretation was that the tendency of CL domains to form homodimers facilitate condensation. Accordingly, a VL domain alone did not induce rRB formation. Would expression of a VL only suffice to change the phenotypes (see also p25)?

The authors should consider that not only cell-specific factors (ERGIC53, ERp44, the redox pose of the ER etc., see Ronzoni et al. 2010), but also subtle determinants in other domains, mainly the Variable ones, would dictate the rate, extent and localization of condensation/precipitation. These points should be further analyzed, as the differences between experimental systems may reveal novel phenomena, or at least constructively discussed.

P21, line 462. The potential ole of JC in favoring µHC-∆CH1 is of interest. Considering its tendency to form intra- and inter-chain bonds (see the smear secreted by HEK transfectants) JC may serve a ‘cross-linking’ role as described for L chain by Mattioli et al (2006).

-p25, line 555. The authors should consider expressing a VL alone or swapping VH domains. What is their role in catalyzing aggregation?

-p27, line597. It is of interest that JC alone did not form RBs but was trapped in ‘RB-like’ structures when µHC were there. Are JC degraded more rapidly when not trapped in RBs?

-p35 line 761. The nature and significance of these droplets should be explained and discussed.

-p36 line 791. Why is the JC smear no longer visible?

-p37 line 825. There are many things that one does not realize when submitting a manuscript. Papers are published if they contain original and important observation(s), like for instance that the absence of the BiP-binding CH1 is generally sufficient to induce intracellular condensation of immunoglobulins. The original observations are then confirmed (e.g. the Bruggeman lab showing that mice lacking L chains have Mott cells plenty of Ig lacking a CH1) and extended by the observation that J chains are trapped in µHC and µHC-∆CH1 ‘RBs’. Are JC detergent insoluble? Are JC trapped in IgG-containing RBs?

A potentially interesting point to follow up would be to determine whether µHC form mixed polymers with µHC-∆CH1 and are trapped in RBs. It remains possible that the two end up in different blobs.

-p38 line 827. The authors state *….it was clear that covalent aggregation between JC and µHC-∆CH1 .. was the underlying…* the logic of this conclusion is not clear. As the authors elegantly confirm, JC are not needed to form RBs.

-p40 line 875. Sec MALS or sucrose gradient analyses should be described here to sustain the case. By the way, that the inter H-L bond is not necessary was known for 5 decades.

-p42 line 912. Can the authors exclude that SS reshuffling occurs after secretion.

An interesting endeavor -also considering the long discussion about the potential medical use of IgM variants- would be to compare the stability of the mutants with or without interchain bonds.

-p52 line 1133. The data suggest that unassembled µHC avoid BiP quality control ad are secreted. How do the authors explain this unexpected finding?

Some sections of the discussion sounds more like parts of a grant. Although probably tenable, some speculations are not sustained by data or elements that derive from this paper and could be deleted.

**Minor points**

-p16 line 365. Hexamers represent the vast majority of secreted IgM. Wouldn’t ELISA allow to quantify them?

-p17, lines 375 and following. Many previous studies showed that IgM can be assembled and secreted by non-lymphoid cells. Of interest, HeLa variants were isolated that failed to do so and secreted also non polymeric IgM subunits. Retention of intermediates was partially rescued by ERp44 and ERGIC53 overexpression, suggesting a defect in distal quality control. This is another example of how different cell sublines handle proteins differently. The unexpected can lead to novel findings, without the need to think that others made mistakes.

-p19 line 423: the sentence *‘…induces a spontaneous…*’ sounds somehow contradictory

-p23, line 500 and following. The dual role of CH1-BiP and C575-ERp44 was shown over 30 years ago and confirmed in many further studies.
