# Supplementary material for: Understanding the biosynthesis of human IgM SAM-6 through a combinatorial expression of mutant subunits that affect product assembly and secretion
Source: PLoS One. 2024 Jun 7;19(6):e0291568. doi: 10.1371/journal.pone.0291568 (PMC11161108; doi:10.1371/journal.pone.0291568)
Supplement: Attachment — Submitted filename: Response to Reviewers.docx [file pone.0291568.s016.docx]

PONE-D-23-28036

Understanding the biosynthesis of human IgMs through a combinatorial expression of mutant subunits that affect different assembly steps.

PLOS ONE

Dear Dr. Hasegawa,

Thank you for submitting your manuscript to PLOS ONE. After careful consideration, we feel that it has merit but does not fully meet PLOS ONE’s publication criteria as it currently stands. Therefore, we invite you to submit a revised version of the manuscript that addresses the points raised during the review process.

1) While both reviewers appreciate the enormous amount of data generated in this study, it overwhelms any reader.  I strongly recommend to focus on the novel findings, and to condense the confirmatory data into supplemental data/figures.  The whole manuscript should be streamlined and significantly shortened.

We shortened the text from ~30,000 words to ~23,000 words (including the Supplemental legends and references), although we added several new data figures requested by both reviewers. We also used Supplemental materials more effectively. Because IgMs are often considered a very complex group of secretory glycoproteins, we wanted to ensure we established a suitable experimental platform so that we could start asking questions on IgM biosynthesis to uncover something novel. It was a natural process to build upon many of the known important concepts that laid the foundation of IgM assembly mechanisms. This is an important thing to do, as Reviewer #2 pointed out that “differences in employed expression systems can play roles in revealing unexpected novel phenomena.” Instead of treating the data as “confirmatory” and sending them to Supplement, I decided to keep some of them in the main Figures.

It was also important to show all the cards because the cell line we used is highly relevant in antibody drug discovery research and for antibody therapeutics production in the biotech industry in general.

2) Both reviewers are also concerned about the discussion of some seemingly controversial data that could simply be explained by differences in the experimental systems.  These extensive discussion also distract from the main novel findings and I suggested to condense respective sections.

As suggested, we removed unnecessary arguments and discussions when explaining some conflicting results.

3) Address all the concerns of the reviewers by making appropriate changes to the text and provide additional experimental data if required to support the current conclusions.

We performed detergent solubility analysis for the Russell body-like aggregates as suggested by Reviewer #2 (see Suppl. 10). We also performed two additional IF imaging studies. (1) Characterization of the IgM-positive punctate structures by co-staining with ERGIC and ER exit site markers (see Suppl. 1). (2) Characterization of Russell body-like aggregates by co-staining with additional ER resident makers such as BiP, ERp57, and calreticulin, as well as cis- and trans-Golgi markers such as giantin and p230 (see Suppl. 2 and Suppl. 3).

Regrettably, due to our organizational technical limitations, we were not able to perform one suggested transmission EM and CLEM study to characterize the IgM-containing cytosolic puncta.

Please submit your revised manuscript by Mar 18 2024 11:59PM. If you will need more time than this to complete your revisions, please reply to this message or contact the journal office at [plosone@plos.org](mailto:plosone@plos.org). When you're ready to submit your revision, log on to <https://www.editorialmanager.com/pone/> and select the 'Submissions Needing Revision' folder to locate your manuscript file.

Thank you for extending the deadline to April 15, 2024.

Please include the following items when submitting your revised manuscript:

- A rebuttal letter that responds to each point raised by the academic editor and reviewer(s). You should upload this letter as a separate file labeled 'Response to Reviewers'.
- A marked-up copy of your manuscript that highlights changes made to the original version. You should upload this as a separate file labeled 'Revised Manuscript with Track Changes'.
- An unmarked version of your revised paper without tracked changes. You should upload this as a separate file labeled 'Manuscript'.

If you would like to make changes to your financial disclosure, please include your updated statement in your cover letter. Guidelines for resubmitting your figure files are available below the reviewer comments at the end of this letter.

If applicable, we recommend that you deposit your laboratory protocols in protocols.io to enhance the reproducibility of your results. Protocols.io assigns your protocol its own identifier (DOI) so that it can be cited independently in the future. For instructions see: [https://journals.plos.org/plosone/s/submission-guidelines#loc-laboratory-protocols](https://nam11.safelinks.protection.outlook.com/?url=https%3A%2F%2Fjournals.plos.org%2Fplosone%2Fs%2Fsubmission-guidelines%23loc-laboratory-protocols&data=05%7C02%7Charukih%40amgen.com%7Cf8ebdc5e72544e2baebe08dc242dca01%7C4b4266a6136841afad5a59eb634f7ad8%7C0%7C0%7C638425026629295153%7CUnknown%7CTWFpbGZsb3d8eyJWIjoiMC4wLjAwMDAiLCJQIjoiV2luMzIiLCJBTiI6Ik1haWwiLCJXVCI6Mn0%3D%7C0%7C%7C%7C&sdata=abHngEop9f4NK5BPJ2pay8TzbsoJvoPoDWqGOxoxkP4%3D&reserved=0). Additionally, PLOS ONE offers an option for publishing peer-reviewed Lab Protocol articles, which describe protocols hosted on protocols.io. Read more information on sharing protocols at [https://plos.org/protocols?utm_medium=editorial-email&utm_source=authorletters&utm_campaign=protocols](https://nam11.safelinks.protection.outlook.com/?url=https%3A%2F%2Fplos.org%2Fprotocols%3Futm_medium%3Deditorial-email%26utm_source%3Dauthorletters%26utm_campaign%3Dprotocols&data=05%7C02%7Charukih%40amgen.com%7Cf8ebdc5e72544e2baebe08dc242dca01%7C4b4266a6136841afad5a59eb634f7ad8%7C0%7C0%7C638425026629306444%7CUnknown%7CTWFpbGZsb3d8eyJWIjoiMC4wLjAwMDAiLCJQIjoiV2luMzIiLCJBTiI6Ik1haWwiLCJXVCI6Mn0%3D%7C0%7C%7C%7C&sdata=0mI%2FcvSGdMlVMxMtOTp9y4DNfO7TYQuQmjfoYNkZoqU%3D&reserved=0).

We look forward to receiving your revised manuscript.

Kind regards,

Sebastian D. Fugmann, Ph.D.
Academic Editor
PLOS ONE

-----------------------------------------------------------------------------

**Journal Requirements:**

When submitting your revision, we need you to address these additional requirements.

1. Please ensure that your manuscript meets PLOS ONE's style requirements, including those for file naming. The PLOS ONE style templates can be found at

https://journals.plos.org/plosone/s/file?id=wjVg/PLOSOne_formatting_sample_main_body.pdf and

<https://journals.plos.org/plosone/s/file?id=ba62/PLOSOne_formatting_sample_title_authors_affiliations.pdf>

2. Note from Emily Chenette, Editor in Chief of PLOS ONE, and Iain Hrynaszkiewicz, Director of Open Research Solutions at PLOS: Did you know that depositing data in a repository is associated with up to a 25% citation advantage (https://doi.org/10.1371/journal.pone.0230416)? If you’ve not already done so, consider depositing your raw data in a repository to ensure your work is read, appreciated and cited by the largest possible audience. You’ll also earn an Accessible Data icon on your published paper if you deposit your data in any participating repository (<https://plos.org/open-science/open-data/#accessible-data>).

3. We note that you have included the phrase “data not shown” in your manuscript. Unfortunately, this does not meet our data sharing requirements. PLOS does not permit references to inaccessible data. We require that authors provide all relevant data within the paper, Supporting Information files, or in an acceptable, public repository. Please add a citation to support this phrase or upload the data that corresponds with these findings to a stable repository (such as Figshare or Dryad) and provide and URLs, DOIs, or accession numbers that may be used to access these data. Or, if the data are not a core part of the research being presented in your study, we ask that you remove the phrase that refers to these data.

The “data not shown” issue was addressed by actually showing the data as the new figure sets, as shown in Fig. 12 and Suppl. 11.

4. We notice that your supplementary figures are included in the manuscript file. Please remove them and upload them with the file type 'Supporting Information'. Please ensure that each Supporting Information file has a legend listed in the manuscript after the references list.

We followed these instructions.

-----------------------------------------------------------------------------

**Reviewer #1:**

The authors present a very thorough study on IgM assembly. A large number of mutants have been studied and the findings obtained largely support current models for IgM assembly. The new findings are buried in the impressive data set. While it is of value to repeat previous findings as controls, the new insight needs to be better highlighted. All in all the manuscript is a very long read and therefore should be shortened. The story and main message is missing/unclear. Many of the data is a reproduction of previous findings. Some aspects are new and interesting – the authors should elaborate more on them. Some conclusions do not seem to be correct and should be revisited (chapter 3.12). In general, the manuscript would benefit from shortening and reorganizing.

**Specific points**

1. Mutants that confirm previous findings should be summarized in the supplementary information.

This point was addressed to the best we can without compromising our intentions for this research.

Because IgMs are very complex secretory glycoproteins, we wanted to ensure we first established a suitable experimental platform so that we could start asking questions on IgM biosynthesis to uncover something new. It was a natural process to go through many of the known important concepts in our own assay and protein expression systems.

We judged it was too presumptuous to assume that a series of concepts proposed during the past 30 years (by using different clones and sources of IgMs from different species, different assays, and different cell types, etc.) are automatically recapitulated in our natural human IgM—partly because we don’t use the same assays that investigators in the 80s and 90s had used. We really needed to see how things could translate if we used our assay methods in our cell hosts. This is pointed out by Reviewer #2 in the comment, “as the differences between experimental systems may reveal novel phenomena”. Nonetheless, I tried to address this the best I could.

The cell line we used, HEK293(6E), is widely used in the biotech industry as a preferred host to produce recombinant immunoglobulins during early drug discovery. It would be beneficial for the biotech community to fully demonstrate the potential of this cell host in making a complex molecule like IgM and how the cells respond to the demand of IgM assembly. Plus, our expression platform allowed us to switch the production of hexamers and pentamers qualitatively, which helped us to test the effects of mutations in hexamer and pentamer in a more controlled fashion.

2. The text needs to be shortened. Often experiments and results are described in too much detail and length. For example, one could state that μHC expressed alone always formed Russel bodies regardless of mutations – with the exception of 4x C>S. A description of each mutant in this context is overwhelming if no additional effects are visible.

Agreed not to describe experiments and results in too much detail and length.

3. 3.1 - The first paragraphs are meticulously performed proofs of principle. I suggest shifting these to the supplements, as they do not contain new insights into the topic of IgM biosynthesis and assembly. Figure 1 and 2 belong in the supplement section. The same is true for 3.2. Please highlight the one difference you spotted when co-expressing μ-HC-ΔCH1 with λLC. This information gets lost in the presence of the list of already known facts. The corresponding figures should be moved to the supplement. Please only show images for new findings.

The reviewer’s points are carefully considered. The other reviewer (Reviewer #2), however, requested us to show more characterization of IgM puncta by co-staining with the ER exit site or ERGIC markers to identify the structure. Likewise, Reviewer #2 asked us to co-stain the μHC induced Russell body with additional ER and Golgi markers. Furthermore, Reviewer #2 specifically asked us to determine the detergent solubility of intracellular μHC and its mutants.

As such, instead of sending Fig. 1 and Fig. 2 to supplement, I decided to reorganize the figure panels by removing unnecessary images and gels, while adding new data that Reviewer #2 requested. I hope I hit a reasonable mid-point that addressed the feedback from both reviewers.

4. The finding that Russel bodies are not formed by μ-HC-ΔCH1 C414/575S, which also is secretion competent, should be highlighted.

Followed the reviewer’s suggestion. I also added the detergent solubility data suggested by the other reviewer at the same time to highlight the rescue from Russell body, acquisition of detergent solubility, and restoration of secretion.

5. Please refer to this paper: <https://doi.org/10.15252/embj.2021108518> in the context of chapter 3.5

Although the reviewer suggested citing Giannone et al. (2022) 's paper in chapter 3.5, I found Chapter 3.4 is a more suitable place to cite it. This paper addresses why the C575S mutation decreases the efficiency of covalent polymeric IgM formation by blocking the formation of transitory non-native disulfide required for IgM polymerization.

6. The titration of JC DNA and the correlation with the amount of pentamer is a very nice experiment and should get way more spotlight in the context of this paper.

By following this reviewer’s suggestion, I rewrote section 3.6. I also modified the Abstract and Intro to capture this. Thank you for the suggestions.

7. It is interesting, that you were able to secrete JC in the absence of μHC and λLC – something that was not reported before. This finding is worth more investigation.

I agree. The free JC secretion has not been widely known. We are in the midst of characterizing this enigmatic JC subunit behavior in more detail for a future manuscript.

Incidentally, Kawasaki et al (2024) just published a paper in PNAS reporting the function of JC as a chemokine (see link below). There are many things we still do not know about JC subunit.

<https://doi.org/10.1073/pnas.2318995121>

8. 3.7 - The presence of pentamers and hexamers has been already published.

Yes. The presence of pentamer and hexamer mixtures has been known for a long time. In fact, I cited relevant papers in this section to reflect that. However, it has never been shown as clearly as this experiment that we can qualitatively switch hexamer production to pentamer production by simply titrating transfecting the DNAs of each subunit. In many studies, the investigators do not regulate the production of hexamer or pentamers, and some investigators report JC-free IgM pentamers that we did not detect in our expression platform. We might have benefited from this robust and preferred recombinant expression system widely employed in the biotech industry.

9. 3.8 - This section needs to be shortened and many data should be presented as supplementary information

I followed this reviewer’s suggestion as much as possible. The other reviewer suggested that I examine the detergent solubility of the JC subunit in the presence of other co-transfected subunit(s) in various combinations. As a result, it was challenging to make this section short, but I still tried.

10. The new finding about JC as part of Russel bodies depending on the presence of other chains is interesting. When CH1 is deleted, the effects stay the same. The evidence could be moved to the supplement and the text shortened.

The co-aggregation of JC into Russell body (of μHC or μHC-ΔCH1) was dependent on the Cys-575 in the absence of an LC subunit.

I tried to shorten the text and use the supplement as much as possible.

11. 3.9 - It is already known, that C575 and C414 are necessary for correct pentamer assembly. C575 has the more important role. For example Hiramoto et. al. (DOI: 10.1126/sciadv.aau1199) already showed that for the C414S mutation, in addition to ‘loose’ pentamers, also tetramers can be formed. The cause, why the oligomers are not formed in your setting is probably the absence of C575.

We included analytical SEC data (directly performed on harvested culture media) for C414S, C575S, and C414/575S mutants, both in hexameric and pentameric IgM expression settings.

This SEC data also showed some mutants that could not assemble covalent IgM polymer still assembled polymeric IgMs partly through non-covalent protein-protein interactions.

12. 3.10 - The results with ΔCS mutant are very interesting. However, the length of the description is too long.

Followed the reviewer’s suggestion and shortened the section as much as I could.

13 3.11 - This section could be combined with the previous chapter, where you state that the covalent link between CH1 and LC is not strictly necessary for hexamer/pentamer formation. This section is also too long. The summaries at the end of each chapter seem sufficient to present the data.

I carefully considered this option of combining this with the previous section. I decided to keep them separate but did my best effort shortening it.

14. 3.12 - The C337S mutant was described previously in several publications; e.g. Wiersma et. al. described formation of hexamers and pentamers with this mutant, which of course dissociate on SDS-PAGE (<https://doi.org/10.4049/jimmunol.160.12.5979>). This is also reasonable, as single Cμ4tp domains are able to form hexamers in vitro (<https://doi.org/10.1073/pnas.1701797114>), which of course can only be observed with non-denaturing approaches like SEC. Most likely, the μ2λ2JC and μ2JC are part of such a non-covalent pentamer, which is dissociated by SDS-PAGE. The explanation proposed in the manuscript seems unlikely. Analysis of this mutant in the presence of LC AND JC by SEC seems needed. Formation of hexamers without JC is seen in Figure 14, chromatogram 6 but there are no intermediates between the monomer and hexamer.

Much more diverse types of covalently assembled intermediates were found for the C337S mutant in non-reducing SDS-PAGE. Various “loosely” associated intermediates probably exist in the same sample, too, as this reviewer pointed out. SEC worked relatively well for the C337S mutant hexamer analysis; however, when we ran analytical SEC for the C337S mutant pentamer in the harvested culture media, we kept obtaining too many small broad “ripples,” and the data were not interpretable.

As this reviewer suggested, if μ2λ2J and μ2J were part of such loosely associated non-covalent pentamers, the subunit stoichiometry of such loose pentamers would be very heterogeneous and different from that of canonical pentamers. For example, some loosely associated pentamers may have more than one JC subunit in the loosely associated complexes. If this is actually the case, our uninterpretable SEC data may simply reflect such facts.

Reflecting on the reviewer’s inputs, I deleted my previous conclusion and rewrote this section.

15. 3.13 - The last paragraph of this chapter describes the effect of the 4x C>S mutant well (line 1177-1180), but again the text needs to be shortened.

Previous section 3.13 is now section 3.14. Section 3.14 was shortened as much as I could.

16. 3.14 - Please show all the chromatograms together with the SDS gels of the more important mutants.

Because all the gels were shown in previous sections, I decided to focus on SEC data in the new Figure 14. I also re-arranged the orders of chromatographs. The SEC results for C414S mutant was “data not shown” in the original submission, but they are now shown (see Fig. 14A).

For the 3-chain transfection (pentameric IgMs), I am now showing the SEC data for C414S, C575S, C414/575S with λLC or λLC-ΔCS (see Fig. 14C). The results for C337S mutant are not shown because of the technical difficulties as I explain in the response above.

-----------------------------------------------------------------------------

**Reviewer #2:**

In an extensive tour de force for both writers and readers (>100 pages, almost 30,000 words, 15 multipaneled main figures and 7 supplemental ones) Hasegawa et al. investigate the biogenesis of multimeric IgM. They address this interesting problem analyzing systematically a panel of heavy and light chain mutants, that yield 48 possible combinations.

While the biochemical analyses are clear and complete, the imaging studies (mainly immunofluorescence) leave room for improvement and clarification.
However, besides some technological issues that can be addressed with additional work, the main criticism that can be raised to this extensive study is its largely confirmatory nature. That interchain H-L bonds are not needed for antibody assembly is known since over 50 years. In many species IgA exist that lack them. And monoclonal antibodies endured clonal selection without a covalent association between their H and L chains.

In my original discussion, I already discussed the HC-LC inter-chain disulfide bonds in other species and Ig classes. The observation has not been reported in IgMs of higher vertebrates.

Large parts of the wordy text is devoted to refuting papers published by others rather than to highlighting novel findings. Nothing wrong with the desire to clarify potential controversies. It seems to this reviewer, however, that the authors see controversies where there are simply different experimental systems. In doing so, the authors do not address the potentially relevant reasons that underlie different phenotypes in different cells expressing different proteins.

The reviewer’s comments are taken. Any texts that might have suggested refuting prior work were removed completely from the revised manuscript.

That said, the wealth of data and thoroughness of this systematic approach would make a profoundly revised manuscript suitable for publication.

Below follows a list of specific points that the authors are expected to carefully address before this manuscript can be reconsidered for publication.

**Major points**

The text should be shortened drastically. The main points of the study can be summarized in much less than one third of the actual size of the text and iconography. Many of the main figures can become supplemental information. For example, the authors ‘validate’ the efficiency of transfection in most experimental set ups. Validation is obviously essential, but the data can be easily summarized once in supplementary material. As presented, the paper reads like a lab book: the authors should make the effort to pick the relevant information and focus the reader’s attention on the novel aspects of their diligent work.

I added a new set of data by following this and other reviewer’s suggestions but reduced the overall length by 23%. I also reorganized some figures and used Supplement more extensively.

In general, condensation in RBs correlates with the presence of abundant detergent insoluble material. The authors may wish to investigate further this problem and discuss why no such ‘precipitates’ be formed in their system. Higher resolution imaging are needed for some key mutants analyzed (below some detailed suggestions)

The suggested detergent solubility assay was performed by using the protocol I used previously in a different study. (DOI: 10.1016/j.bbamcr.2012.06.015)

The data are shown in Fig. 1G and Suppl. 5.

-Page 2, line 56. In view of the existing literature, it is not at all unexpected that the H-L and inter-µ chain bonds linking C337 be disposable for polymerization. That C414 and C575 can mediate different bonds was also already shown (Giannone et al., 2022 and references therein). What is solid in this study is the ‘holistic’ analysis in a single human IgM. The authors should rewrite the Ms focusing on the few novel findings and highlighting the systematic confirmation of what was already known from different models.

Yes, this is true. In the past 30 years, all the constant region Cys residues participating in inter-chain disulfide bonds have been mutated and tested. These studies helped generate the current model of IgM assembly, using various sources of IgMs and different assay methods. In individual studies, however, investigators tested a subset of Cys residues while different assay methods were used to measure different aspects. Some investigators studied hexamers while others studied the mixture of hexamers and pentamers. Different cell types (both homologous and heterologous) were used by different investigators. What is important in this study is to use one clinically relevant natural human IgM and a widely used recombinant system in biotechnology to produce various recombinant antibodies for therapeutic use. The system we employed can qualitatively switch hexamer and pentamer production at will by simply changing the transfecting subunit ratios. We were able to systematically test all important Cys residues both in hexameric and pentameric contexts—in terms of their biosynthesis and product quality.

To reflect this reviewer’s comment in the revision, I modified the Abstract and Introduction, as well as the rest of the texts as needed. Thank you.

-Page 15 , line 344 and Figure 1 c-d. The distribution of free µHC observed by immunofluorescence (IF) cells differs from other reports in which unassembled H chains were shown to distribute in the ER, colocalizing with BiP or other markers of the organelle. Therefore, identification of the red blobs containing µHC as Russell bodies is premature. Calnexin costaining (shown in Figure 4) is not consistent with a RB, as defined by the accumulation of amorphous Ig aggregates in an intracellular compartment. A membrane protein like calnexin would be expected to be excluded from the lumen of ER-localized RB.

In our first-hand experience, free γHC and free μHC can be distributed to reticular ER without aggregating into “blobs” if we use the classical CMV-based mammalian expression vectors or other more attenuated expression vectors. However, if we use a modern expression vector that can sustain a much higher level of gene/protein expression than the classical CMV vector, free HCs aggregate into those blobs depending on the intrinsic properties of HC subunits. There are a few “privileged” HCs that do not aggregate into a blob even under those hyper-overexpression settings. This has been shown using recombinant IgG mAbs.

So, the difference between ER distribution and blob formation can depend on at least two factors: (1) the HC’s intrinsic physicochemical properties determined by the primary sequence and others, and (2) the sheer expression level that can be achieved by the employed expression vector system.

We have been using an expression system (cell host and vector) developed in NRCC, which is widely used in the biotech industry to produce large quantities of recombinant antibodies.

Regarding the calnexin co-aggregation with the blob, our thoughts are explained in the section below.

We also start using the term “Russell body-like” globular aggregates in case this reviewer is not comfortable with our using the term, Russell body, definitively. The other reviewer seems to have no problems though.

Are these µHC detergent insoluble, as most condensed-aggregated proteins in Mott cells are?

We performed the suggested detergent solubility assay (Fig. 1G and Suppl. 5). Yes, these μHC blobs are detergent insoluble.

The authors should use other organelle markers and perform electron microscopy and exclude the possibility that aggresomes be formed by their transfectants.

We addressed this reviewer’s concern by performing additional co-staining experiemnts (see Suppl. 2 and Suppl. 3). Tested markers are SEC13, SEC31A, BiP, ERp57, calreticulin, giantin, p230, and LAMP2 in addition to the original calnexin (an ER membrane-anchored protein).

In our original submission, we co-stained with calnexin---which was repeatedly shown to co-localize (or co-aggregate) with RB-like aggregates despite calnexin being an ER membrane protein, not a lumenal protein.

Examples:

https://doi.org/10.1016/j.bbamcr.2012.06.015

https://doi.org/10.1080/19420862.2017.1314875

We believe calnexin and nascent calnexin can still co-aggregate with the aggregating HC subunit in the ER lumen. Calnexin is a type-I membrane protein with residues 21-481 is lumenal and 482-501 is TM while 503-592 is cytosolic. The major portion of the calnexin protein is located in the lumen. Conceptually it is strange that calnexin co-aggregate with the misfolding HC subunits in the ER lumen, but the same observations were made repeatedly. There is a marginal possibility that our rabbit polyclonal anti-calnexin is cross-reacting with its close homolog, calreticulin, an ER lumenal protein.

As shown previously and in Suppl. 2 and 3, the aggregating HCs also co-aggregated with typical ER lumenal proteins such as BiP, calreticulin, and ERp57. After being convinced that calnexin staining could be conveniently used to co-stain Russell body, I took it for granted without considering what other investigators would think about our observation.

Because the “blobs” are clearly associated with the ER, we did not perform EM studies to exclude the possibility of them being aggresomes.

In lymphoid cells unassembled µ and J chains are dislocated to the cytosol and degraded very rapidly by proteasomes (Chillaron et al, 2000: Mancini et al, 2000; Fagioli et al 2001), but more slowly in HeLa (Medrano-Fernandez et al, 2014). The authors should compare the rate and mode of the degradation as well as the solubility of µHC and µHC-∆CH1 before concluding that both form similar structures when overexpressed in the absence of L chains.

Both µHC (Suppl. 2) and µHC-∆CH1 (Suppl. 3) aggregate into the Russell body-like globular “blobs” in the ER lumen, and both µHC and µHC-∆CH1 formed detergent insoluble particulates (Suppl. 5).

While the rate and mode of the degradation may be interesting additional features to investigate, I feel the morphological data and detergent insolubility are sufficient for us to say both µHC and µHC-∆CH1 aggregated in the ER in the absence of L chains.

In general, it would be nice to see bigger enlargements of the IF images, and more markers (ERGIC and Golgi subregions) to better appreciate the localization of the key mutants (not all of them, the authors should try to focus the reader’s attention on the ones that give unexpected results).

The original Fig. 1A images were replaced with new ones. In additions, we show new set of co-staining experiments of IgM punctate structures using SEC13, SEC16A, SEC31A, BiP, calnexin, giantin, p230, NPC1, and LAMPS2 (Suppl. 1). Likewise, we performed a new set of co-staining work using additional organelle markers for µHC blobs (Suppl. 2) and µHC-∆CH1 blobs (Suppl. 3).

-p18, line 395. The authors should discuss why SAM µHC behave differently from other H chains (VH? Human vs mouse? Etc).

SAM-6 IgM’s µHC behaved similarly to many other γHC from various IgG clones in our laboratory using this hyper overexpression system from NRCC. This expression system is known to be one of the strongest (if not the strongest) mammalian expression systems that can sometimes drive crystallization of various overexpressed recombinant proteins intracellularly.

Instead of human vs mouse differences, I believe the difference is due to the combination of VH physicochemical difference and sheer difference in overexpression level. Some “privileged” HCs do not aggregate into Russell body-like detergent-insoluble aggregates even expressed in this expression platform----so the VH difference definitely plays an important role. In this sense, it is important to use equivalent expression platforms when we compare different IgMs or different VH. I added this comment briefly in the corresponding area of the manuscript.

After all, the difference can be caused by the use of different cell hosts, assays, or expression systems, so it is worthwhile re-evaluating the known effects of each mutant anew in the new expression system that can push the protein expression to a higher level.

-p20 line 453. The authors describe these ‘cytoplasmic puncta’, without characterizing them. In the discussion, they raise the possibility of them being ER exit sites. These structures should be characterized, using additional markers, ideally by CLEM.

We performed additional co-staining experiments with representative organelle markers including SEC13, SEC16A, SEC31A, BiP, calnexin, giantin, p230, NPC1, and LAMP2. The pertinent text is modified in the corresponding results section. Despite the superficial similarities to the ER exit site, the co-staining experiment did not reveal the identity of the IgM puncta by IF microscopy.

Regrettably, CLEM capacity is not available in our organization. Even a conventional transmission EM is not possible. As such, we need to save this approach for a future study. Given that a few other human IgM clones also localized to the similar puncta using our overexpression platform, the punctate localization was not a unique property of SAM-6 IgM and is worthwhile investigating more in the future.

-pages 20-21; the authors go a long way to refute some ‘influential papers’ published on the biogenesis of RBs. In Ref. 69, Mattioli et al. use HeLa transfectants to show a role of ERGIC in catalyzing the condensation of murine µHC∆CH1 chains, possibly by increasing the local concentration above a critical point. They also show a role of L chains in determining the site of condensation (smooth RBs in ERGIC, no L chains and rough RBs, ribosome coated ER, with L chains). A reasonable interpretation was that the tendency of CL domains to form homodimers facilitate condensation. Accordingly, a VL domain alone did not induce rRB formation. Would expression of a VL only suffice to change the phenotypes (see also p25)?

We already removed these lengthy arguments.

We though this is an interesting experiment to run, and we made a VL-only construct and tested how the VL only construct behaves (in terms of secretion and subcellular localization) and if VL-only co-expression would prevent the Russell body-like aggregation of μHC-ΔCH1 or μHC. The results are shown in Suppl. 4 and mentioned in the corresponding Results section.

As the reviewer may be aware, the VL-only protein can be highly condensation prone as often reported in myeloma-associated Fanconi’s syndrome patients. My initial instinct was that VL protein itself aggregates into Russell body-like inclusions and the VL protein would NOT be secreted because of its misbehaving properties. To my surprise, the VL protein was secreted well and readily detectable on Coomassie blue staining in the culture media. However, the available anti-lambda chain polyclonal antibodies are directed to the constant domain; the VL protein was invisible by Western blotting or IF imaging.

Nonetheless, the VL protein exerted its effect in the co-transfection experiments. (1) The VL-only domain was not good enough to prevent the aggregation of full-length μHC (Suppl. 4D), but (2) the VL-only protein was able to suppress the Russell body-like aggregation of μHC-ΔCH1 to the same extent as that of full-length λLC.

The authors should consider that not only cell-specific factors (ERGIC53, ERp44, the redox pose of the ER etc., see Ronzoni et al. 2010), but also subtle determinants in other domains, mainly the Variable ones, would dictate the rate, extent and localization of condensation/precipitation. These points should be further analyzed, as the differences between experimental systems may reveal novel phenomena, or at least constructively discussed.

Our model human IgM SAM-6 is germline-encoded without affinity maturation in the VL or VH region. This fact, in addition to the use of different experimental systems and different IgM model sequences, may also influence the observed differences in protein behavior as somatic mutations can change the antibody’s physicochemical properties.

Wherever appropriate, we suggested that unique physicochemical properties embedded in VL and VH of individual antibody clones and the differences in employed expression systems can play roles in revealing unexpected novel phenomena.

This is, in fact, the importance of re-visiting known knowledge of IgM assembly in the new light of a new expression platform and a different set of assay methods.

-P21, line 462. The potential role of JC in favoring µHC-∆CH1 is of interest. Considering its tendency to form intra- and inter-chain bonds (see the smear secreted by HEK transfectants) JC may serve a ‘cross-linking’ role as described for L chain by Mattioli et al (2006).

We cited Mattioli et al (2006) in Discussion (section 4.4) in the suggested context of Fig. 8F (first row).

-p25, line 555. The authors should consider expressing a VL alone or swapping VH domains. What is their role in catalyzing aggregation?

We made a VL-only construct and tested how the VL only construct behaves (in terms of secretion and subcellular localization) and if VL-only co-expression would prevent the Russell body-like aggregation of μHC-ΔCH1 or μHC. The results are shown in Suppl. 4 and mentioned in the corresponding Results section.

-p27, line 597. It is of interest that JC alone did not form RBs but was trapped in ‘RB-like’ structures when µHC were there. Are JC degraded more rapidly when not trapped in RBs?

We did not determine the intracellular half-life of JC when it is not forming RB-like aggregates or when it was trapped to RB-like structures in the presence of μHC.

Instead, we determined the detergent solubility of intracellular pool when JC was expressed alone and when JC was co-expressed with μHC or μHC mutants (see Suppl. 5B, left most panel). JC completely partitioned to soluble fraction when it was expressed alone; while the JC portioned to particulate fraction when it was co-expressed with μHC. The detergent solubility of JC was partially restored when the μHC had C575S or C414/575S mutations.

If I understand it correctly, the soluble JC in the ER should be a better ERAD substrate than the particulate insoluble JC trapped in the RB-like aggregates.

-p35 line 761. The nature and significance of these droplets should be explained and discussed.

In the absence of JC, μHC (C414/575S) aggregates into an RB-like inclusion while μHC-ΔCH1 (C414/575S) fills the ER and Golgi. In the presence of the JC subunit, both μHC (C414/575S) and μHC-ΔCH1 (C414/575S) induce protein droplets. Because Cys-575 is mutated, the JC’s cross-linking functions do not operate in this protein-protein interaction. So, their interactions must be non-covalent in nature.

Similar droplets were produced on multiple occasions in our laboratory when we overexpressed IgGs or their LC subunits. In one case, intra-ER protein droplet was induced by “scFv-Fc” fused to the 18 aa μ-tailpiece. This molecule showed cryoglobulin characteristics, and the solution became turbid at temperatures lower than 37°C and formed protein droplets by LLPS. We did not see such coprecipitation phenomena for μHC (C414/575S) and μHC-ΔCH1 (C414/575S), but we have not investigated the roles of LLPS in the presence or absence of JC. In order to study LLPS events in vitro, we have to purify all the components and test if we can reconstitute protein droplet formation only in the presence of titrating amount of purified JC.

-p36 line 791. Why is the JC smear no longer visible?

The short answer is that the JC smear is there, but the JC protein level was too low to show up as smears in lanes 2, 3, 5, and 6 of Fig. 8D. The detection sensitivity of monoclonal anti-JC mAb can also vary depending on whether JC is reduced, whether JCs are daisy chained under non-reducing conditions and whether the JC is covalently incorporated into the pentameric IgMs.

The smear might have shown up strongly if we used more sensitive mAb, if any, or some polyclonal antibodies.

Fig. 8A shows the validation of protein expression and secretion for three subunits. The proteins were analyzed under reducing conditions. The bottom panel shows JC protein expression in cell lysates (lanes 1–6) and JC secretion in culture media (lanes 7–9). The secreted JC is detected in lane 7 (Fig. 8A, bottom panel), and the secretion is blocked in lane 10 due to RB formation; while in all other lanes, the secreted JC is only faintly detectable at this exposure. The amount of released JC is pretty low in lanes 8, 9, 11, and 12 to begin with. Now, if we analyze the same culture media under non-reducing conditions, the results are shown in panel D. The expectation would be we see high molecular weight JC in lane 1 and complete absence of JC signal in lane 10, while in other lanes we should see some faint smears. But these smears are not readily seen in this blot—because the JC amount was insufficient for detection.

-p37 line 825. There are many things that one does not realize when submitting a manuscript. Papers are published if they contain original and important observation(s), like for instance that the absence of the BiP-binding CH1 is generally sufficient to induce intracellular condensation of immunoglobulins. The original observations are then confirmed (e.g. the Bruggeman lab showing that mice lacking L chains have Mott cells plenty of Ig lacking a CH1) and extended by the observation that J chains are trapped in µHC and µHC-∆CH1 ‘RBs’. Are JC detergent insoluble? Are JC trapped in IgG-containing RBs?

I performed a detergent solubility assay for JC by itself and with μHC and mutants or LCs to address this reviewer’s question. Please see Suppl. 5B.

A potentially interesting point to follow up would be to determine whether µHC form mixed polymers with µHC-∆CH1 and are trapped in RBs. It remains possible that the two end up in different blobs.

Interesting experimental suggestions. Differential detection of these two molecules (µHC and µHC-∆CH1) inside of a same cell by imaging seems technically challenging unless each protein is differentially tagged or labeled. For example, μHC can be fused to mono-GFP and μHC-ΔCH1 to mono-RFP. However, this type of large fusion partner can easily change the intrinsic properties of respective proteins and may not recapitulate the behaviors of µHC and µHC-∆CH1 precisely.

Alternatively, if there is such thing as a μCH1-specific monoclonal antibody, such a probe might make this experiment more feasible. For example, after co-expressing μHC and μHC-ΔCH1, we can stain the cells with anti-μHC antibody to detect RBs made of both μHC and μHC-ΔCH1, while the staining with anti-μCH1 monoclonal mAb will only detect the RB made of full-length μHC. This can tell if the full-length μHC and μHC-ΔCH1 are blended within a single Russell body or segregated into different blobs witin a cell.

-p38 line 827. The authors state ….it was clear that covalent aggregation between JC and µHC-∆CH1 ... was the underlying…… the logic of this conclusion is not clear. As the authors elegantly confirm, JC are not needed to form RBs.

What I meant was:

[μHC-ΔCH1 alone] = RB

[μHC-ΔCH1 (C575S) alone] = RB

[μHC-ΔCH1 (C414/575S) alone] = ER

[μHC-ΔCH1+λLC] = puncta ----- RB is prevented by λLC

[μHC-ΔCH1 (C575S)+λLC] = puncta ----- RB is prevented by λLC

[μHC-ΔCH1 (C414/575S)+λLC] = ER-Golgi

[μHC-ΔCH1+JC] = RB (JC co-aggregated in RB)

[μHC-ΔCH1 (C575S)+JC] = RB (JC excluded from RB)

[μHC-ΔCH1 (C414/575S)+JC] = droplet

[μHC-ΔCH1+λLC+JC] = RB (JC co-aggregated) -- adding JC to [μHC-ΔCH1+λLC] recalled RB

[μHC-ΔCH1 (C575S)+λLC+JC] = ER-Golgi

[μHC-ΔCH1 (C414/575S)+λLC+JC] = ER-Golgi

In the 3-chain transfection setting (show in blue), the presence of intact Cys-575 was required for RB formation because introducing C575S (shown in pink) prevented the RB formation of the 3 participating chains. For me, the logical conclusion was that in the 3-chain participating RB, the covalent crosslinking of Cys-575 and JC was the underlying mechanics for RB formation.

-p40 line 875. Sec MALS or sucrose gradient analyses should be described here to sustain the case. By the way, that the inter H-L bond is not necessary was known for 5 decades.

I feel the clear enough gel mobility difference and the increase in free LC monomer in non-reducing SDS were sufficient. In addition, in Fig. 14, we performed a series of analytical SEC assays to support our conclusion for λLC-ΔCS’s association with μHC. A reliable SEC-MALS assay would require purified higher concentration proteins, whereas a simple analytical SEC can be done on harvested culture media directly.

Yes, the unnecessary inter H-L bond has been known for a few decades for certain immunoglobulin subclasses and from different parts of animal kingdoms, as I already discussed in section 4.1.

-p42 line 912. Can the authors exclude that SS reshuffling occurs after secretion.
An interesting endeavor -also considering the long discussion about the potential medical use of IgM variants- would be to compare the stability of the mutants with or without interchain bonds.

I cannot exclude the possibility that S-S reshuffling occurs after secretion, so I included one line stating that possibility in the text. It is known to occur rather frequently in human IgG4 to generate asymmetrical bi-specific IgG4 after secretion.

I removed the entire discussion related to the medical use of IgM variants because it sounded like a “grant proposal.” But, yes, when we try to develop such molecule types for clinical use, we extensively evaluate their chemical and physical stability in an extensive series of processes called “molecular assessments” and “manufacturing assessments.” The stability of mutants with or without the H-L interchain bonds will be rigorously tested and reported to authorities when applying for medical use.

-p52 line 1133. The data suggest that unassembled µHC avoid BiP quality control and are secreted. How do the authors explain this unexpected finding?

Yes, fee μHC (4×C>S) mutant protein was secreted to the culture media in the absence of LC. Because it still has the near intact CH1 domain (except for C137S mutation), this molecule can still be a substrate of BiP mediated ER retention mechanism. This is indeed unexpected.

One potential experiment would be to create a μHC (3×C>S) construct that has intact Cys-137 residue and test if this mutant is secreted in the absence of LC. This will at least address if the BiP retention mechanism is relying on the Cys-137 to assess the secretion readiness.

I will mention that it is unexpected and that it is not understood how free μHC (4×C>S) escapes the BiP retention mechanism.

Some sections of the discussion sounds more like parts of a grant. Although probably tenable, some speculations are not sustained by data or elements that derive from this paper and could be deleted.

We removed the corresponding text completely in the revised manuscript.

Minor points

-p16 line 365. Hexamers represent the vast majority of secreted IgM. Wouldn’t ELISA allow to quantify them?

This segment was removed from the manuscript as part of the effort to shorten the manuscript. However, yes, an ELISA assay can be developed to quantify IgM hexamers. If it is already available off-the-shelf from a vendor, we could use such an assay.

We usually end up purifying the protein and quantitate them by OD280 and extinction coefficient.

-p17, lines 375 and following. Many previous studies showed that IgM can be assembled and secreted by non-lymphoid cells. Of interest, HeLa variants were isolated that failed to do so and secreted also non polymeric IgM subunits. Retention of intermediates was partially rescued by ERp44 and ERGIC53 overexpression, suggesting a defect in distal quality control. This is another example of how different cell sublines handle proteins differently. The unexpected can lead to novel findings, without the need to think that others made mistakes.

I totally agree with the view. Perhaps I was trying hard to make sense of all the conflicting information.

All the texts that sounded like refuting the conflicting results are removed in the revised manuscript.

-p19 line 423: the sentence ‘…induces a spontaneous…’ sounds somehow contradictory

I agree. I already did a major revision based on comments from both reviewers. By the time I reached this suggestion, this sentence had already been removed from the text.

-p23, line 500 and following. The dual role of CH1-BiP and C575-ERp44 was shown over 30 years ago and confirmed in many further studies.

Yes, I am aware of that. Our study is built on the foundation of many pioneering discoveries during the past 30 years from renowned investigators. Our intension is to move the needle just a little further by revealing unexpected or novel aspects of IgM biosynthesis using our expression system.
